# Comparative chromatin accessibility upon BDNF stimulation delineates neuronal regulatory elements

**DOI:** 10.15252/msb.202110473

**Published:** 2022-08-23

**Authors:** Ignacio L Ibarra, Vikram S Ratnu, Lucia Gordillo, In‐Young Hwang, Luca Mariani, Kathryn Weinand, Henrik M Hammarén, Jennifer Heck, Martha L Bulyk, Mikhail M Savitski, Judith B Zaugg, Kyung‐Min Noh

**Affiliations:** ^1^ Structural and Computational Biology Unit, European Molecular Biology Laboratory (EMBL) Heidelberg Germany; ^2^ Faculty of Biosciences Collaboration for Joint PhD Degree between EMBL and Heidelberg University Heidelberg Germany; ^3^ Institute of Computational Biology Helmholtz Center Munich Oberschleißheim Germany; ^4^ Genome Biology Unit European Molecular Biology Laboratory (EMBL) Heidelberg Germany; ^5^ Division of Genetics, Department of Medicine Brigham and Women's Hospital and Harvard Medical School Boston MA USA; ^6^ Department of Pathology Brigham and Women's Hospital and Harvard Medical School Boston MA USA

**Keywords:** enhancers, gene expression, neuronal stimulation, post‐mitotic neurons, transcription factors, Chromatin, Transcription & Genomics, Neuroscience

## Abstract

Neuronal stimulation induced by the brain‐derived neurotrophic factor (BDNF) triggers gene expression, which is crucial for neuronal survival, differentiation, synaptic plasticity, memory formation, and neurocognitive health. However, its role in chromatin regulation is unclear. Here, using temporal profiling of chromatin accessibility and transcription in mouse primary cortical neurons upon either BDNF stimulation or depolarization (KCl), we identify features that define BDNF‐specific chromatin‐to‐gene expression programs. Enhancer activation is an early event in the regulatory control of BDNF‐treated neurons, where the bZIP motif‐binding Fos protein pioneered chromatin opening and cooperated with co‐regulatory transcription factors (Homeobox, EGRs, and CTCF) to induce transcription. Deleting cis‐regulatory sequences affect BDNF‐mediated Arc expression, a regulator of synaptic plasticity. BDNF‐induced accessible regions are linked to preferential exon usage by neurodevelopmental disorder‐related genes and the heritability of neuronal complex traits, which were validated in human iPSC‐derived neurons. Thus, we provide a comprehensive view of BDNF‐mediated genome regulatory features using comparative genomic approaches to dissect mammalian neuronal stimulation.

## Graphical abstract

Dynamic multi‐omic profiling and comparative genomics are used to dissect the role of BDNF on neuronal chromatin. A comprehensive description of BDNF‐specific regulatory features is provided, from transcription factor interactions to complex traits.

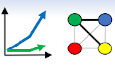

## Introduction

The brain‐derived neurotrophic factor (BDNF) plays a role in neuronal growth, survival, differentiation, repair, maturation, activity‐induced synaptic plasticity, and memory formation (Park & Poo, 2013; Panja & Bramham, 2014; Leal *et al*, 2015). BDNF is a major source of neuronal stimulation, being synthesized and secreted in the central nervous system. BDNF‐related molecular pathways are also potential targets for treating brain diseases (Choi *et al*, 2018; Nagahara *et al*, 2009) as impairments of BDNF‐mediated cellular function are linked to several neurological and psychiatric disorders (Björkholm & Monteggia, 2016; Lima Giacobbo *et al*, 2019). Exogenous BDNF application in cultured cortical neurons (CNs) mimics many of the *in vivo* effects of BDNF, from generating activity‐dependent cellular signals to changing the spine morphology associated with long‐term potentiation, thus much is known about these processes (Park & Poo, 2013; Panja & Bramham, 2014; Leal *et al*, 2015). Extracellular BDNF binds its cognate TrkB receptor (Soppet *et al*, 1991) and induces signal transduction pathways involved in phospholipase Cγ, phosphatidylinositol 3‐kinase, and mitogen‐activated protein kinase (MAPK), leading to the activation of transcription factors (TFs) such as the cAMP‐responsive element‐binding TF, early growth response factors (EGRs), and FOS transcription factor (Calella *et al*, 2007; Minichiello, 2009; Esvald *et al*, 2020). While the genomic distribution of BDNF‐induced TFs is poorly characterized, collective actions of these TFs at promoters and enhancers may coordinate gene expression required for long‐lasting structural and functional changes in neurons.

Chromatin regulation is a critical initial step in gene expression. While BDNF‐induced chromatin regulation and gene expression have been studied in individual loci (Alder *et al*, 2003; Tuvikene *et al*, 2016), genome‐wide responses to BDNF stimulation have not been analyzed. It is also unclear if the BDNF‐induced chromatin changes are related to specific brain disorders and if changes in chromatin features induced by BDNF differ from other stimulatory events. Comparative analysis of epigenomic data has helped define the role of regulatory elements in various cell types (Boix *et al*, 2021), yet it has not been applied in neurons with BDNF.

For a comparative analysis of chromatin responses, neuronal stimulation induced by an elevated level of extracellular potassium chloride (KCl) is appealing for several reasons. While BDNF stimulation activates mainly the MAPK signaling pathway, KCl stimulation induces membrane depolarization and intracellular calcium rise (Greer & Greenberg, 2008), which triggers a series of calcium‐dependent signaling events resulting in activation of TFs in the nucleus. KCl stimulation has been used to study activity‐dependent gene expression and chromatin response in primary neurons (Kim *et al*, 2010; Kitazawa *et al*, 2021) and revealed crucial TFs. The TFs transcriptionally activated by KCl include neuronal PAS domain protein 4 (NPAS4), FOS, and EGRs (Yap & Greenberg, 2018), many of which overlap with BDNF stimulation (Joo *et al*, 2016; Liu *et al*, 2018).

Here, we profiled genome‐scale changes in chromatin accessibility and transcription in mouse primary CNs following stimulation by BDNF or KCl. Our data analyses revealed changes in chromatin accessibility and the impact of these changes on gene expression in response to BDNF, compared to depolarization by KCl. Verifying our analyses with proteomics, genome‐editing, and human‐induced pluripotent stem cells (hiPSCs)‐derived CNs provide a clearer systemic understanding of stimulation‐induced chromatin response in neurons. Our results also underscore that stimulation‐induced chromatin signatures in human non‐coding regulatory regions are enriched for brain disorder‐associated loci.

## Results

### BDNF triggers diverse transcriptional responses

**Figure 1:**
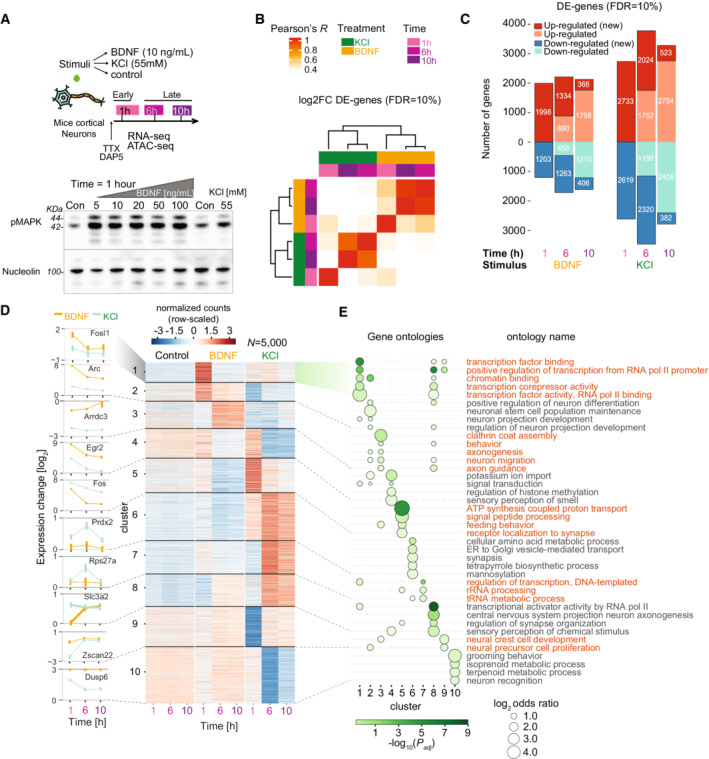
Transcriptional dynamics in mouse cortical neurons (CNs) upon neuronal stimulation with brain‐derived neurotrophic factor (BDNF) and potassium chloride (KCl) defines early and late gene programs A
*(top)* Experimental design. Cultured CNs are stimulated with BDNF, KCl or no treatment (Control) and stimulated neurons are prepared for RNA‐seq and ATAC‐seq at three specific timepoints: early (1 h) and late (6 and 10 h); *(bottom)* Blot for pMAPK in neurons treated with KCl 55 mM and several concentrations of BDNF (Con = no stimulation). 44 and 42 kDa bands indicated with lines. Nucleolin is shown as internal control.B
Hierarchical clustering for all differentially expressed‐genes (DE‐genes, adjusted *P*‐value < 0.1, using Benjamini–Hochberg's correction) using correlation of log2‐fold changes when compared to unstimulated control samples at each timepoint.C
Number of DE‐genes at each timepoint and treatment combination (above and below zero indicates up‐ and down‐regulated genes, respectively). Darker shades indicate genes newly up‐/down‐regulated at a given timepoint (new).D
Partitioning around medoids clustering of expression dynamics using genewise‐scaled Z‐scores of top 5,000 DE‐genes from (c) with lowest adjusted *P*‐value (*k* = 10 clusters). Numbers indicate clusters. Left line plots indicate expression levels for signature genes selected for each cluster. 4,907 DE‐genes are also selected with FDR = 1% and more than 100 counts across samples.E
Gene ontology (GO) enrichment analysis for clusters shown in (d). Each ribbon shows clusters with their respective significant gene groups using topGO (Alexa *et al*, 2006). Up to five significant terms per cluster are shown (*P*‐values adjusted with BH). Circle sizes indicate enrichment of ontology genes in each cluster versus all other clusters. Names on the right y‐axis indicate ontology common names.

We monitored chromatin and transcription changes across multiple timepoints after BDNF stimulation in mouse primary CNs and compared them to KCl depolarization (Fig 1A). For KCl depolarization, we applied a concentration (55 mM) of KCl that is used to induce neuronal stimulation (Tyssowski *et al*, 2018). For BDNF stimulation, we applied a physiological dose (10 ng/ml) previously reported in the mouse brain (Yoo *et al*, 2016). Neurons treated with KCl modestly activated phosphorylated MAPK (pMAPK), whereas different concentrations (5–100 ng/ml) of BDNF strongly induced pMAPK (Fig 1A). Both stimuli induced transcription of immediate early genes (IEGs) within minutes of treatment, but the transcriptional levels constantly increased with different dynamics in the first hour of stimulation (Appendix Fig S1A), in agreement with previous reports applied KCl stimulation (Guzowski *et al*, 1999; Tyssowski *et al*, 2018). Therefore, to access early transcriptional responses, we examined 1 h after treatment as the first timepoint, followed by 6 and 10 h to capture later transcriptional events (Tyssowski *et al*, 2018).

Using mRNA‐sequencing (RNA‐seq), we identified the transcriptional changes in neurons collected at 1, 6, and 10 h after BDNF stimulation, compared to unstimulated controls (two biological replicates for each time and condition). Transcriptome profiles were highly reproducible in biological replicates (Appendix Fig S1B and D). Hierarchical clustering of differentially expressed genes (DE‐genes; FDR = 10%) in all conditions revealed a clear separation of BDNF‐induced transcriptional changes compared to KCl‐induced changes, with each stimulus inducing an early (1 h) and late (6, 10 h) transcriptional response (Fig 1B). Comparison of the DE‐genes with published RNA‐seq in KCl‐treated neurons (1, 6 h) (Ataman *et al*, 2016) showed a higher correlation with the KCl samples (Spearman's *ρ* = 0.83/0.82 at 1/6 h) than BDNF (0.76/0.21 at 1/6 h; Appendix Fig S1C) validating our cell assays. Large numbers of DE‐genes were observed at all times after stimulation, but the number of genes that became newly differentially expressed at 6 h or at 10 h almost always decreased in both KCl (*N =* 3,128/3,505/261 for 1, 6 and 10 h, respectively, FDR = 1%) and BDNF (*N* = 1,786/1,388/444, FDR = 1%) (Fig 1C; Appendix Fig S3A). Thus, while more DE‐genes appeared in KCl than BDNF, both induced comparable transcriptional dynamics. The majority of DE‐genes expressed at 10 h were already differentially expressed at 6 h, indicating stable expression of the late response genes.

Neuronal stimulation induces IEGs, enriched for TFs, and delayed response genes associated with synaptic plasticity and neuronal function (Flavell & Greenberg, 2008; Tyssowski *et al*, 2018). To identify BDNF‐induced genes, we performed unsupervised clustering of the top 5,000 significant DE‐genes across stimuli and timepoints, obtaining early (1 h) and late (6/10 h) clusters across stimuli (Fig 1D). Of these 5,000 DE‐genes, 4,910 had more than 100 counts and 4,907 had an adjusted *P*‐value of 0.01 or less. Among four clusters of early up‐regulated genes, cluster 1 (e.g., Fosl1) and cluster 2 (Arc) were specific for BDNF, cluster 4 (Egr2) was increased in both stimuli, and cluster 5 (Fos) was more highly expressed in KCl. Delayed up‐regulated genes specific for BDNF contained signaling‐linked genes (e.g., Arrdc3, Cebpb; cluster 3) while those specific for KCl contained solute transporters and ion channel‐related genes (Slc3a2, Cacna1d, Slc25a25, Kcne4, etc.; clusters 6, 7, and 8). Only a few clusters of down‐regulated genes were seen in BDNF (cluster 8) and KCl (clusters 9 and 10). Gene ontology (GO) enrichment analysis showed that clusters of early BDNF‐induced genes were enriched for TFs, DNA‐binding, and transcriptional regulation‐related processes as seen previously (Flavell & Greenberg, 2008; Tyssowski *et al*, 2018) (Fig 1E). Delayed gene clusters showed enrichment of different GO neuronal terms. For example, axonogenesis and neuron migration appeared in BDNF (cluster 3), and many genes related to signal peptide processing, endoplasmic reticulum transport, and sensory perception were increased in KCl, but not in BDNF (clusters 6 and 8). Genes down‐regulated by KCl but modestly up‐regulated by BDNF (clusters 9 and 10) were enriched for neuronal cell division, neuronal recognition, metabolic process, and behavioral terms. Thus, BDNF and KCl stimulation separately trigger a transcriptional response similar in dynamics but involving different sets of genes, which could be in part mediated by variable expression of early induced TFs activating different sets of late response genes.

### BDNF alters regulatory‐element chromatin

**Figure 2:**
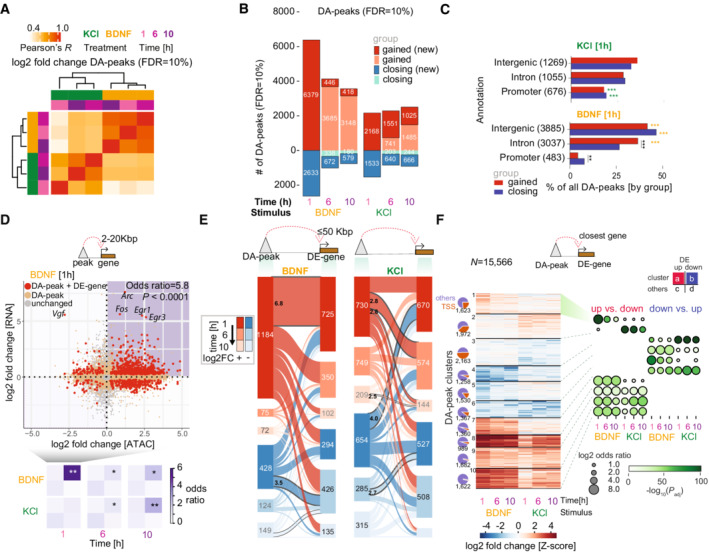
Chromatin accessibility changes upon neuronal activation with brain‐derived neurotrophic factor (BDNF) and potassium chloride (KCl) reveals early BDNF regulatory control of gene expression A
Hierarchical clustering for all differentially accessible peaks (DA‐peaks, adjusted *P* < 0.1, using Benjamini–Hochberg's correction) using correlation of log2‐fold changes when compared versus matched control samples.B
Number of DA‐peaks at each timepoint and treatment combination (above and below zero indicates gained and closing DA‐peaks, respectively). Darker shades indicate peaks newly gained/closing at a given timepoint (new).C
Percentage of gained and closing DA‐peaks grouped by top genomic annotations at the 1 h timepoint (by percentage). Numbers in parenthesis next to each annotation label indicate the absolute number of peaks associated with the annotation. Gray asterisks indicate within‐stimulation Fisher's exact test comparisons. Green/yellow asterisks indicate between‐stimulation comparisons, wherever one peak set is significantly enriched for one annotation (**P < 0.01; ***P < 0.001).D
(*top*) Association between DREs and gene expression at the BDNF 1 h timepoint. Each point indicates the log2‐fold change of an ATAC‐seq peak (x‐axis) and the gene expression of the closest gene (y‐axis) with distance between 2–20 Kbp. Colors indicate whether none (gray), only the peak (orange), or both peak and gene (red) show significant changes versus control neurons. (*bottom*) Enrichment for paired DA‐peak and DE‐gene in the four quadrants is summarized for BDNF and KCl. Asterisks indicate *P‐*values as corrected by a Benjamini–Hochberg procedure (*P < 0.05; **P < 0.01).E
Number of associations between DA‐peaks and DE‐genes for KCl (left) and BDNF (right). Sankey plot shows closest DE‐genes with distances < 50 kbp to DA‐peaks. Numbers in connecting areas between peaks and genes indicate association Z‐scores between DA‐peaks and DE‐genes (shown only if one is significant using Fisher's exact test (adjusted *P* < 0.1) and Z‐scores greater than 2.5), using a permutation approach for DA‐peaks and their connected DE‐genes while maintaining timewise changes (see Materials and Methods).F
(*left*) Partitioning around medoids clustering of accessibility dynamics using scaled Z‐scores for log2 fold changes of all DA‐peaks in (b) (*k* = 10 clusters). Venn diagrams indicate the proportion of peaks in each cluster associated with TSS or any other regulatory region (*right*) Enrichment of up‐regulated (DE‐up) versus down‐regulated (DE‐down) DE‐genes (up vs. down), and enrichment of DE‐down versus DE‐up genes (down vs. up) in closest genes within a cluster when compared with the same category in other clusters. Enrichments are calculated using Fisher's exact test, with adjusted *P*‐value correction via Benjamini–Hochberg.

To understand the regulatory basis of BDNF‐ and KCl‐induced gene expression, we quantified chromatin accessibility dynamics in the same samples (Fig 1A) using the assay for transposase‐accessible chromatin sequencing (ATAC‐seq) (Buenrostro *et al*, 2015). Principal component analysis showed that variability in chromatin peaks was reproducible within biological replicates (Appendix Fig S2A). We identified a total of 58,724 peaks, of which 15,566/6,052 (FDR = 10% or 1%) were differentially accessible (DA‐peaks) across any condition. Clustering of DA‐peaks showed a distinct separation between stimuli, but unlike the DE‐gene results, chromatin response upon BDNF induction was not clearly separated into early and late responses (Fig 2A). We separated DA‐peaks as gained DA‐peaks (increased chromatin accessibility compared to control) and closing DA‐peaks (decreased chromatin accessibility compared to control), and classified them by the time of their first occurrence. At 1% FDR, we found that the majority of all gained DA‐peaks (4,074; 90.2%) in BDNF appeared at 1 h and nearly half of them maintained increased accessibility at later timepoints (Fig 2B; Appendix Fig S3B). Only 269 (5.9%) and 176 (3.9%) additional peaks appeared at 6 and 10 h, respectively. In contrast, the gained DA‐peaks in KCl were balanced across all timepoints with 508 (32.7%), 585 (37.7%), and 459 (29.6%) at 1, 6, and 10 h, respectively. For the closing DA‐peaks (FDR = 1%), a similar pattern was seen for both BDNF and KCl: decreasing numbers from early to late timepoints, and comparable fractions of newly closing DA‐peaks (491/85/50; 220/100/621 for BDNF and KCl, respectively). Thus, BDNF stimulation triggered rapid and extensive increases in chromatin accessibility.

Despite the higher number of DE‐genes at 1 h in KCl, we found over two‐fold more DA‐peaks at 1 h in BDNF (9,012) relative to KCl (3,701) (Fig 2B; Appendix Fig S2B). These results imply that BDNF stimulation may induce a regulatory remodeling of the chromatin landscape, whereas KCl has a more substantial effect on transcription. Indeed, when DA‐peaks at 1 h were subdivided into intergenic, intronic, and gene promoter regions, DA‐peaks in KCl were over‐represented in promoters (20% for KCl and 5% for BDNF; Fisher's exact test adjusted *P*‐value < 0.001; two‐sided) and BDNF peaks were enriched for intergenic regions and introns (32% for KCl and 47% for BDNF; adjusted *P* < 0.001) (Fig 2C, all annotations in Appendix Fig S2C). Chromatin state modeling (ChromHMM; Ernst & Kellis, 2012) analysis based on neuronal datasets (Su *et al*, 2017) further revealed that gained DA‐peaks in BDNF were associated with enhancers (over 10‐fold more enrichment than KCl at genic enhancers). Gained DA‐peaks in KCl were located at active transcription start sites (TSS, highest log2 fold enrichment 2.3 in KCl vs. 1.4 in BDNF) and bivalent promoters marking neuronal genes (log2 fold enrichment 1.1 vs. 0.1) (Appendix Fig S2D). Fewer differences were observed in the closing DA‐peaks except for CCCTC‐binding factor (CTCF)‐associated regions, which was significantly enriched in KCl (log2 fold enrichment 2.0, which is 2.1‐fold higher than BDNF). Thus, the two responses promote different chromatin regulatory architectures early on, with KCl affecting gene promoters and BDNF acting preferentially through regulatory elements, such as enhancers.

Based on a study linking neuronal stimulation response to the three‐dimensional conformation of the genome (Beagan *et al*, 2020), we integrated the DA‐peaks with published Hi‐C maps generated across neurodevelopment (Bonev *et al*, 2017). These Hi‐C maps do not include genomic contacts that are formed *de novo* in response to stimulation, but encompass the pre‐established loops during neurodevelopment. We observed higher correlations for loop‐associated peak pairs in CNs with respect to the ones associated with embryonic stem cells (ESCs), or unannotated (Appendix Fig S4A), suggesting a role for genome topology in co‐varying regions. Furthermore, co‐variation of enhancer accessibility and gene expression is enriched for HiC contacts at 1 h in BDNF. An equivalent analysis for KCl showed a similar trend which was, however, not statistically significant (Appendix Fig S4B). We observed higher percentages of DE‐genes linked to CN loops in BDNF 1 h (45.3%) and KCl 1 h (39.7%) versus ESC loops (26.7 and 23.4% for BDNF and KCl 1 h, respectively; Appendix Fig S4C). These percentages were also higher when comparing DA‐peaks with between CN and ESC (9.2 and 10.9% vs. 4.9 and 5.2%). The number and percentage of DE‐genes with DA‐peaks linked via CN loops is higher for BDNF 1 h (446, 20.3%) than KCl 1 h (363, 10.1%) (Appendix Fig S4C). GO analysis does not indicate terms strongly associated with DE‐genes with loops or DE‐gene/DA‐pairs with loops, and shows weaker signals in comparison with GO analyses performed using broad dynamic gene clusters (Appendix Fig S4D). Altogether, following BDNF stimulation, changes in enhancer accessibility appear to translate into gene expression changes, and these correlate with changes in the physical connectivity in the genome observed during neurodevelopment and pre‐established loops in CNs. It will be interesting to investigate the relationship of DA‐peaks and activity‐dependent *de novo* loops in future studies to understand whether DA regions may play a role in restructuring chromatin loops upon BDNF stimulation.

### Neuronal chromatin dynamics affect gene expression

We further investigated the functional relationship between chromatin accessibility changes at regulatory elements and transcription, observing a significant association at specific timepoints between gained accessibility and increased gene expression upon BDNF (2–20 Kbp from TSS, enhancers in Fig 2D; 0–2 Kbp from TSS, promoters in Appendix Fig S5A and B). The strongest association was between gained DA‐peaks and up‐regulated DE‐genes at 1 h after BDNF stimulation (odds ratio = 5.8 for enhancers; 20.8 for promoters; adjusted *P* = 2 × 10^−18^, two‐sided Fisher's exact test with BH correction). Weaker associations were observed in enhancers at 6 h and 10 h after BDNF (OR = 1.7 at 6 h; 2.3 at 10 h; Fig 2D, bottom). KCl showed no significant association between gained DA‐peaks and DE‐genes in promoters or enhancers at 1 h, but gained DA‐peaks and up‐regulated DE‐genes became increasingly associated with enhancers at later timepoints (OR = 1.6 at 6 h; 2.5 at 10 h; *P* < 0.01). This suggests that gained accessibility in promoters is linked to up‐regulation of genes at 1 h after BDNF, whereas for KCl, the gene expression outcome of gained DA‐peaks in promoters is more complex. Chromatin remodeling at distal regulatory elements (DREs) has a rapid impact on BDNF‐induced gene expression, while for KCl, it affects gene up‐regulation at 6 and 10 h. No significant association was observed between closing peaks and down‐regulated genes at each timepoint.

To examine whether early changes in accessibility prime gene expression at later consecutive timepoints, we analyzed the association between DA‐peaks (1 h, 6 h, 10 h; gained new and closing new in Fig 2B) and their nearest DE‐gene (50 kbp or less), classified peak‐to‐gene associations by the time of their first appearance, and assessed whether any pair of peak‐to‐gene classes were over‐represented (using Z‐scores based on peak label permutations; see Materials and Methods; Fig 2E). We found that gained DA‐peaks at 1 h post‐BDNF induction were significantly correlated only to genes already up‐regulated at 1 h (odds ratio = 1.2, FDR = 10%; Z‐score > 6; Appendix Fig S5C; Dataset EV2) without being correlated with newly induced DE‐genes at later times, whereas decreasing DA‐peaks at 1 h BDNF were correlated to newly down‐regulated genes at 6 h (Z‐score > 3). For KCl, in contrast, early increased DA peaks (1 h) were significantly associated with newly induced DE genes at 6 h and 10 h (odds ratio = 1.1 and 1.3, respectively; Z‐scores > 2.5). Thus, increased and decreased chromatin accessibilities at 1 h likely prime gene expression at later times in KCl and BDNF, respectively.

To further dissect the relationship between chromatin accessibility and gene expression we performed unsupervised clustering of all DA‐peaks across timepoints and conditions (KCl and BDNF), grouped them into 10 clusters (Fig 2F), and calculated whether they were enriched for peaks connected to up‐ versus down‐regulated genes at individual timepoints and conditions. A set of BDNF‐specific early response peaks associated with early response genes in BDNF (cluster 1; 1,623 peaks) and a similar peak cluster for KCl (cluster 2; 1,972 peaks) contain a large fraction of promoters. Together with cluster 1, another set of peaks (cluster 7; 1,360 peaks) includes opening peaks associated with upregulated genes in response only to BDNF, at all timepoints. Sets of shared DA‐peaks that were affected by both BDNF and KCl (clusters 8–10; 4,293 peaks) showed a faster response in BDNF than in KCl for accessibility and gene expression, and are composed mainly of distal elements. In the case of closing peaks associated with downregulated genes, we could identify four different trends (clusters 3–6; *N =* 2,163/1,258/1,530/1,367, respectively). Cluster 3 comprises KCl‐specific peaks corresponding to a great extent to promoters (in all timepoints) whereas clusters 4 and 5 are BDNF‐specific and are mostly annotated as distal elements, with cluster 5 depicting early response downregulated genes. Similar to clusters 8–10, cluster 6 represents shared closing peaks, with a large proportion of distal elements. Taken together, BDNF induces robust changes in chromatin accessibility that direct early gene expression, which persists to later timepoints. In contrast, accessibility changes appear delayed in the KCl response, and specific chromatin patterns were only enriched for late‐response genes.

### TF motifs underlying chromatin responses

**Figure 3:**
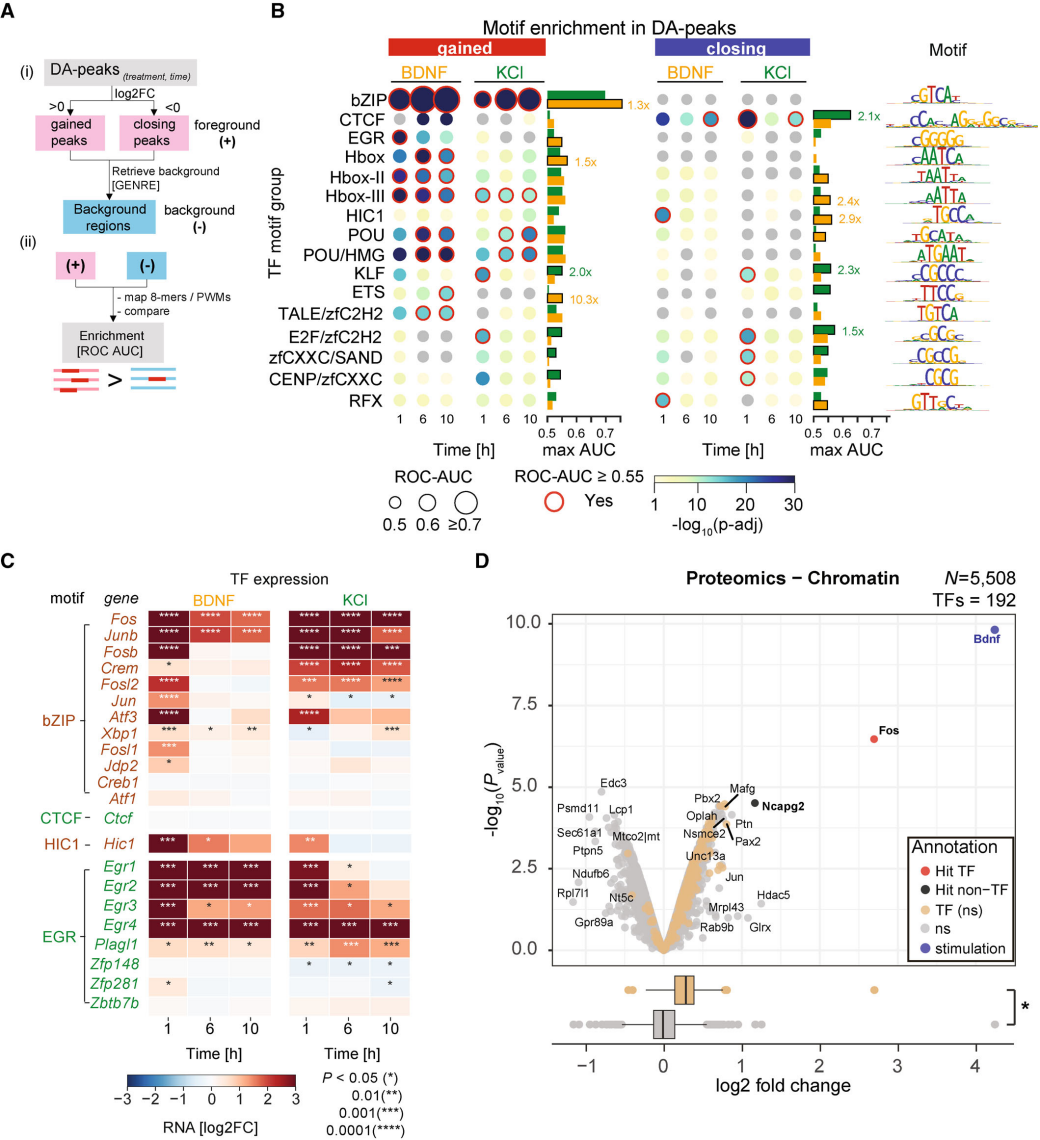
Transcription factors (TFs) linked to differentially accessible chromatin regions identify common and stimulus‐specific regulatory TFs A
(i) DA‐peaks obtained at each timepoint are analyzed with GENRE (Genomically Equivalent Negative REgions) software to generate regions used as negative controls in enrichment tests (Mariani *et al*, 2017). (ii) A library of 8‐mers and position weight matrices (both motifs) are used to classify positive sequences (DA‐peaks) versus negative control regions as a receiver operating characteristic area under the curve value (ROC‐AUC).B
Enrichment of regulatory motifs in gained (*left*) and closing (*right*) DA peaks. Circle size indicates ROC‐AUC for recovery of positive versus negative regions. Circle color indicates *P‐*value significance (one‐sided Wilcoxon rank sums test). Red lines indicate adjusted *P* < 0.1 after Benjamini–Hochberg's correction. ROC‐AUC values lower than 0.5 are shown as gray circles. Barplots indicate maximum value observed for each TF group across all timepoints for brain‐derived neurotrophic factor (BDNF) (orange bar) and KCl (green bar). In relevant cases for main text fold changes are labeled.C
RNA expression changes for TFs related to 8‐mer groups bZIP, CTCF, HIC1, and EGR. Significant changes relative to control samples are shown with asterisks (Wilcoxon rank sums test, two‐sided).D
Proteomics of the chromatin‐bound fraction from mESC‐derived neurons 1 h after stimulation show significant enrichment of TFs upon stimulation with BDNF compared to KCl (boxplot, lower panel, Wilcoxon two‐tailed rank sum test, *P < 0.0001; central band indicates the median, boxes depict third and first quartiles and whiskers show the 1.5× IQR above and below the box). Out of 5,508 detected proteins, Fos shows a significant abundance increase after BDNF stimulation compared to KCl (¦log2 fold change¦ ≥ 1, adjusted *P‐*value < 0.05). High levels of BDNF (blue dot) are associated with experimental stimulation.

Given that chromatin accessibility in distal elements was partially shared between BDNF and KCl (despite a delay in the KCl response), we explored common and specific TF activity after stimulation. To identify TF‐binding motifs in each set of gained and closing DA‐peaks, we used 8‐mers describing 108 TF specificity groups (Mariani *et al*, 2017) and a position weight matrices (PWMs) database for TF‐binding specificities (Weirauch *et al*, 2013), quantifying TF motif enrichment in comparison with mouse‐specific negative control regions (generated by GENRE (Mariani *et al*, 2017); Fig 3A; Materials and Methods). Some 69% of DA‐peaks appearing after stimulation are associated with one of the 16 over‐represented TF motifs (Appendix Fig S6A) indicating a small set of TFs dominate the activity‐dependent regulatory landscape. The majority of TF motifs reported next are still significantly detected when using DA‐peaks selected with FDR = 1% (Appendix Fig S3D). The basic leucine zipper (bZIP) domain motif showed the highest motif enrichment in gained DA‐peaks for both BDNF and KCl (Fig 3B, receiver operating characteristic area under the curve ([ROC‐AUC] = 0.65; *P* < 0.0001; Wilcoxon rank sum test, BH‐adjusted), consistent with an *in vivo* study of electroconvulsive stimulation in mouse brain (Su *et al*, 2017). The prominent effect of bZIP on opening chromatin regions was corroborated by the physical centrality of bZIP sites in gained DA‐peaks (Appendix Fig S6B) consistent with a pioneering role for these TFs (Vierbuchen *et al*, 2017; Su *et al*, 2017). Homeobox (Hbox‐III) and POU domain (POU; POU‐HMG) motifs were also enriched in gained DA‐peaks after BDNF and KCl stimulation (ROC‐AUC > 0.55). Among the BDNF‐gained peaks, we found motifs for the two Homeobox subgroups (Hbox and Hbox‐II), early growth response (EGR), ETS, and TALE/zf‐C2H2, which are mainly activator TFs. In contrast, E2F/zf‐C2H2 and KLF motifs, which function both as activator and repressor TFs, were enriched at 1 h post‐induction in KCl only, implying a more complex gene expression outcome of gained DA‐peaks at 1 h upon KCl stimulation. Furthermore, closing DA‐peaks were significantly associated with motifs for hypermethylated in cancer 1 (HIC1) and regulatory factor X (RFX) in BDNF, and with motifs for CTCF, E2F, KLF, and zf‐CXXC/SAND in KCl (Fig 3B) implying a stimulus‐specific role for these TFs.

Given TFs of the same family share DNA‐binding domains and recognize similar motifs (Weirauch *et al*, 2014; Mariani *et al*, 2017), we used RNA‐seq expression of individual TFs to refine the observed motif enrichments. Among 12 TF members related to bZIP motifs, *Fos*, *Junb*, *Fosb*, and *Fosl2* transcripts increased significantly at 1 h in both BDNF and KCl, consistent with the enrichments in gained peaks (Fig 3C). Their expression levels were reduced at 6 h in BDNF, but sustained in KCl until 10 h, possibly reflecting the delay in activation for KCl stimulation. Among the eight TF members with EGR specificities, four (*Egr‐1/2/3/4*) were induced in both BDNF and KCl at 1 h, with increased *Egr1* and *Egr2* levels continuing until 10 h in BDNF but declined at 6 h in KCl. The higher *Hic1* expression levels in BDNF relative to KCl, together with the *Hic1* motif being enriched in early closing DA‐peaks upon BDNF stimulation, are consistent with *Hic1* acting as repressor (Pinte *et al*, 2004; Boulay *et al*, 2012; Ullah *et al*, 2018). Despite high enrichment of the CTCF motif in KCl‐induced closing DA‐peaks, *CTCF* showed invariable expression levels, consistent with its ubiquitous expression and structural role in the genome (Phillips & Corces, 2009). CTCF is likely to function with other TFs whose expression was induced by stimulation.

To assess TF protein levels, we performed mass spectrometry‐based quantitative proteomic analyses on the chromatin‐bound fraction at 1 h post‐stimulation (Materials and Methods). Principal component analysis clustered the biological duplicates based on the treatments (Appendix Fig S6B). A significant increase in Fos protein abundance was observed in samples after BDNF stimulation, which was more pronounced than that in KCl (adjusted *P* < 0.2; two‐sided Wald test, BH‐adjusted) (Fig 3D, upper panel). Fos was the highest‐enriched endogenous protein among the chromatin‐bound fraction after 1 h BDNF stimulation. Among all 5,508 proteins detected, we observed an overall increase in the abundance of 192 combined TF proteins after BDNF stimulation compared to KCl (Fig 3D, lower panel, comparison between RNA and proteins in Appendix Fig S7C and D). However, the levels of the individual TFs, other than Fos, did not significantly change, possibly due to their low protein abundances. These results are consistent with the Fos protein playing a central role in neuronal stimulation related to synaptic plasticity, memory, and learning (Kandel, 2012). Our transcription and proteomics results are concordant with the bZIP motif being the most enriched in gained DA‐peaks, and support the functional role of bZIP, especially Fos protein in augmenting chromatin accessibility upon BDNF stimulation.

In addition, Fos protein showed a delayed increase in KCl treatments by Western blot (Appendix Fig S7A), and the enrichment for Fos‐associated motifs at enhancers (intergenic regions) linked to gained DA‐regions (FDR = 1%) is higher at later timepoints for KCl and earlier for BDNF (Appendix Fig S6C). These results suggest that Fos‐bound enhancers exert a delayed response to KCl. Furthermore, while Fos protein was more abundant after 1 h of BDNF treatment than in KCl, Fos mRNA showed the opposite trend, with higher levels in KCl‐treated neurons. This discordance might be due to the complex equation of Fos protein and mRNA levels. For instance, there is negative autoregulation of the fos gene upon Fos protein (Rahmsdorf *et al*, 1987; Wilson & Treisman, 1988; Schönthal *et al*, 1989), where a higher stabilization of the Fos protein via phosphorylation (Chen *et al*, 1993; Monje *et al*, 2003; Gilley *et al*, 2009) results in lower levels of *fos* mRNA (Rauscher *et al*, 1988a, 1988b).

### bZIP and TF cooperativity in induced gene expression

**Figure 4:**
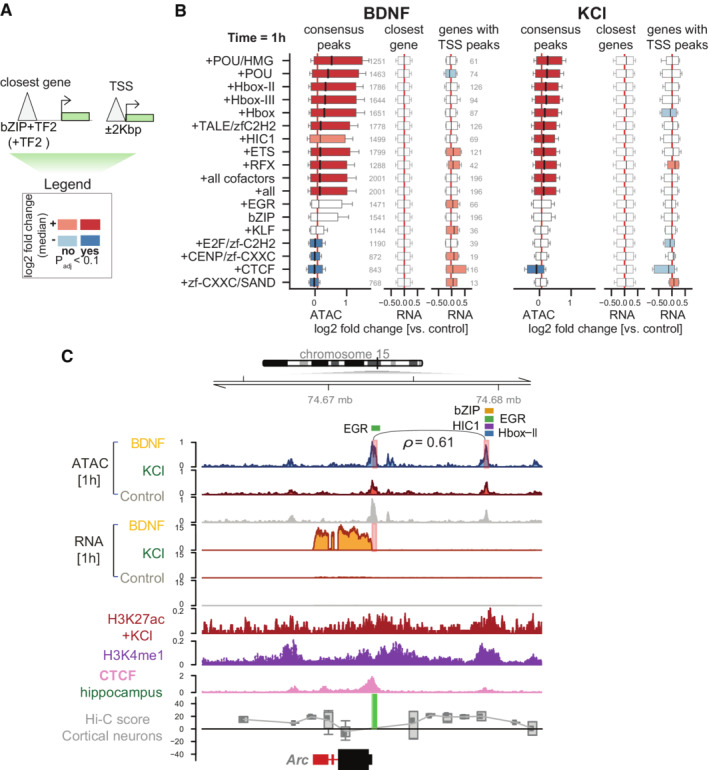
Brain‐derived neurotrophic factor (BDNF) transcriptional up‐regulation is linked to bZIP and EGR interactions in promoters and enhancers A
Scheme indicating annotation of ATAC‐seq peaks based on the presence of a bZIP motif (bZIP) and another TF (TF2). Peaks are linked to genes based on closest genomic distance (“closest gene”) or only if present in gene TSS (“TSS”). A log2‐fold change median indicates the difference in accessibility or expression log2‐fold changes between all peaks with bZIP+TF2 motifs versus only bZIP peaks, and equivalently for the expression values of genes connecting to those peaks.B
Genome‐wide changes at bZIP+TF2 and bZIP were measured as log2‐fold change distributions for accessibility (“consensus peaks”), genome‐wide closest genes (“closest gene”), and proximal target gene RNA levels (“genes with TSS peaks”) when bZIP motifs are co‐present with other TFs (TF2) in ATAC‐seq peaks versus bZIP alone. Significance is assessed using the one‐sided Wilcoxon rank sums test between peaks with *k*‐mers co‐present versus peaks with only bZIP. Absolute changes lower than 0.1 and not significant are shown as white boxplot bars.C
Chromosome 15 genome tracks neighboring *Arc*, displaying ATAC‐seq read counts per million (CPM); RNA‐seq CPM; H3K27ac normalized signal upon potassium chloride (KCl) stimulation, H3K4me1 (Malik *et al*, 2014); CTCF (Sams *et al*, 2016); and Cortical neurons Hi‐C data (Bonev *et al*, 2017). Red bars in ATAC‐seq tracks indicate gained DA‐peaks in BDNF, and red bars in RNA‐seq tracks indicate *Arc* differential expression in BDNF and KCl 1 h. Green blocks in Hi‐C tracks indicate anchor points for the calculation of contact scores, using shaman (Cohen *et al*, 2017). Line and Spearman's rho value indicate counts rank‐based correlation between highlighted peaks across all samples (Appendix Fig S4C). TF module names indicate the presence of *8*‐mers in those peaks.

Chromatin regions that open early, presumably in response to bZIP‐related factors, could potentially be amenable to other cofactors. We tested whether the co‐presence of bZIP with other TF motifs (Fig 3B) increased accessibility over the bZIP motif alone (Fig 4A; see Materials and Methods). POU, Hbox, ETS, or RFX motifs are co‐present with bZIP (bZIP+TF2) and this is associated with a significant increase in accessibility after BDNF and KCl stimulation. In contrast, EGR or KLF motifs co‐present with bZIP did not show any further changes in accessibility (Fig 4B, Appendix Fig S8A). To evaluate the collaborative effects of the co‐present sites on gene expression, we compared the change in expression for genes near peaks with bZIP+TF2 versus peaks with bZIP alone. The co‐presence of TFs such as ETS, RFX, EGR, KLF, zfCXXC, and CTCF with bZIP at proximal regions (< 2 kb from TSS) of target genes was positively associated with BDNF‐induced gene expression (Fig 4B). EGR and KLF were associated with increased expression of proximal genes upon BDNF stimulation without any additive effect on chromatin accessibility compared to bZIP alone (Fig 4B). Thus, while bZIP is the main TF responsible for chromatin accessibility, other TFs, such as POU and Hbox, further enhance accessibility without directly affecting nearby gene expression, whereas EGR and KLF may be required to fine‐tune BDNF‐induced gene expression without changing accessibility.

bZIP+TF2 co‐presence at distal elements (> 2 kb from TSS) did not show any significant effects on the expression of the closest gene, which may partially be due to the complexity of mapping distal regulatory sites to their correct target genes. Cooperation was seen between a subset of increased DA‐peaks at enhancers and up‐regulated DA‐genes (Fig 2D), where co‐occurrence of bZIP+TF2 could have an impact. To test whether the co‐occurrence of bZIP+TF2 could impact gene expression induced by BDNF stimulation, we focused on the *Arc* gene, a key effector for synaptic function (Plath *et al*, 2006; Tzingounis & Nicoll, 2006; Kawashima *et al*, 2009; Pintchovski *et al*, 2009) which is substantially induced by BDNF stimulation (Fig 4C). The distal region of *Arc* showed increased accessibility at 1 h in BDNF and contains various activator TF motifs (one bZIP, three EGR, and one Hbox‐II) and one repressor TF (two HIC1 motifs) in close proximity. This distal region exhibited properties of an active enhancer (enriched H3K27ac and H3K4me1 marks obtained from neuronal epigenomics data) (Malik *et al*, 2014), bound CTCF (Sams *et al*, 2016; Ren *et al*, 2017), and had a high Hi‐C contact score with the *Arc* gene, as measured by shaman package (preprint: Cohen *et al*, 2017). The binding of Egr1 and a bZIP family protein, Fos, to this region was also examined by ChIP‐quantitative PCR (qPCR) (Appendix Fig S9).

We hypothesized that TF motifs adjacent to bZIP in this enhancer region could contribute to higher *Arc* expression upon BDNF stimulation. To assess this, we aimed to remove one of the activator TF motifs such as EGR motif and determine the level of *Arc* induction. It is, however, complicated as each of the three EGR motifs at this enhancer region is very close to the other TF motif (first EGR motif with HBox‐II, second EGR motif with HIC1, and third EGR motif with bZIP). To this end, we chose to remove the second EGR motif with HIC1 as we might be able to separate the activator and repressor effect. The third EGR motif is in high proximity to bZIP, thus we chose not to disturb it. We generated mouse embryonic stem cell (mESC) clones that homozygously removed the distal genomic region containing one of the three EGR motifs and one or two HIC1 motifs adjacent to the EGR motif, depending on the clone, without disturbing the other motifs (Jinek *et al*, 2012; Materials and Methods, Appendix Fig S8B). We differentiated the clones into neurons and measured *Arc* expression, using RT‐qPCR, after stimulation. Similar to primary neurons, *Arc* expression increased upon BDNF stimulation in ESC‐derived neurons. We observed a significant reduction in *Arc* gene expression upon BDNF stimulation in clones with a deletion of the distal TF motifs (*t*‐stat = −3.0, *P* < 0.01; two‐sided *t‐*test), but not in CRISPR controls (*P* > 0.05) (Appendix Fig S8C; Dataset EV3). KCl treatment failed to induce Arc expression in mESC‐derived neurons, unable to verify the role of this putative enhancer in KCl‐dependent Arc expression. These results suggest that an EGR motif close to bZIP in the DRE functions in BDNF‐mediated *Arc* gene activation. In addition, when dissecting the expression levels per clonal line, we observed a trend of anticorrelation between the number of HIC1 motif deletions (additionally to the EGR motif deletion) and *Arc* transcription induced by BDNF, implying a role of HIC1 in this enhancer (Appendix Fig S8C).

Furthermore, we investigated the cooperativity between the transcription factors EGR and bZIP in the regulation of *Arc* expression. For this, we used the small molecule inhibitor T5224, which prevents AP‐1 (bZIP) family members, such as Fos, from binding DNA (Aikawa *et al*, 2008). The control and KO mESC clones bearing deletions in the distal genomic region of the Arc gene were differentiated into neurons and treated with BDNF for 1 h and increasing concentrations of T5224. As expected, the inhibitor treatment reduced *Arc* expression triggered by BDNF stimulation and also other BDNF‐inducible genes such as *Fos* and *Btg2* (Appendix Fig S8D), known to be transcriptionally regulated by AP‐1 family members (Pagin *et al*, 2021). The inhibitor treatment yielded a significantly reduced level of Arc gene expression in the KO lines compared to the control lines, suggesting the joint effect of AP‐1 (bZIP) and EGR TFs on *Arc* expression. We tested two models to describe *Arc* expression. A simple model considered *Arc* expression to be additively dependent on the EGR‐binding motif (deletion of the genomic region containing EGR motifs) and bZIP (T5224 inhibitor concentration) as in Arc ∼ EGR + bZIP. A more complex model included the interaction between bZIP and EGR: Arc ∼ EGR + bZIP + EGR:bZIP. We found that the complex model could describe our data significantly better than the simple model (Likelihood Ratio Test χ^2^(1) = 4.3438, *P* = 0.03714). As a negative control, we analyzed the BDNF‐dependent expression of *Fos* and *Btg2* in control and KO lines in the presence of T5224 (Appendix Fig S8D). The expression of Fos and Btg2 genes is better explained by a simple model relying solely on the bZIP term and not by the complex model including the interaction of two factors (*Fos*: Likelihood Ratio Test, χ^2^(1) = 2.8148, *P* = 0.2448; *Btg2*: Likelihood Ratio Test χ^2^(1) = 4.476, *P* = 0.1067). Altogether, our results validate the cooperativity of bZIP and EGR TF motifs at Arc enhancer in Arc gene activation upon BDNF stimulation.

### Differential neuronal gene exon usage

**Figure 5:**
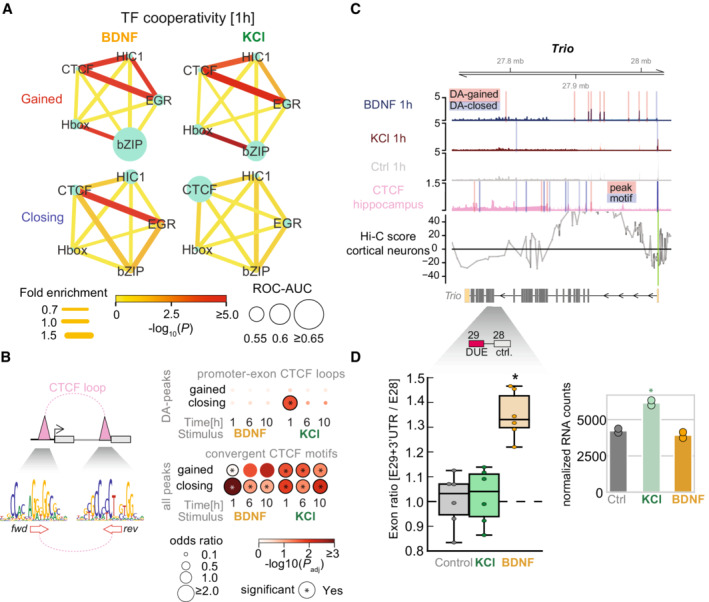
Chromatin–transcription factor (TF) interactions during mouse neuronal activation and their association with promoter‐exon loops and splicing A
Enrichment association networks between HIC1/bZIP/EGR/CTCF and Hbox based on results from (Fig 4A). Circle sizes indicate ROC‐AUC using motifs for each TF alone in those peaks. Edges weights and colors indicate fold enrichment for co‐occupied peaks versus single peaks, and significance of association. Calculations were done with SuperExactTest (Wang *et al*, 2015).B
(*top‐left*) Scheme depicting CTCF‐loop connecting promoters and exons. (*bottom‐left*) CTCF‐loops are enriched for convergent CTCF motifs. (*top‐right*) Enrichment of promoter‐exon CTCF loops in gained and closing DA‐peaks (loop annotations from Ruiz‐Velasco *et al* (2017)). (*bottom‐right*) Enrichment of convergent CTCF motifs with a distance of less than 50 Kbp in gained DA‐peaks and closing DA‐peaks over unchanged peaks as background.C
Genome tracks harboring the *Trio* gene. ATAC‐seq tracks indicate DA‐peaks (red highlight = gained DA‐peak; blue highlight = closing DA‐peak); CTCF tracks indicate the presence of motifs (pink highlight = ChIP‐seq peak; blue highlight = motif based on CTCF Position Weight Matrix). Below gene models, reference DUE exon position is highlighted (red); control exon (highlighted in gray) is used for comparison.D
(*left*) Exon ratio between E29 + 3′UTR and E28 fold changes 1 h after treatment with BDNF (orange), KCl (green), and control (gray). Central band indicates median. Boxes indicate interquartile range (IQR, or Q3‐Q1). Central band indicates median. High and low whiskers indicate first datum higher than Q3 + 1.5*IQR, or lower than Q1–1.5*IQR, respectively. Asterisk indicates significant changes versus control (*t*‐test, two‐sided; *P < 0.1). (*N* = 2, independent biological replicates). (*right*) Normalized gene counts for gene expression values versus control (*adjusted *P*‐value < 0.1 versus control).

Our TF cooperativity analysis revealed that CTCF motifs were significantly co‐localized with EGR in gained DA‐peaks after BDNF and KCl stimulation, and in closing DA‐peaks upon BDNF stimulation (Fig 5A). Most DA‐peaks contain non‐overlapping motifs for CTCF and EGR, suggesting the colocalization is not an artifact based on partial EGR and CTCF motif overlap. Closing DA‐peaks at 1 h in KCl were enriched for CTCF‐binding sites without EGR motifs, convergent CTCF motifs between proximal peaks in promoter‐exon pairs, and known CTCF promoter‐exon loop annotations (OR = 3.5; Fisher's exact test adjusted *P* < 0.001; Fig 5B). Thus, there may be a functional interaction between CTCF and EGR with looping‐associated gene regulation, which might not act in closing DA‐peaks upon KCl stimulation. Given that CTCF binding may affect exon usage (Shukla *et al*, 2011; Paredes *et al*, 2013; Ruiz‐Velasco *et al*, 2017), these insights combined with the enrichment of CTCF‐binding sites in both intronic and exonic regions of DA‐peaks (Appendix Fig S10A), suggest activity‐dependent chromatin accessibility may play a role in alternative splicing.

To test this idea, we quantified differentially used exons (DUEs) between BDNF and KCl, and found 54 genes with DUEs that harbored BDNF DA‐peaks over a CTCF motif or a putative promoter‐exon CTCF loop (OR = 1.6 relative to non‐DUE genes; *P* < 0.001; Appendix Fig S10B; promoter‐exon CTCF loop predictions from Ruiz‐Velasco *et al* (2017 *)*). We selected DUEs within three of these 54 genes (Trio, Stxbp5, Cpe‐201) whose functions were implicated in neurons (Woronowicz *et al*, 2010; Geerts *et al*, 2017; Katrancha *et al*, 2019) and validated them using RT‐qPCR. Relative exon usage was assessed by the ratio between the exon differentially used in our analysis and one control exon from the same gene that remained unchanged after stimulation. We confirmed that BDNF but not KCl increased the relative exon usage of all three genes with respect to unstimulated control neurons without changing the expression level of the genes (Fig 5C and D for Trio; Appendix Fig S10C for Stxbp5, Cpe‐201; Dataset EV3). These results suggest that BDNF stimulation increased the expression level of specific spliced mRNA isoforms of some neuronal genes, whereas KCl stimulation did not exert this effect, perhaps through promoter‐exon CTCF‐looping regulation, which was described as splicing mechanisms before (Ruiz‐Velasco *et al*, 2017). In the Trio gene DUE arises through inclusion/exclusion of exon 29, which is the last exon of a transcript variant that carries an additional 3′UTR sequence, generating a truncated protein. Given that reduced levels of full‐length Trio and the truncated mutations are linked to neurodevelopmental disorders and intellect0ual disability (Pengelly *et al*, 2016; Sadybekov *et al*, 2017), our results highlight a potential connection between BDNF‐induced chromatin dynamics and exon‐specific gene expression in neuronal disorders.

### Chromatin‐accessible regions associated with neuropsychiatric traits

To investigate the relationship between activity‐induced chromatin accessibility in neurons and human disease, we analyzed GO terms associated with the genes neighboring DA‐peaks after BDNF and KCl stimulation and found they were linked with distinct neurobiological and learning functions (Appendix Fig S11A). To determine whether these BDNF‐ and KCl‐responsive elements are involved in distinct neuropathological traits, we used data from 45 genome‐wide association studies (GWAS) that link genetic variants with complex traits of diseases (Buniello *et al*, 2019) and calculated the enrichment of trait‐associated SNPs among our DA‐peaks (transferred to the human genome) using partitioning heritability analysis (Finucane *et al*, 2015; Materials and Methods, Dataset EV4).

**Figure 6:**
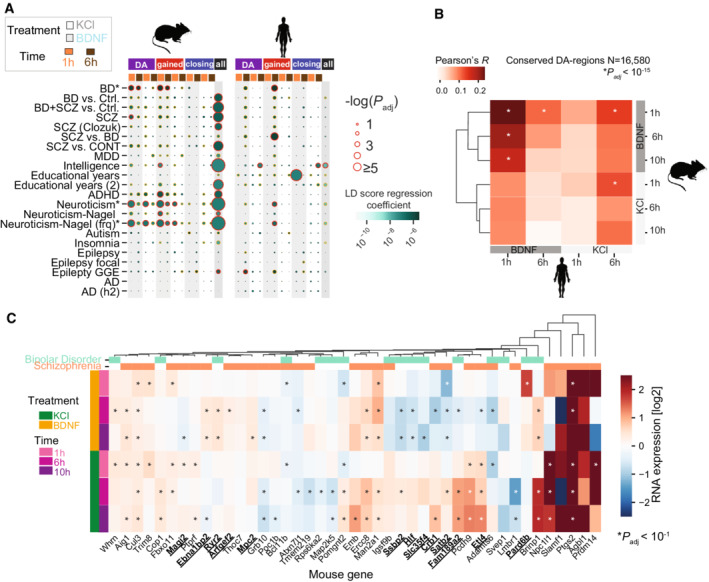
Conserved brain‐derived neurotrophic factor (BDNF)‐specific chromatin accessibility associations with human complex traits A
Associations between chromatin accessibility DA‐peaks and genome‐wide association studies (GWAS) summary statistics. All DA‐peaks (DA), gained DA‐peaks (gained), closing DA‐peaks (closing), and consensus peaks (all) are fitted to summary statistics data of multiple GWAS studies (y‐axis). Circle color indicates linkage disequilibrium score regression coefficient (effect size), and circle size indicates association significance. Shadings indicate treatments. Timepoints are shown (orange = 1 h, brown = 6 h). Red lines indicate LD score regression coefficient adjusted *P* < 0.1 after BH correction. Orange lines indicate *P* < 0.1. (BD, Bipolar Disorder; SZ, Schizophrenia; BD + SZ, BP and SZ samples combined; MDD, Major Depressive Disorder; ADHD, Attention deficit hyperactivity disorder; AD, Alzheimer's Disease). Details for GWAS studies and preparation steps in Dataset EV4.B
Correlations between differentially accessible elements conserved mouse and human regions across conditions (*N* = 16,580). Asterisks indicate significance of Pearson's *R* correlation after correction with BH‐procedure.C
Mouse expression of GWAS Schizophrenia and Bipolar Disorder human genes with DA‐regions conserved and differentially accessible in the mouse genome. Genes with significant up‐ or down‐regulation for one treatment (BDNF or KCl) versus control, and not the other are in bold and underlined (Wald test, two‐sided).

Testing for associations between all genome regions harboring ATAC‐seq peaks, we found enrichment for several neuronal traits and little enrichment for non‐neuronal traits (Appendix Fig S11B), thus validating our analysis and experimental system. We identified 31 significant GWAS signals (adjusted *P*‐value < 0.1, using BH correction) in chromatin accessible regions from mouse neurons, suggesting that conserved neuronal chromatin regions (between the mouse and human genomes) are linked to a subset of psychiatric traits (Fig 6A), consistent with previous studies showing that accessible genomic regions in mammalian brains are linked to human neuropsychiatric disorders (de la Torre‐Ubieta *et al*, 2018; Hook & McCallion, 2020).

In addition to these general enrichments, we also found several stimulation‐specific trait associations. Specifically, gained DA‐peaks after BDNF stimulation significantly overlapped with inherited risk loci for bipolar disorder (BD), Schizophrenia (SCZ), intelligence, attention deficit hyperactivity disorder (ADHD), and neuroticism (Fig 6A). Among them, neuroticism risk loci were also associated with gained DA‐peaks after KCl stimulation. DA‐peak regions after BDNF and KCl did not overlap with risk loci for autism and epilepsy, implying that a subset of neuropsychiatric traits might be particularly sensitive to chromatin dynamics upon BDNF and KCl stimulation. Alzheimer's disease (AD), a neurodegenerative trait, was not observed in our early‐stage neurons consistent with AD risk loci mainly being associated with enhancers in microglial cells (Hemonnot *et al*, 2019; Boix *et al*, 2021).

To validate the association between human neuropsychiatric traits and BDNF‐induced DA‐peaks in a different system, we generated hiPSC‐derived excitatory neurons, using doxycycline‐inducible NGN2 expression, and determined chromatin accessibility after BDNF and KCl stimulation (Materials and Methods). The hiPSCs‐induced neurons exhibited post‐mitotic neuronal markers comparable to the primary cultured neurons from mice (e.g., TUBB3, MAP2, Synapsin 1/2, and Tau; Appendix Fig S12A and B). Similar to the results from mouse neurons, hiPSCs‐induced neurons after BDNF stimulation generated more DA‐peaks than KCl, although the major response was delayed compared to mice, being observed at 6 h (Appendix Fig S12C and D). Clustering of 3,029 human DA‐peaks (FDR = 10%) revealed two separate groups by stimulation (Appendix Fig S12E). A comparison of BDNF‐induced chromatin accessibility between mouse and human neurons identified a weak but significant correlation in conserved genomic regions (Fig 6B).

Repeating our partitioning heritability analysis using the human DA‐peak regions (sorted by differential accessibility *P*‐value) revealed nine significant GWAS signals with neuropsychiatric traits (Fig 6A; Appendix Fig S11B). A strong association with educational attainment was observed only in human DA‐peaks upon BDNF stimulation. Conversely, the ADHD association found in mouse DA‐peaks upon BDNF stimulation did not appear in human DA‐peaks. However, BD, SCZ, and neuroticism were commonly associated with gained peaks after BDNF stimulation in both species but disappeared in overall accessible regions (Fig 6A), demonstrating that neuropsychiatric disorder‐related GWAS SNPs tend to be located near BDNF‐stimulation‐specific regulatory regions.

Prior work reported that individuals with BD or SCZ often show reduced levels of BDNF (Ray *et al*, 2014; Lima Giacobbo *et al*, 2019), suggesting a link between BDNF‐mediated gene regulation and these traits. Therefore, we examined whether SCZ‐ and BD‐risk loci‐linked genes show an altered response upon BDNF stimulation that was different from their response upon KCl stimulation (which was not linked to BD or SCZ in our analysis in Fig 6A). Assessing the changes in expression levels of SCD‐ and BD‐linked genes upon BDNF and KCl stimulation in mouse neurons (Fig 6C) revealed BDNF‐specific up‐regulated (Ebna1bp2, Ryr2, Arfgef2, Pard6b, and Mpc2) and down‐regulated (Ssbp2, Hlf, Slc35f4, Crb1, Satb2, Fam189a2, Etl4, and Magi2) DE‐genes, implying that the GWAS‐variants for these genes act through BDNF‐response elements. Together, these analyses suggest a connection between stimulation‐dependent chromatin accessibility and human complex traits, especially in a subset of neuropsychiatric disorders.

## Discussion

In this study, we performed a comprehensive analysis of gene expression and chromatin accessibility to define the overall principles and specificity of the response to neuronal stimulation triggered by BDNF, in comparison to neuronal depolarization triggered by KCl. Our transcriptional analyses revealed that BDNF and KCl stimulation in mouse primary CNs lead to distinct patterns of gene expression, involving early and late transcriptional waves, with differentially expressed TFs likely being responsible for the patterns of differentially expressed genes.

Transcriptional and chromatin profiling in brain neurons upon stimulation has been a challenging task given the complexity of the brain, which hinders the detection of specific signals due to high variability across cell types (Winick‐Ng *et al*, 2021). Our chromatin profiling *in vitro* culture system is presented as a tool to explore and identify candidate genomic regulatory elements limited to specific stimulation and cell type. We have validated our findings using CRISPR perturbations of regulatory elements on transcription, as well as compared chromatin responses to BDNF in mouse and human in an attempt to relate our findings to potential diseases. Nevertheless, we note that the effects on chromatin in response to stimulation are extremely specific to many parameters, such as stimulation strength and duration (Joo *et al*, 2016; Fukuchi *et al*, 2017; Tyssowski *et al*, 2018), neuronal type and connectivity (Fuentes‐Ramos *et al*, 2021; Harabula & Pombo, 2021) and possibly cellular memory to previous stimuli (Yap & Greenberg, 2018).

The proper response to BDNF stimulation can be crucial during neurodevelopment when the transcriptional program must be tightly regulated to avoid untimely consequences (Cohen‐Cory *et al*, 2010). On the other hand, neuronal activation by KCl prompts rapid functional responses that may require a fast activation of gene expression programs. This difference in the biological relevance between KCl activation and BDNF stimulation can be reflected in the distinct chromatin responses described here. At the chromatin accessibility level, we found that BDNF stimulation‐induced comprehensive changes in the enhancer landscape at an early stage, with concomitant gene expression changes. This regulatory network involving enhancer promoter interactions with the recruitment of different TFs to regulate transcription indicates a tight chromatin control. Conversely, KCl activation resulted in delayed chromatin remodeling of a similar set of enhancers. At an early timepoint, the KCl‐dependent changes in chromatin accessibility in promoters showed little correlation with transcription, albeit the transcriptional response was greater compared to BDNF. This could be related to the independence of cis‐chromatin elements in regulating gene transcription at an early timepoint of KCl or more complex TF dynamics at promoters involving TFs functioning as both activators and repressors (e.g., KLFs and E2Fs). Besides, neuronal activation by KCl depolarization also induces BDNF expression and its release to the postsynaptic cell, where the additional response to BDNF could confound the specific effect of KCl.

We speculate that the higher Fos protein levels in BDNF promote a more rapid opening of the distal regulatory regions, compared to KCl. Higher levels of Fos induction upon BDNF stimulation might be due to stronger activation of MAPK in BDNF compared to KCl, consistent with previous studies showing a correlation between MAPK activation and Fos expression (Whitmarsh, 2007). The variation in gene expression seen in BDNF and KCl likely arises because of the different levels and/or combinations of distinct co‐factor TFs bound with Fos in their newly accessible regulatory regions.

Fos, a classical pioneer factor assembling the AP‐1 complex (composed of Fos and Jun heterodimers, members of the bZIP protein family; Biddie *et al*, 2011), was a major driver increasing chromatin accessibility for both stimuli. We further revealed that multiple co‐regulatory TFs, such as ETSs, RFXs, and EGRs, are enriched with bZIP motifs and regulate gene expression, consistent with models that co‐regulators recruited in the vicinity of bZIP TFs may provide functional specificity (Su *et al*, 2017). The association with EGRs and their higher expression in BDNF over time suggests a major involvement in the chromatin and expression events induced by BDNF, consistent with EGR TFs regulating downstream target genes involved in synaptic plasticity and memory formation (Beckmann & Wilce, 1997; Gallitano‐Mendel *et al*, 2007). In addition, we found that the transcriptional repressor TF HIC1 (Pinte *et al*, 2004; Boulay *et al*, 2012; Ullah *et al*, 2018) is associated both with subsets of binding regions opened by BDNF and with the overall closing of early accessible regions, correlating with its strong early up‐regulation upon BDNF stimulation. The stronger co‐enrichment of HIC1 and EGR in BDNF‐gained peaks and weaker co‐enrichment of CTCF and EGR in KCl‐closing peaks (Fig 5A) indicates a further regulatory role through the interaction of these factors, as suggested previously (Pruunsild *et al*, 2017). Although a clear role for HIC1 motifs in BDNF‐induced gene expression needs to be resolved, HIC1 might act to regulate genes as an early repressor in regions opened by bZIP and rapidly co‐regulated by EGR factors. As co‐binding of TFs may target a more specific set of genes and thus lead to a more specific functional impact (Jolma *et al*, 2015; Vandel *et al*, 2019; Ibarra *et al*, 2020), a complex interplay of these factors in the onset of BDNF specific gene expression is possible and requires further investigation.

The relationship between chromatin accessibility and genomic regions associated with complex traits can provide a deeper understanding for diseases in the context of genetic variation at non‐coding regulatory regions (Maurano *et al*, 2012) that could affect TF occupancy (Vierstra *et al*, 2020). Previous studies showed that mapping mouse epigenomes with human conserved regions can reveal cis‐regulatory regions linked to human GWAS (McClymont *et al*, 2018; Hook & McCallion, 2020). Here, we found that neuronal regulatory regions conserved between mouse and human are enriched for genetic variants linked to human neuronal traits, and more predominantly in regions that increase chromatin accessibility upon BDNF, suggesting that BDNF‐activating chromatin associated with neuropsychiatric traits can be detected in several species. Limitations to our analysis lie in the inherent distinctions between *in vitro* and *in vivo* systems, represented by the difference in neuronal maturation states, interactions between different cellular types, and formation of neuronal networks, which can affect the chromatin response specific to BDNF. Therefore, chromatin events dependent on these parameters that only occur *in vivo* could not be captured in our setting. Nevertheless, associations with regions showing BDNF‐induced gains of expression in both species highlight the utility of comparative genomics in prioritizing and validating associations with complex traits, with our study describing a systematic approach for dissecting stimulation‐driven chromatin function in brain cells.

## Materials and Methods

### Reagents and Tools table

**Table:** 

	Reference or Source	Identifier or Catalog Number
**Experimental Models**		
Mice for dissection of embryonic cortical neurons	EMBL Animal House	CD1
Mouse embryonic stem cells	Laboratory of Kyung‐Min Noh	129XC57BL/6J generated from male 129‐B13 agouti mice
Human induced pluripotent stem cells	Michael Snyder lab, Stanford University	male H10‐CESCG‐297
**Recombinant DNA**		
pLV‐TetO‐hNGN2‐eGFP‐Puro	Addgene	Cat# 79823
FUdeltaGW‐rtTA	Addgene	Cat# 19780
pMD2.G	Addgene	Cat# 12259
psPAX2	Addgene	Cat# 12260
pspCas9(BB)‐2A‐GFP	Addgene	Cat# 48138
pspCas9(BB)‐2A‐RFP	Addgene	Cat# 91854
**Antibodies**		
Mouse‐anti‐Map2 mAb	Sigma	Cat# m9942
Chicken anti‐Synaptophysin pAb	Synaptic Systems	Cat# 101 006
Mouse‐anti‐TUBBIII mAb	Abcam	Cat# ab78078
Rabbit anti‐PSD95 pAb	Cell Signaling Technology	Cat# 3450
Camelid sdAB FluoTag‐X4 anti‐GFP labeled with ATTO488	Synaptic Systems	Cat# N0304‐At488‐L
Guinea pig anti‐MAP2 pAb	Synaptic Systems	Cat# 188 004
Guinea pig anti‐Synapsin1/2 pAb	Synaptic Systems	Cat# 106 004
Mouse anti‐Tau monoclonal AB	Novus	Cat# NBP2‐50051
Rabbit‐anti‐Egr1 mAb	Cell Signaling	Cat# 4153
Rabbit‐anti‐c‐Fos mAb	Cell Signaling	Cat# 2250
Goat‐anti‐rabbit IgG conjugated with AlexaFluor 594	Life Technologies	Cat# A11012
Goat‐anti‐mouse IgG conjugated with AlexaFluor 594	Life Technologies	Cat# A11005
Donkey anti‐chicken IgY (IgG) (H+L) conjugated with Alexa Fluor 488	Jackson ImmunoResearch Labs	Cat# 703‐545‐155
Cy3 donkey anti‐rabbit IgG (H+L)	Jackson ImmunoResearch Labs	Cat# 711‐165‐152
Donkey anti‐guinea pig IgG (H+L) conjugated with Alexa Fluor 647	Jackson ImmunoResearch Labs	Cat# 706‐605‐148
Goat anti‐mouse IgG (H+L) conjugated with Alexa Fluor 750	ThermoFisher	Cat# A‐21037
**Chemicals, enzymes and other reagents**		
Accutase	ThermoFisher	Cat# A1110501
Benzonase	Millipore	Cat# 71206‐3
Poly‐D‐Lysine	Sigma	Cat# P0899
Laminin	Sigma	Cat# 11243217001
Neurobasal medium	ThermoFisher	Cat# 21103049
Penicillin/streptomycin	ThermoFisher	Cat# 15140122
GlutaMAX	ThermoFisher	Cat# 35050
B27 supplement	ThermoFisher	Cat# 12587010
Leukaemia Inhibitory Factor (LIF)	EMBL Protein Expression and Purification Core Facility	
Retinoic acid	Sigma	Cat# R26250
N‐2 supplement	ThermoFisher	Cat# 17502048
DMEM	ThermoFisher	Cat# 11960085
Essential 8 medium	ThermoFisher	Cat# A1517001
Vitronectin	ThermoFisher	Cat# A14700
Neurobasal A	ThermoFisher	Cat# 10888022
DMEM/F12	Gibco	Cat# 51300‐044
Doxycycline	Sigma	Cat# D9891
Puromycin	Sigma	Cat# P8833
Fibronectin	Sigma	Cat# F0895
Neurobasal medium	ThermoFisher	Cat#21103049
L‐Ascorbic Acid	Sigma	Cat# A5960
DAPT	Tocris	Cat# 2634
CalPhos^TM^ Mammalian Transfection kit	Takara	Cat# 631312
Formaldehyde	ThermoFisher	Cat# 28906
Prolong Gold	ThermoFisher	Cat# P36934
D‐2‐amino‐5‐phosphonopentanoic acid (D‐AP5)	Tocris	Cat# 0106
Tetrodotoxin (TTX)	Tocris	Cat# 1078/1
Brain Derived Neurotrophic Factor (BDNF)	R&D systems	Cat# 248‐BDB
T‐5224	Cayman	Cat# 22904
Turbo^TM^ DNase	ThermoFisher	Cat# AM2238
MultiScribe^TM^ Reverse Transcriptase	ThermoFisher	Cat# 4311235
SYBR Green Master Mix	Applied Biosystems	Cat# 4309155
cOmplete protease inhibitor tablets	Roche	Cat# 11 873 580 001
Dynabeads protein G magnetic beads	ThermoFisher	Cat# 10003D
RNase A	ThermoFisher	Cat# EN0531
Proteinase K	ThermoFisher	Cat# EO0491
NEBNext Ultra II kit	New England Biolabs	Cat# E7645L
oligo‐dT capture kit	New England Biolabs	Cat# S1550
RNeasy kit	Qiagen	Cat# 74104
AMPure XP magnetic beads	Beckman Coulter	Cat# A63880
Sera‐Mag SpeevBeads	GE Healthcare	Cat# 45152105050250 and # 65152105050250
Filter plates	MSGVN22	Cat# MSGVN22
TMT10plex	ThermoFisher	Cat# 90110
TMT11	ThermoFisher	Cat# A37724
OASIS HLB plates	Waters	Cat# 186001828BA
Acclaim C18 PepMap 100	ThermoFisher	Cat# 164946
nanoEase M/Z HSS C18 T3	Waters	Cat# 186009249
**Other**		
Real‐Time PCR	Applied Biosystems	ABI7900
NextSeq 500 platform	Illumina	EMBL Heidelberg GeneCore Facility
Nextera DNA Library Prep Kit	Illumina	Cat # FC‐121‐1030
High Sensitivity DNA Bioanalysis Kit	Agilent	Cat# 5067‐4626
Qubit dsDNA HS Assay Kit	ThermoFisher	Cat# Q32851
NEBNext High‐Fidelity 2X PCR Master Mix	New England Biolabs	Cat# M0541S
Subcellular Protein Fractionation for Cultured Cells kit	ThermoFisher	Cat# 78840
Ultimate 3000 HPLC	Dionex	
Ultimate 3000 RSLC	ThermoFisher	
Q Exactive Plus Mass Spectrometer	ThermoFisher	
Nikon Ti‐2 widefield microscope	Nikon	

### Methods and Protocols

#### Primary cortical neuron culture

Mice husbandry and handling practices and procedures followed the routine and standard operating procedures. Prenatal embryos of CD‐1 mice at embryonic day 15 (E15) were used to isolate CNs with approval from the institutional animal care and use committee at EMBL. Pregnant female mice were euthanized with CO_2_ inhalation and consecutive cervical dislocation, and the embryos were decapitated to remove the brain. Embryonic cortex was isolated and dissociated by chopping with scalpel followed by digestion in accutase for 12 min at 37°C. During digestion, we treated the tissue with 250 unit/μl of benzonase to prevent neuronal clumping due to genomic DNA released from dead cells. Following digestion, neurons were triturated gently and passed through the 40 μm cell strainer before plating them onto a six‐well plate at a density of 1 × 10^6^ cells per well. Tissue culture plates were coated with 0.1 mg/ml of Poly‐D‐Lysine and 2.5 μg/ml of laminin. Primary neuronal cultures were maintained in Neurobasal medium containing 1% penicillin/streptomycin, 1% GlutaMAX, and 2% B27 supplement at 37°C with 5% carbon dioxide in the incubator. Post‐seeding after 1 day *in vitro* (DIV1), half of the media was replaced with fresh pre‐warmed Neurobasal media with all the supplements.

#### Neuronal differentiation of mESCs

Differentiation of mESCs to glutamatergic neurons was performed as previously described (Bibel *et al*, 2007) with minor modifications. Briefly, we removed the leukemia inhibitory factor (LIF) from mESC culture and grew cells in suspension using non‐adherent plates to enhance the formation of embryoid bodies. Every 2 days, differentiation medium without LIF was exchanged and on day 4, 5 μM of retinoic acid was added to the medium to promote the differentiation to the neuronal lineage. On day 8, neuronal precursor cells were dissociated using trypsin and plated at a density of 2 × 10^5^ cells/cm^2^ on plated coated with 0.1 mg/ml of Poly‐D‐Lysine and 2.5 μg/ml of laminin. Cells were cultured on N‐2 medium, consisting of regular DMEM supplemented with 1x N‐2 supplement, 1x B27 supplement, and antibiotics, which was replaced every 2 days. Experiments were always performed 4 days after plating cells.

#### Neuronal differentiation of hiPSCs

Human iPSCs (hiPSCs) derived from peripheral blood mononuclear cells with institutional review board approval (Stanford University, reference number 30064) were kindly provided by Michael Snyder's laboratory at Stanford University. hiPSCs were cultured in Essential 8 medium with supplement and vitronectin‐coated dish. To generate NGN2‐induced neurons (iNeurons), hiPSCs were transduced with TetO‐hNGN2‐P2A‐eGFP‐T2A‐PuroR and rtTA lentiviruses at 60% of confluency. After 1 day of transduction, the viral media was replaced with 1:1 ratio of Neurobasal A and DMEM/F12 medium containing GlutaMAX, insulin, N2/B27 without vitamin A supplements with 0.3% of glucose together with 1 μg/ml of doxycycline to induce rtTA expressions (Day 0). GFP‐positive hiPSCs were selected by adding 3 μg/ml puromycin (Day 2). After 2 days of puromycin selection, cells were dissociated with accutase and replated in triple coated dish with 0.1 mg/ml of Poly‐D‐Lysine, 10 μl/ml of Laminin, and 10 μl/ml of fibronectin in Neurobasal medium containing B‐27 w/o vitamin A, GlutaMAX, 200 μM of L‐ascorbic acid and 1 μM of DAPT (Day 4). 1 μg/μl of puromycin was added for 2 more days and 1 μg/μl doxycycline for 4 more days. Media was replaced every 2–3 days. After 10 days (Day14), iNeurons are stimulated with BDNF and KCl and subjected to ATAC‐seq.

#### Lentivirus generation

Two different lentiviruses containing human Neurogenin2 (hNGN2) under the control of TetON promoter or reverse tetracycline activator (rtTA) were generated. Each lentivirus was produced by co‐transfecting the transgene construct with two helper plasmids, pMD2 and psPAX2, into HEK293 cells using calcium phosphate transfection kit (Takara) following the manufacturer's protocol. After 48–72 h of incubation, media containing lentiviruses were harvested and concentrated at 28,000 *g* for 3 h, aliquoted, and stored at −80°C.

#### Immunofluorescent staining of human and mouse neurons

To evaluate the maturity of mouse primary CNs and NGN2‐EGFP‐induced iNeurons, cells were stained DIV10 or DIV13, respectively, using standard immunofluorescence staining protocols. In brief, cells were gently washed with 1xPBS, fixed for 15 min with 4% PFA (pre‐heated to 37°C), and subsequently permeabilized with 0.3% Triton X‐100/1xPBS for 3 min at room temperature (RT). Afterward, cells were washed thrice for 10 min at RT using washing buffer (1xPBS; 2% BSA; 25 mM glycine) and incubated for 1 h at RT with the following primary antibodies diluted in washing buffer: camelid sdAB FluoTag‐X4 anti‐GFP labeled with ATTO488 (1:500), mouse anti‐MAP2 monoclonal AB (1:1,000), guinea pig anti‐MAP2 polyclonal AB (1:1,000), rabbit anti‐PSD95 monoclonal AB (1:250), guinea pig anti‐Synapsin1/2 polyclonal AB (1:1,000), chicken anti‐Synaptophysin polyclonal AB (1:250), mouse anti‐Tau monoclonal AB (1:1,000), and mouse anti‐TUBBIII monoclonal AB (1:200). After washing three times 10 min RT with washing buffer, the primary antibodies were labeled with the following fluorophores (all 1:1000): Alexa Fluor 488 donkey anti‐chicken IgY (IgG) (H + L), Cy3 donkey anti‐rabbit IgG (H + L), Alexa Fluor 647 donkey anti‐guinea pig IgG (H + L), and Alexa Fluor 750 goat anti‐mouse IgG (H + L). After 3 washing steps with 1x PBS for 10 min at RT, cells were mounted with Mowiol (containing: 2.4 g Mowiol 4–88; 6 g glycerol, 6 ml H2O, and 12 ml 0.2 M TRIS/HCl (pH 8.5)).

Images were acquired as Z‐stacks of 10–15 planes at 500 nm steps size in 2 × 2 segments and stitched together based on a 15% overlap using a Nikon Ti‐2 widefield microscope controlled by NIS 5.2.02 software (Nikon). The microscope was equipped with a CFI P‐Apo DM 60x Lambda oil objective (Nikon), SPECTRA III light engine (Lumencor), and ORCA‐Fusion CMOS‐camera (Hamamatsu). Fluorescence was excited with 395 nm (DAPI), 475 nm (Alexa Fluor 488), 555 nm (Cy3), 635 nm (Alexa Fluor 647), and 748 nm (Alexa Fluor 750). Emission was filtered by a Pentafilter (432/515/595/681/809 nm) or by a 515/30 nm, 595/30 nm, and 682/42 nm filter for Alexa Fluor 488, Cy3, and Alexa Fluor 647, respectively. For representation, Z‐stacks were background and drift corrected and further processed as maximum projections using ImageJ software (version 2.3.0/1.53q) and plugins (Template Matching and Slice Alignment; Tseng *et al*, 2011).

#### Stimulation with BDNF and KCl, and T5224 treatment

Prior to every stimulation on DIV7, neurons were made quiescent for 2 h with 100 μM D‐2‐amino‐5‐phosphonopentanoic acid (D‐AP5) and 1 μM tetrodotoxin (TTX). KCl (55 mM) depolarization was performed by adding warmed KCl depolarization buffer (170 mM KCl, 2 mM CaCl_2_, 1 mM MgCl2and 10 mM 4‐(2‐hydroxyethyl)‐1‐piperazineethanesulfonic acid (HEPES)) to a final concentration of 31% directly into the neuronal culture medium and incubated for 1, 6, and 10 h. For the BDNF stimulation, neurons were incubated with BDNF (10 ng/ml) on DIV7 for 1, 6, and 10 h. Fos inhibitor T5224 was added 1 h prior to stimulation with BDNF from a 1,000‐fold concentrated stock in DMSO to the desired final concentrations.

#### Deletion of *Arc* putative enhancer with CRISPR‐Cas9 and determination of Arc expression by qPCR

To disrupt a putative EGR‐binding region of the Arc *gene* enhancer, two guide RNA sequences (Dataset EV5) were cloned into pspCas9(BB)‐2A‐GFP and pspCas9(BB)‐2A‐RFP, respectively, following the published protocol (Ran *et al*, 2013). Of each resulting plasmid, 2 μg were nucleofected into 2 × 10^6^ mESC using a Nucleofector (Lonza). After 48 h, samples were single‐cell sorted for GFP and RFP double positive‐cells. Colonies were expanded for genotyping and freezing. Deletion events were confirmed for homozygosity by agarose gel electrophoresis and checked by Sanger sequencing. CRISPR off‐target effects were tested with Sanger sequencing on predicted off‐target sites, and genome integrity was checked using low coverage genome sequencing.

A total of three mutant homozygous lines and three CRISPR control lines—resulting from CRISPR‐Cas9 editing rounds that did not include the desired deletion—were differentiated in duplicates to glutamatergic neurons and stimulated for 1 h with BDNF or KCl. T5224 treatment was conducted using two control and two KO lines, which were differentiated also in duplicates. *Arc*, *Fos*, and *Btg2* expression upon stimulation was quantified by RT‐qPCR. cDNA was generated from total RNA treated with DNase using MultiScribe™ Reverse Transcriptase. A total of 10 ng of cDNA were subjected to qPCR using PowerUp SYBR Green Master Mix and the StepOnePlus Real‐Time PCR system. Each reaction was assayed in triplicates (BDNF stimulation) or duplicates (KCl stimulation). Changes in *Arc* expression were assessed by normalization to *Rpl‐13* and unstimulated control (2^−ΔΔCt^). Primers used in RT‐qPCR measurements can be found in Dataset EV5.

#### Transcription factor ChIP‐qPCR in primary neurons

After stimulation, 4 million mouse primary neurons were fixed on plate with 1% formaldehyde for 10 min and quenched with 125 mM glycine. Cells were collected by scraping and snapfrozen. Chromatin for immunoprecipitation was prepared by lysing the cells on ice in sonication buffer (50 mM Tris–HCl pH 8.0, 0.5% SDS, protease inhibitors) and sheared yielding a fragment size distribution of 100–500 bp using Bioruptor Plus (12 cycles 30 s ON/30 s OFF). Soluble fraction was collected by centrifugation at 21,000 *g* and 4°C for 10 min. Chromatin was diluted six times in lysis buffer (10 mM Tris–HCl pH 8.0, 100 mM NaCl, 1% triton‐X 100, 0.5 mM EGTA, 1 mM EDTA, 0.1% Na‐Deoxycholate, 0.5% N‐lauroylsarcosine, protease inhibitors) and incubated overnight with Dynabeads protein G magnetic beads, which had been coated for 1 h at RT with 5 μl anti‐Fos or 5 μl anti‐Egr1 antibodies. Beads were washed six times, each time for 5 min with rotation; twice with RIPA buffer; twice with RIPA buffer supplemented with 300 mM NaCl; and twice with LiCl buffer (10 mM Tris–HCl pH 8.0, 250 mM LiCl, 0.5% NP‐40, 0.5% Na‐Deoxycholate, 1 mM EDTA). Beads were captured on magnet and rinsed quickly with TE buffer. To elute the immunocomplexes, 100 μl ChIP elution buffer (10 mM Tris–HCl pH 8.0, 300 mM NaCl, 5 mM EDTA, 0.5% SDS) were added and treated with 1 μl RNAse for 1 h at 37°C. Reverse cross‐linking was achieved by adding 1 μl proteinase K and incubating 1 h at 55°C followed by an overnight incubation at 65°C. DNA fragments were purified by phenol–chloroform extraction and ethanol precipitation. Library preparation was performed using NEBNext Ultra II kit with 50 ng of input material and all the immunoprecipitated DNA. Quantification by qPCR was done using 1 ng of DNA. Primers used can be found in Dataset EV5.

#### RNA‐seq sample preparation

Mouse CNs were collected at 1, 6, and 10 h after each stimulation for RNA isolation. The RNeasy kit (Qiagen) was used to extract RNA and genomic DNA was digested using the Turbo DNAse kit. To assess the quality of RNA all samples were analyzed using Bioanalyzer. Only samples with a RIN (RNA integrity number) score above 9 were used for library preparation. To prepare libraries we used the oligo‐dT capture kit in combination with the NEBNext Ultra II kit. We pooled 24 samples with each sample carrying a distinct barcode and sequenced on NextSeq 500 at EMBL, Heidelberg Gene Core facility.

#### RNA‐seq computational data processing

We mapped RNA‐seq reads in each sample to the *M. musculus* mm10 genome using TopHat2 (v2.1.1) (Kim *et al*, 2013), using the transcriptome defined in Gencode (version M16, Ensembl 91) as reference. We used mapped reads counts to call differentially expressed genes using DESeq2 (version 1.30.0) (Love *et al*, 2014), using the following setup as a model: *y* ∼ *stimulation*, where *y* are the normalized RNA gene expression levels and *stimulation* is either BDNF (1, 6, 10 h) KCl (1, 6, 10 h) or control (all samples). As PCA analyses indicate that control neurons cluster together regardless of timepoint, we processed raw counts for these samples using DESeq2, and compared gene expression levels of BDNF, KCl at any timepoint versus all control samples, estimating in each case log2 fold changes, standard errors, and significance using the Wald test implementation (two‐sided). 12,109 genes with adjusted *P*‐value < 0.1 were defined as DE‐genes. To study variability in gene expression using unsupervised clustering, we selected the top 5,000 DE‐genes, based on the lowest adjusted *P*‐value observed across all comparisons. 4,910 (out of 5,000 selected DE‐genes) of these genes have a coverage of 100 reads or more in at least one sample. We clustered the mean‐corrected expression changes in each gene using partitioning around medoids (PAM) clustering, setting the number of *k* medoids as 10. The number of clusters was decided based on the granularity described by significantly discovered GO terms (Fig 1D), and associations with DE‐gene trends (Fig 2F). We compared the enrichment of GO terms in each cluster versus other clusters using topGO (Alexa *et al*, 2006) version 2.42.0, using all genes belonging to other clusters as genome background.

#### ATAC‐seq sample preparation and sequencing

For ATAC‐seq, 50,000 mouse primary CNs and 100,000 human iNeurons were harvested by centrifugation at 500 *g* × 5 min at 4°C. Cell pellets were washed with an ice‐cold PBS buffer and 50 ml cold lysis buffer (10 mM Tris–HCl pH 7.4, 10 mM NaCl, 3 mM MgCl_2_, 0.1% NP‐40). The cell suspension was centrifuged at 500 *g* for 10 min at 4°C and supernatant was discarded. The nuclei‐enriched pellet was immediately used for transposition reaction using Nextera DNA Library Prep Kit. The transposition reaction was incubated at 37°C for 30 min and followed immediately by purification using QIAGEN MinElute Kit. Following steps were primarily based on the protocol described by Buenrostro *et al* (2013) with minor changes. Purified DNA was subjected to an initial step of PCR amplification consisting of five cycles using NEBNext High‐Fidelity 2X PCR Master Mix and standard barcoded primers of Nextera kit for each sample. We used 5 μl of partially amplified libraries from each sample to perform qPCR to determine the additional cycles for PCR amplification that were required while avoiding the saturation of the PCR, which may reduce the complexity of the original libraries. This was measured by comparing cycle numbers against 1/3 of the maximum fluorescence intensity using real‐time PCR graphs on ABI7900. After the additional PCR cycles, samples were purified using Agencourt AMPure XP magnetic beads. The quality of the DNA libraries was tested with the High Sensitivity DNA Bioanalysis Kit. DNA concentration of each library was measured with the Qubit dsDNA HS Assay Kit & fluorometer. Based on the concentrations of each library, eight samples were pooled together with each sample double barcoded for pair‐end sequencing on NextSeq 500 platform EMBL, Heidelberg Gene Core facility.

#### Chromatin accessibility computational data processing

We mapped ATAC‐seq reads in each sample to the *M. musculus* genome (build mm10) using bowtie2 (v2.3.4.1) (Langmead & Salzberg, 2012). Mapped reads in each sample were used to call peaks in each treatment and timepoint with MACS2 (v2.2.7.1) (Zhang *et al*, 2008), using the following parameters to call peaks in each paired‐end sample: “*‐‐nomodel ‐‐shift ‐75 ‐‐extsize 150*”. Then, we jointly analyzed called peaks and ATAC‐seq reads to call differentially accessible peaks using DiffBind [Stark R, Brown G (2011). DiffBind: differential binding analysis of ChIP‐Seq peak data]. We only considered peaks detected in at least two samples. We generated consensus peaks using an overlap of 66%. To correct counts per peak in all ATAC‐seq datasets we used a LOESS normalization step. Normalized ATAC‐seq peaks counts were obtained with DESeq2 using the following model: *y* ∼ *stimulation*, where stimulation is a treatment and time combination (e.g., BDNF_1h, KCl_6h). Similar to RNA‐seq analysis, based on PCA grouping of control samples we decided to pool all control samples and used them as a reference for comparison. Log2 fold changes between conditions and controls were assessed using Wald test (two‐sided), defining DA‐peaks when adjusted *P* < 0.1. Gained or closing DA‐peaks are based on positive or negative log2 fold changes versus the normalized means of control samples, respectively.

#### Chromatin accessibility, epigenomics, and gene ontology enrichments

General genomic annotations for gained and closing DA‐peaks in each treatment‐timepoint pair were done using HOMER (Heinz *et al*, 2010). To assess the enrichment of neuronal‐specific chromatin marks, we used a previously reported hidden markov model generated from multiple chromatin marks and ChIP‐seq data for neurons, using ChromHMM(Ernst & Kellis, 2012). As this model uses 15 states generated using the mouse genome build mm9, to interrogate our DA‐peaks we converted these annotation ranges to mm10 using liftOver with default parameters. We report the log2 fold enrichment between the number of nucleotides in one of the 15 states, versus the number of nucleotides overlapping other states, using the function OverlapEnrichment of ChromHMM. To assess GO enrichment of peaks proximal to DA‐peaks we used a binomial and hypergeometric tests as implemented in the GREAT server (McLean *et al*, 2010), with default parameters to map peaks to genes: Peaks are associated with genes if located upstream of a TSS up to 5,000 bp, downstream of a TSS up to 1,000 bp, or up to 1 Mbp away from a TSS. We used as background regions unchanged peaks and their proximal target genes.

#### Genomic data co‐variation and loop data analysis

We defined DREs as regions more than 2kbp and no further than 50Kbp from TSS regions. We assessed the enrichment of associations between DREs and DE‐genes by sign quadrants, counting associations between peaks and genes. When two peaks are linked to one gene, to avoid duplicate counts we considered the sign of the peak whose change had the lowest adjusted *P*‐value. Enrichment was done using 2 × 2 contingency tables between double‐positive and double‐negative quadrant counts, using Fisher's exact test.

To assess the enrichment in the number of observed DA‐peaks and DE‐genes linked to each other, we compared the observed associations versus the ones defined through a permutation approach where log2 fold changes are maintained and distance values are scrambled across DA‐peaks and DE‐genes. Let *P* and *G* be the number of DA‐peaks (*P*) or DE‐genes (*G*) observed for a particular stimulation/time condition. *P* and *G* are Poisson distributed, and for high mean values (> 100) their distribution can be approximated with a Normal distribution. Thus, we can assess the deviation from expectation by using an empirical permutation approach, in which we compare our observed counts against the mean and standard deviation estimated from P and G values from permuted data obtained by shuffling the gene/peak labels. Our permutation approach for DA‐genes and DA‐peaks only resamples features (genes/peaks) and maintains the distribution of observed log2 fold change and *P*‐values for each timepoint. This permuted distribution maintains time dependencies and is less biased than full numerical shuffling. We did 1,000 permutations to obtain the expected mean (mu) and standard deviation (s) values for each *P* and *G* distributions and used those to calculate Z‐scores for the observed values of *P* or *G* (x), that is, Z = (x − mu)/s. We defined an over‐representation of associations between DA‐peaks and DE‐genes when the Z‐scores for both peaks and genes were greater than 2.5. In addition to permutation statistics, results are assessed using 2x2 contingency tables and Fisher's exact test for both gene and peak counts. All results are corrected using BH's procedure (Dataset EV2).

Similar to DE‐genes, DA‐peaks were also assessed by clusters of variability using PAM clustering using *k* = 10. DA‐peak clusters were annotated by genomic location using HOMER (TSS or others). To assess the over‐representation of DE‐genes going up or down in each cluster, we calculated 2 × 2 contingency tables for closest DE‐genes up in each cluster versus down in other clusters, or down in cluster versus up in other clusters. We assessed the significance in both cases with Fisher's exact test, with BH correction.

ATAC‐seq peak pair correlations were generated using all of the peak‐pairs closer than 50 Kbp. ATAC peaks were stratified by none, one or both peaks annotated as DA‐peaks in at least one contrast versus control samples. To compare these significance‐stratified regions with Hi‐C contacts (Bonev *et al*, 2017), we allow a distance threshold of 10Kbp between Hi‐C peak anchors and each ATAC‐seq peak. Correlations were stratified by cell type using annotations by Bonev *et al*, based on shaman scores. This indicates higher covariation between peaks with known chromatin contact information.

Enrichment of same direction DA‐peaks and DE‐genes for Hi‐C regions was done as follows: For each distal DA‐peak, a Hi‐C score was calculated versus the TSS region of target genes. If the shaman score was positive, then the association was considered strong. Counts for strong versus non‐strong pairs were calculated for positive and negative peak‐gene associations, versus opposite sign directions, and assessed for significance using Fisher's exact test (Appendix Fig S4D). Multiple testing correction was done using Benjamini–Hochberg's procedure.

#### Motif enrichment analysis

We defined summit‐centered 200‐bp regions from all differentially accessible peaks as foreground regions and retrieved background regions for each one using GENRE (Mariani *et al*, 2017), using a custom mm10 background. Briefly, a representative background sequence set is generated from a mouse‐specific database of reference genomic regions, matching for equivalent GC‐content and CpG frequency, promoter overlap (extent of the sequence located within 2 kb upstream of a TSS), and repeat overlap. We mapped TF motifs used in foreground and background sequences using (i) an *8*‐mers reference set for 108 TF specificity groups generated from protein‐binding microarray (PBM) data (Mariani *et al*, 2017) and (ii) an extensive database of *PWM*s retrieved from the CIS‐BP database (Weirauch *et al*, 2013). For mapping of *8*‐mers, we used GENRE to define a single score per sequence as the best E‐score greater than 0.35, or −1 otherwise, using the PWMmatch function (R Biostrings) if the TF is represented as a PWM, and *grepl* if represented as a *k*‐mer. To scan PWMs from the CIS‐BP database, we used FIMO (Grant *et al*, 2011) with default parameters, and kept the highest score for downstream analyses. We used these scores to assess sensitivity and specificity using a ROC analysis in the classification of foreground versus background sequences as in each treatment and timepoint, respectively. We used the ROC‐AUC to define significantly enriched TF specificity modules and motifs. To assess significantly enriched modules, we used Wilcoxon rank sums tests, one‐sided, adjusted with a Benjamini–Hochberg procedure (FDR = 10%). If significant, we used a ROC‐AUC threshold of 0.55 for interpretation of enriched TFs. To show TF expression levels, we used both the TF family annotations provided by CIS‐BP and the TF specificity annotations from GENRE. One limitation of motif enrichment analyses based on k‐mers is the partial overlap of independent k‐mer groups due to shared k‐mers. In general, 8‐mers overlap between the 108 TF specificity groups is reduced, given the high value of k (8). Previous work to demonstrate this used PBM data and distinguished accurate separation between such datasets (Mariani *et al*, 2017). TF modules such as bZIP and NR2E require careful consideration, as they have a higher overlap of common 6‐ and 7‐mers.

#### TF motif co‐enrichments in DA‐peaks

The top five significant motifs with highest ROC‐AUC values observed were assessed for co‐enrichment in DA‐peaks. Fold Enrichment and significance of group‐wise over‐representations were assessed using the hypergeometric test, implemented in the package SuperExactTest (Wang *et al*, 2015).

#### Associations between bZIP and co‐regulatory TFs with accessibility and expression changes

We assessed the association of presence of bZIP and co‐factors motifs to changes in accessibilities and gene expression. Briefly, we compared log2 fold changes in the chromatin accessibility values for peaks with only bZIP motifs or bZIP and any co‐regulatory motif. This analysis was also repeated for the expression values of closest genes to mapped peaks, and for genes with mapped peaks harboring in TSS regions. Statistical differences were assessed using a Wilcoxon rank sums test, two‐sided, and adjusted using Benjamini–Hochberg's procedure.

#### CTCF‐specific analyses at differentially accessible peaks

To assess the enrichment of DA‐peaks for CTCF promoter‐exon loops we used a previously released dataset of promoter‐exon contact predictions (Ruiz‐Velasco *et al*, 2017) to compare the odds ratio between DA‐peaks in these loops versus unchanged peaks. We used Fisher's exact test to assess the overrepresentation of DA‐peaks in those regions, versus unchanged peaks in those regions. Additionally, we assessed the enrichment of convergent versus divergent CTCF motif orientations across all proximal ATAC peak‐pairs (less than 50Kbp) and regardless of promoter‐exon loops, using the CTCF PWM for this (CIS‐BP ID: M06483_1.94d) (Weirauch *et al*, 2013). Enrichment of DA‐peaks in exons and introns was done using 2 × 2 contingency tables by timepoint (1, 6, or 10 h) and DA‐peak label (gained, closing or unchanged). Adjusted *P*‐values in these analyses were corrected using Benjamini–Hochberg's procedure.

#### Differentially used exons computational and validation analyses

We called DUEs using mapped RNA counts across all samples in timepoint 1 h (Gencode version M16). We used genes with non‐zero counts in at least one treatment and timepoint to perform comparisons between BDNF and KCl in matched timepoints, using DEXSeq (Anders *et al*, 2012). *P‐*values are adjusted using Benjamini–Hochberg's procedure (FDR < 10%).

DUE for validation were based on the presence of a promoter‐exon CTCF loop predicted by Ruiz‐Velasco *et al* (2017). Primary CN cultures on DIV 7 were stimulated with BDNF or KCl for 1 h, each condition performed on independent biological duplicates, and RNA was isolated for expression analysis of the DUEs via RT‐qPCR. Primers for exons of three gene examples were selected, in addition to an additional same‐gene exon for internal control.

For Trio, exon 29 with additional 3′UTR (E29 + 3′UTR) sequence is a DUE between BDNF and KCl in timepoint 1 h. Three primer sets were designed to differentiate E29 (+3′UTR) from constitutive exon 28 (E28). In two of the sets, the forward primer lies within the coding region of E29 with a reverse primer in the immediately downstream 3′UTR region, whereas the third set did not include the 3′UTR region. For the constitutive exon, both forward and reverse primers lie in the coding region of E28. For Stxbp5 primer sets were designed to allow selective amplification of E01 against a constitutive exon, E05. For Cpe‐201 primer sets were designed, one for E06 which is a DUE and another for constitutive E09. All primer sequences used to perform qPCR can be found in Dataset EV5. The stimulation‐dependent fold change of each exon was quantified from 3 technical qPCR replicates normalized against Rpl13 as a reference gene. Fold changes relative to Rpl13 values were used to calculate the exon ratio between each tested exon and their internal exon as a reference. Comparisons between groups were assessed for statistical significance using *t*‐tests (two‐sided).

#### Subcellular proteome analysis by mass spectrometry

Stimulated mESC‐derived neurons were harvested using a scraper and pelleted at 500 *g* for 5 min. Cell pellets corresponding to 10 million cells were washed twice with ice‐cold PBS and subjected to subcellular protein extractions using a Subcellular Protein Fractionation for Cultured Cells kit following manufacturer's instructions. Each condition (Ctrl, BDNF, and KCl stimulations) were assayed in duplicates.

From each subcellular fraction, 10 µg of denatured protein, free of nucleic acids, were subjected to sample preparation for MS using a modified SP3 method (Hughes *et al*, 2014). Briefly, protein samples were precipitated onto Sera‐Mag SpeevBeads using filter plates for acidification and washes. Proteins were digested with trypsin and Lys‐C, and the resulting peptides were vacuum dried and labeled with TMT labels. For each replicate, labeled peptides from the following fractions were pooled to form a TMT11 set: unstimulated, BDNF‐stimulated and KCL‐stimulated, cytosolic (CEF), nuclear (NE), and chromatin‐bound (CHR) fractions (channels 1–9), as well as a membrane fraction (channel 10) and a cytoskeletal fraction (channel 11) pooled from all three different conditions to increase coverage. Pooled peptides were desalted, washed and vacuum dried on OASIS HLB plates. Before LC/MS analysis, peptides were pre‐fractionated into 12 fractions by Ultimate 3000 (Dionex) HPLC high‐pH reverse chromatography and vacuum dried. Reconstituted peptides were analyzed by nanoLC‐MS/MS on an Ultimate 3,000 RSLC connected to a Q Exactive Plus mass spectrometer. Peptides were loaded on a trapping cartridge (Acclaim C18 PepMap 100) using 0.1% FA (solvent A) and separated on an analytical column (nanoEase M/Z HSS C18 T3) with a constant flow of 0.3 μl/min applying a 120 min gradient of 2–40% of 0.1% FA in CAN (solvent B) in solvent A. Peptides were directly analyzed in positive ion mode. Full scan MS spectra with a mass range of 375–1,200 m/z were acquired in profile mode using a resolution of 70,000 (maximum fill time of 250 ms or a maximum of 3e6 ions (AGC)). Precursors were isolated using a Top10 method with an isolation window of 0.7 m/z, fragmented using 30 NCE (normalized collision energy), and MS/MS spectra were acquired in profile mode with a resolution of 35,000, and an AGC target of 2e5 with a dynamic exclusion window of 30 s.

#### Proteomic computational data analysis

Mass spectrometry raw files were processed using IsobarQuant (Franken *et al*, 2015) and peptide and protein identification was obtained with Mascot 2.5.1 (Matrix Science) using a reference mouse proteome (uniprot Proteome ID: UP000000589, downloaded 14.5.2016) modified to include known common contaminants and reversed protein sequences. Mascot search parameters were: trypsin; max. 2 missed cleavages; peptide tolerance 10 ppm; MS/MS tolerance 0.02 Da; fixed modifications: Carbamidomethyl (C), TMT16plex (K); variable modifications: Acetyl (Protein N‐term), Oxidation (M), TMT16plex (N‐term).

IsobarQuant output data were analyzed on a protein level in R (https://www.R‐project.org) using an in‐house data analysis pipeline. In brief, protein data were filtered to remove contaminants, proteins with less than two unique quantified peptide matches, as well as proteins, which were only detected in a single replicate. Subsequently, protein reporter signal sums were normalized within each subcellular fraction across the two TMT sets (replicates) and across the three conditions using the vsn package (Huber *et al*, 2002). Significantly changing proteins between BDNF and KCL stimulation conditions in the CHR subcellular fraction were identified by applying a limma analysis (Ritchie *et al*, 2015) on the vsn‐corrected signal sums. Replicate information was included as a covariate to adjust for batch effects caused by the separate TMT labelings and MS runs. *T*‐statistics and *P*‐values were obtained using the eBayes function from the limma package, and resulting *P*‐values were corrected for multiple testing using the Benjamini–Hochberg method with the topTable function in the limma package.

#### Linkage disequilibrium score regression analysis

For each group of Chromatin accessible regions, we performed partitioning heritability analyses to assess the proportion of variants associated with human complex traits harboring or proximal to differential accessible peaks. For mouse neurons, we used an adjusted *P*‐adjusted threshold of 0.2 for peak selection. In the case of hIPSC‐derived neurons, due to the low number of DA‐peaks detected we used raw *P*‐values for peak selection, and the same threshold. Annotations for mouse neurons and hIPSC‐derived neuron samples were extended to 10 Kbp, allowing recovery of local genetic variants in the context of peaks, and genetic signal of proximal linkage disequilibrium (LD)‐associated regions to be recovered.

We collected GWAS studies from two sources (i) a dataset of neuronal complex trait cohort pre‐processed using steps described by(Hook & McCallion, 2020), and (ii) neuronal and non‐neuronal complex traits GWAS cohort data preprocessed by Dr. Alkes Price group and available at https://alkesgroup.broadinstitute.org/sumstats_formatted. A description of the datasets and main features is provided in Dataset EV4.

Mouse regions were converted to human regions using *liftOver* and for all consensus peaks. Conserved blocks with length longer than 10,000 bp are discarded, recovering a total of 16,580 peaks mapped in the human genome.

Using as a reference genetic population data from 1000 Genomes Europeans (Auton *et al*, 2015), genetic variants in each study were pre‐processed to leverage LD‐block information. This modeling allows aggregation of LD‐genetic associated GWAS SNPs to be aggregated as tags and increases the strength of association between genomic annotations and polygenic trait datasets. Regression scores for gained DA‐peaks, closing DA‐peaks, all DA‐peaks (DA) and consensus chromatin peaks are independently interrogated against all GWAS studies by computing partitioned heritability coefficients, using the package *ldsc* (Finucane *et al*, 2015). Significance *P*‐values associated with the estimate of the first regression coefficient are adjusted for multiple testing correction using Benjamini–Hochberg's procedure.

## Author contributions

**Kyung Min Noh:** Conceptualization; supervision; funding acquisition; investigation; writing – original draft; project administration; writing – review and editing. **Ignacio L Ibarra:** Data curation; formal analysis; investigation; visualization; methodology; writing – original draft; writing – review and editing. **Vikram S Ratnu:** Conceptualization; formal analysis; validation; visualization; writing – original draft. **Lucia Gordillo:** Formal analysis; validation; visualization; writing – original draft; writing – review and editing. **In‐Young Hwang:** Data curation; methodology. **Luca Mariani:** Resources; methodology. **Kathryn Weinand:** Resources; methodology. **Henrik Hammarén:** Resources; formal analysis; visualization; methodology. **Jennifer Heck:** Formal analysis; visualization; methodology. **Martha L Bulyk:** Resources; supervision; methodology. **Mikhail M Savitski:** Resources; supervision; investigation; methodology. **Judith B Zaugg:** Supervision; funding acquisition; investigation; methodology; writing – original draft; project administration; writing – review and editing.

In addition to the CRediT author contributions listed above, the contributions in detail are:

VSR and K‐MN conceived the study. JBZ and K‐MN jointly supervised the computational parts of the study. VSR performed mouse primary neurons sample preparations, ATAC‐seq, RNA‐seq, CRISPR, and exon splicing studies under the supervision of K‐MN, ILI conducted all pre‐ and post‐processing computational analyses, under the supervision of JBZ, LG conducted Western blots, proteomic sample preparation and data interpretation from mESC‐derived neurons, RT‐qPCR measurement and interpretation for enhancer CRISPR knockouts, treatment with Fos inhibitor of CRISPR knockouts with statistical analysis and Egr1/Fos ChIP‐qPCR experiments, under the supervision of K‐MN, LM, KW, and MLB prepared a database of mouse chromatin negative regions for motif enrichment and helped in the interpretation of 8‐mer enrichment results. IH and JH helped with data acquisition. HMH conducted proteomics measurements and post‐processing analyses under the supervision of MMS, ILI and VS wrote the original manuscript with input from K‐MN and JBZ, ILI, K‐MN, and JBZ wrote the manuscript, with the input and approval of all co‐authors.

## Disclosure and competing interests statement

The authors declare that they have no conflict of interest.

## Supporting information



Appendix

Dataset EV1

Dataset EV2

Dataset EV3

Dataset EV4

Dataset EV5

## Data Availability

Processed data and scripts of relevant computational analyses are available at https://git.embl.de/rio/neuronal_activity_bdnf. RNA‐seq and ATAC‐seq raw data for mouse, including normalized counts and processed genes and peaks comparison have been deposited in Gene Expression Omnibus, with accession code GSE166959 (http://www.ncbi.nlm.nih.gov/geo/query/acc.cgi?acc=GSE166959). Human ATAC raw reads are deposited in the EMBL‐EBI European Genome‐Phenome Archive (EGA, ID: EGAS00001006394; https://ega‐archive.org/studies/EGAS00001006394). The mass spectrometry proteomics data have been deposited to the ProteomeXchange Consortium via the PRIDE (https://pubmed.ncbi.nlm.nih.gov/30395289/) partner repository with the dataset identifier PXD022378 (http://www.ebi.ac.uk/pride/archive/projects/PXD022378).
