# Supplementary material for: Comparative chromatin accessibility upon BDNF stimulation delineates neuronal regulatory elements
Source: Mol Syst Biol. 2022 Aug 23;18(8):e10473. doi: 10.15252/msb.202110473 (PMC9396287; doi:10.15252/msb.202110473)

APPENDIX: Table of Contents

Appendix Figure S1 RNA-seq computational data processing

Appendix Figure S2 ATAC-seq computational data processing

Appendix Figure S3 Effect of FDR 1% in DE-genes, DA-peaks, and downstream analyses.

Appendix Figure S4 Chromatin peaks variability and association with High-conformation Capture data (Hi-C).

Appendix Figure S5 Association between DA-peaks and genomic and RNA features

Appendix Figure S6 Enrichment of TF motifs in BDNF and KCl differentially accessible ATAC-seq peaks.

Appendix Figure S7 Dynamics of bZIP motif-binding Fos protein.

Appendix Figure S8 Effect of bZIP and co-regulatory TFs on BDNF and KCl at 6 hours post-stimulation, and genome tracks for modified Arc distal regulatory element perturbed using CRISPR in mESC-derived neurons.

Appendix Figure S9 Validation of Fos and Egr1 binding to Arc enhancer.

Appendix Figure S10 Association between promoter-exon CTCF-loops, chromatin DA-peaks, and differential usage of exons.

Appendix Figure S11 Disease Ontology associations for mouse DA-peaks and Partitioning heritability analysis of mouse primary cortical neurons and hiPSC-derived neurons.

Appendix Figure S12 Histological staining of mouse and hIPSC-induced neurons and differentially accessible peaks upon treatment with BDNF and KCl

Appendix Figure S1 RNA-seq computational data processing

(**a**) qPCR measurement of immediate early gene expression levels after 15, 30 and 60 minutes of stimulation with BDNF or KCl, normalized to RPL-13 (housekeeping gene) and unstimulated neurons (0min) (n=2 biological replicates, error bars as s.d.)

(**b**) Principal component analysis visualization of RNA-seq samples based on rld-normalized data, using PC pairs between PC1 and PC4 (treatment = colors; time=shapes).

(**c**) Gene expression Log2 fold change correlations between genes from BDNF- and KCl-stimulated neurons (y-axis), versus DE-genes reported in mouse cultured neurons stimulated with KCl (x-axis)(Ataman *et al*, 2016).

(**d**) (*left*) Hierarchical clustering of all RNA-seq samples, using the union of DE-genes found in any comparison versus control. (*right*) Hierarchical clustering of all RNA-seq samples, using all genes and normalized counts.

Appendix Figure S1

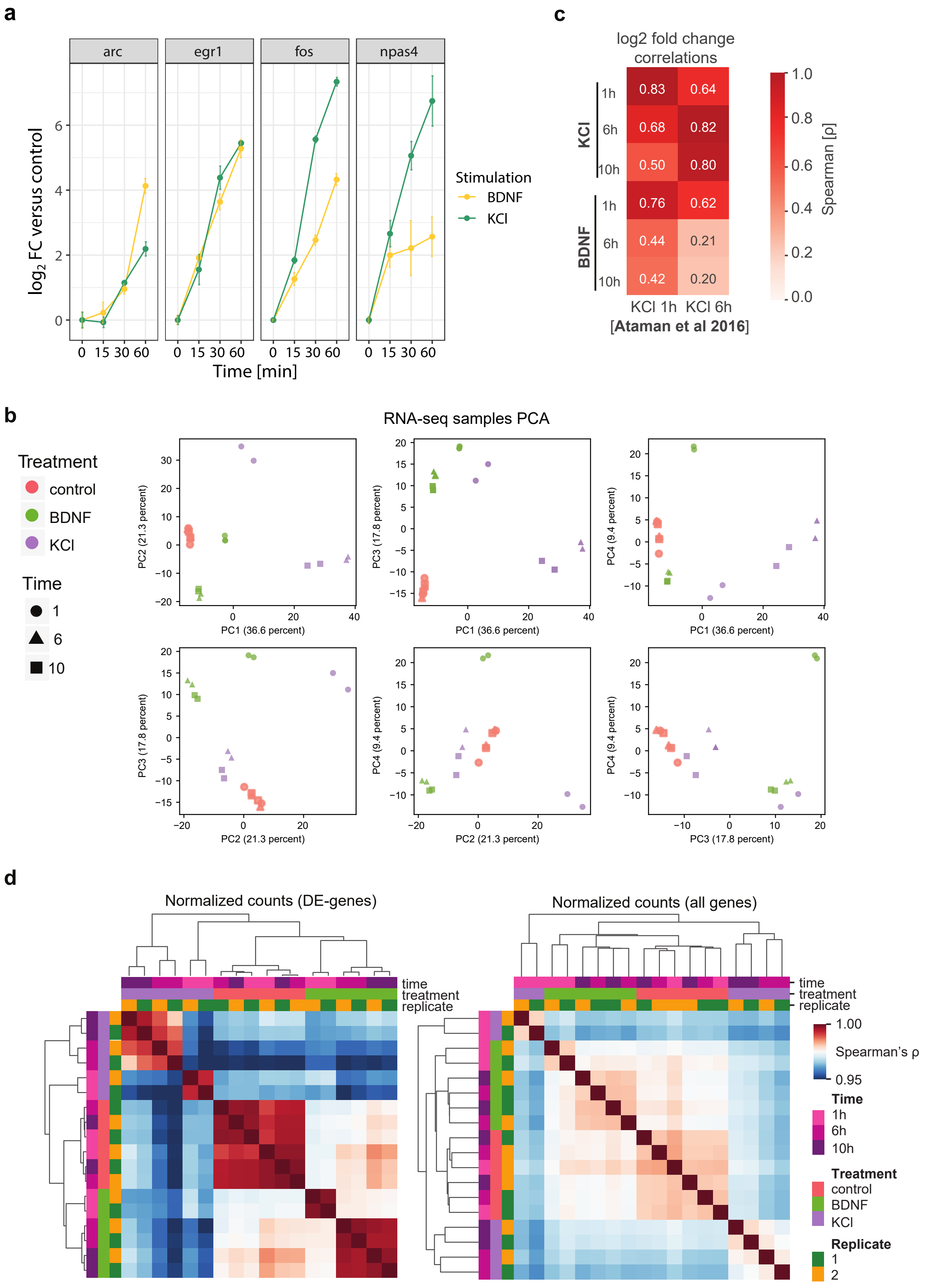

Appendix Figure S2 ATAC-seq computational data processing

(**a**) Principal component analysis visualization for treatment and time points using normalized counts generated with DiffBind [**Stark and Brown, 2011. Bioconductor**].

(**b**) MA-plots for consensus peaks. Differentially accessible peaks (DA-peaks; red dots) obtained in ATAC-seq samples for KCl 1h (left) and BDNF 1h (right). Red points indicate a peak with accessibility levels higher (log fold change > 0) or lower (log fold change < 0) compared to matched controls (FDR=10%).

(**c**) Percentage distribution of DA-peaks for BDNF and KCl 1h in peak annotations (using HOMER). Asterisks indicate within-stimulation Fisher's exact test comparisons, if annotation fraction are statistically significantly different between gained and closing DA-peaks (*adjusted P <0.05; **P < 0.01; ***P < 0.001).

(**d**) Chromatin states enrichments in DA-peaks for neuronal marks, based on ChromHMM 15-states model using neuronal epigenomics datasets (**Methods**). N indicates the total number of DA-peaks associated with each annotation. Black heat map indicates emission probabilities. Green/purple heat maps indicate fold enrichment between nucleotides coverage for DA-peaks in those annotations and background. Black-contoured squares indicate a change with the highest log2 fold change in each stimulation. Barplots summarize the highest log2 fold enrichment value for BDNF (yellow) and KCl (green). Fold enrichments with the greatest change between BDNF and KCl highest values are labeled.

Appendix Figure 2

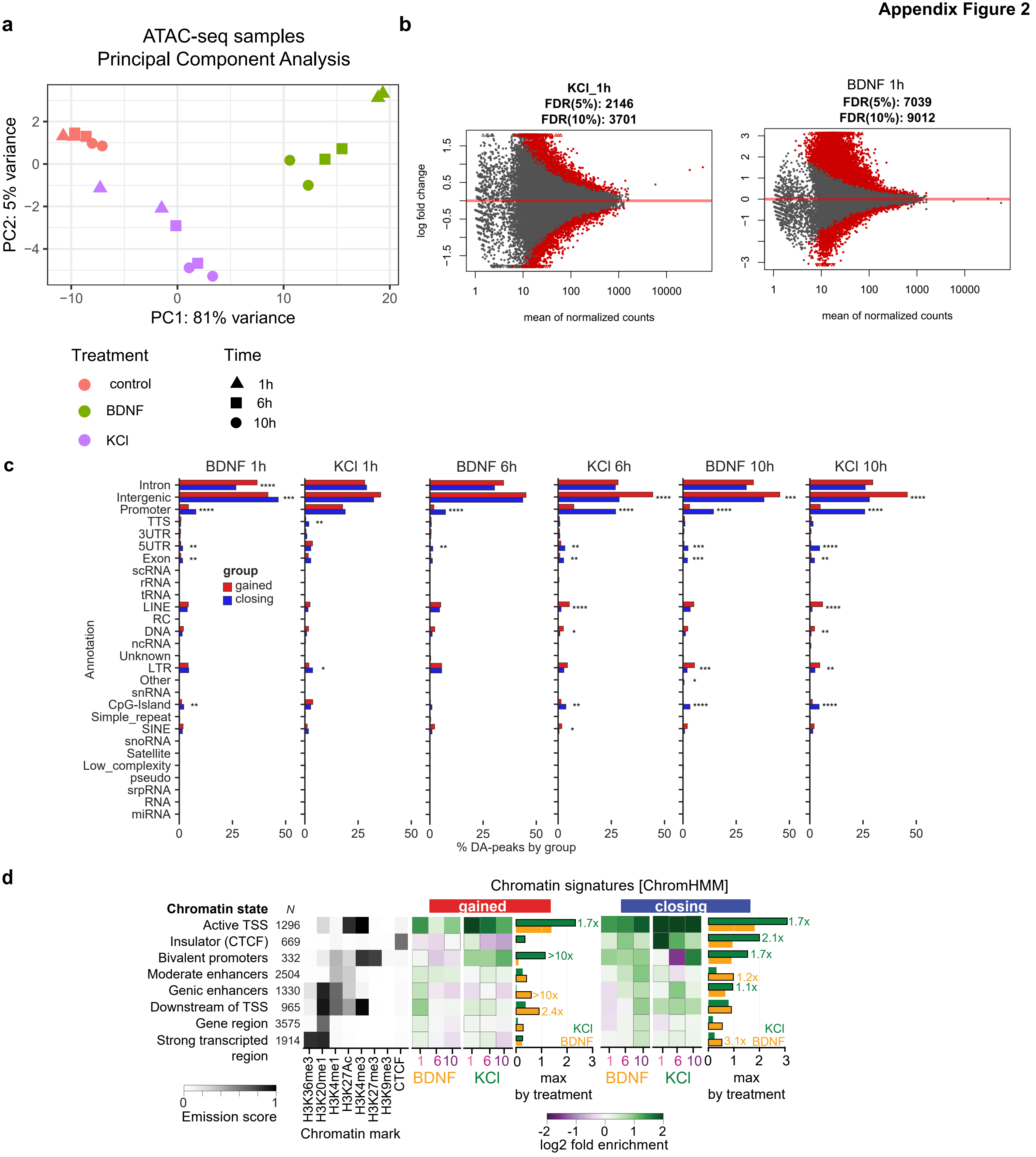

Appendix Figure S3 Effect of FDR 1% in DE-genes, DA-peaks and downstream analyses.

(**a**) number of DA-genes using FDR = 1%. Labels and colors as in **Figure 1c**.

(**b**) number of DA-peaks using FDR = 1%. Labels and colors as in **Figure 2b**.

(**c**) GO analysis using subset of 4,907 genes with FDR = 1% and more than 100 counts. Colors and legends as indicated in **Figure 1e**.

(**d**) TF motif enrichment analyses using DA-peaks with FDR=1% and GENRE-negative regions for those. Colors, legends as in **Figure 3b**.

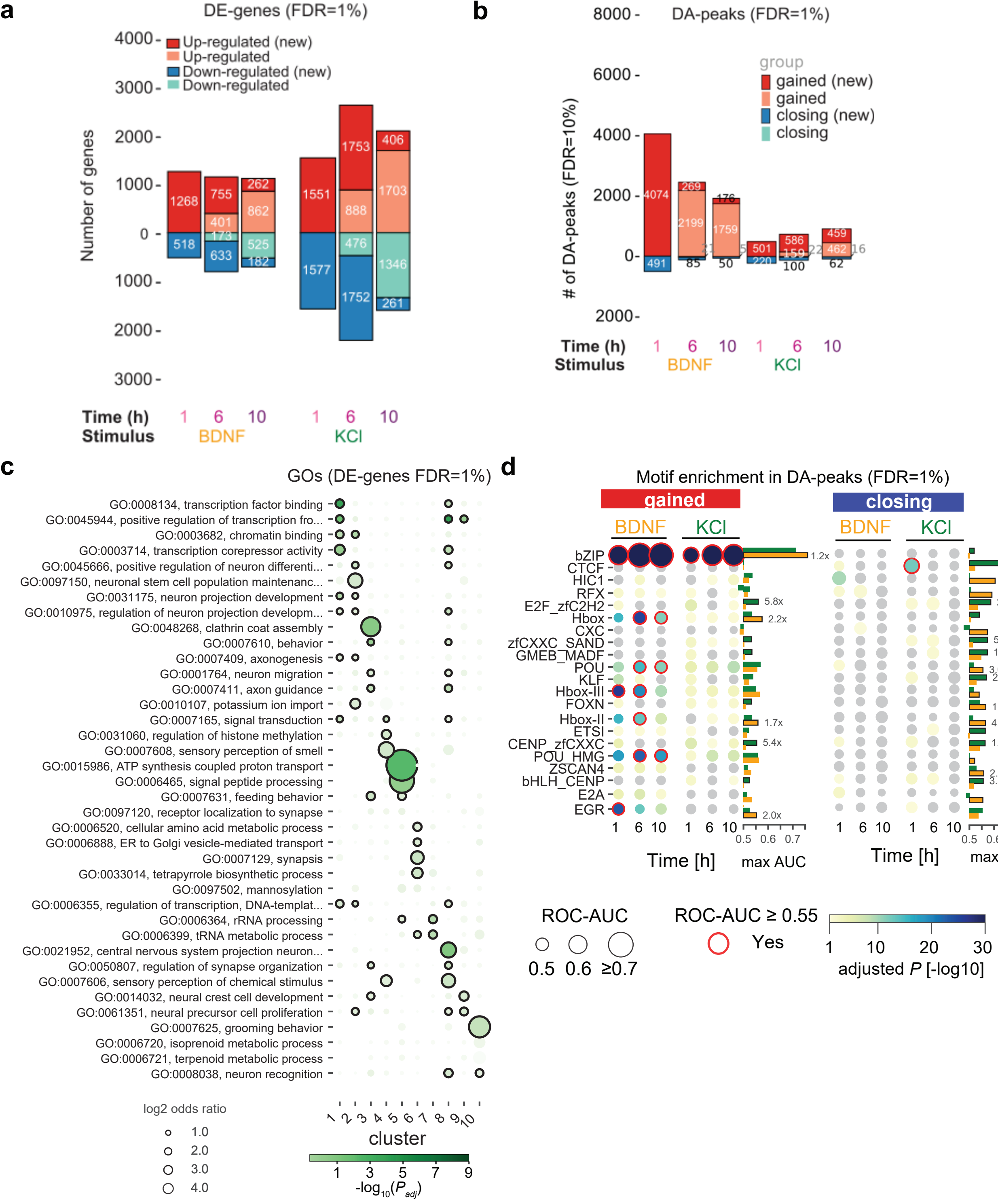

Appendix Figure 3

Appendix Figure S4 Chromatin peaks variability and association with High-conformation Capture data (Hi-C).

(**a**) Correlation distributions for normalized counts of proximal peak pairs with zero, one or two DA-peaks in that pair, grouped by Hi-C data evidence (CN=cortical neurons; NPC=neural progenitor cells; ES=Embryonic stem cells). Hi-C data from(Bonev *et al*, 2017).

(**b**) Enrichment of same sign log2 fold changes for pairs of DA-peaks and proximal DE-genes when grouped as positive Hi-C loops (strong Hi-C contacts), versus different signs in the same group. Asterisk indicates P < 0.1 significant enrichment based on Fisher's exact test (BH adjusted).

(**c**) Heatmap indicating the number of events where a DE-gene is included in a loop in cortical neurons (CN), neural progenitor cells (NPC), or embryonic stem cells (ES), for any of the DE-genes comparisons between BDNF and KCl versus control cells (purple). Right log2 odds ratio indicate the relationship (DE:loop / non-DE-loop) / (non-DE:loop / non-DE:non-loop)

(**d**) Similar to **c,** now indicating the number of DA peaks with loops in any of the described categories. Log2 odd ratios indicate the relationship (DA-peak:loop / non-DA-peak:loop) / (non-DA-peak:loop / non-DA-peak:non-loop)

(**e**) Similar to **c-d,** indicating the number of genes connected to peaks as DE-gene/DA-peak pairs, in any of the described categories. Log2 odd ratios indicate the relationship (DA+DE:loop / DE:loop) / (non-DA-peak:loop / non-DA-peak:non-loop)

(**f**) Top gene ontology results for a query of 1,449 DE-genes with loops (foreground) and 3,859 non-DE genes with loops (background) associated with BDNF 1h and CN loops. Colors indicate raw *P*-values.

(**g**) Top gene ontology results for a query of 446 DE-gene/DA-peak pairs with loops (foreground) and 1,752 DE-genes with loops (background) genes associated with BDNF 1h and CN loops.

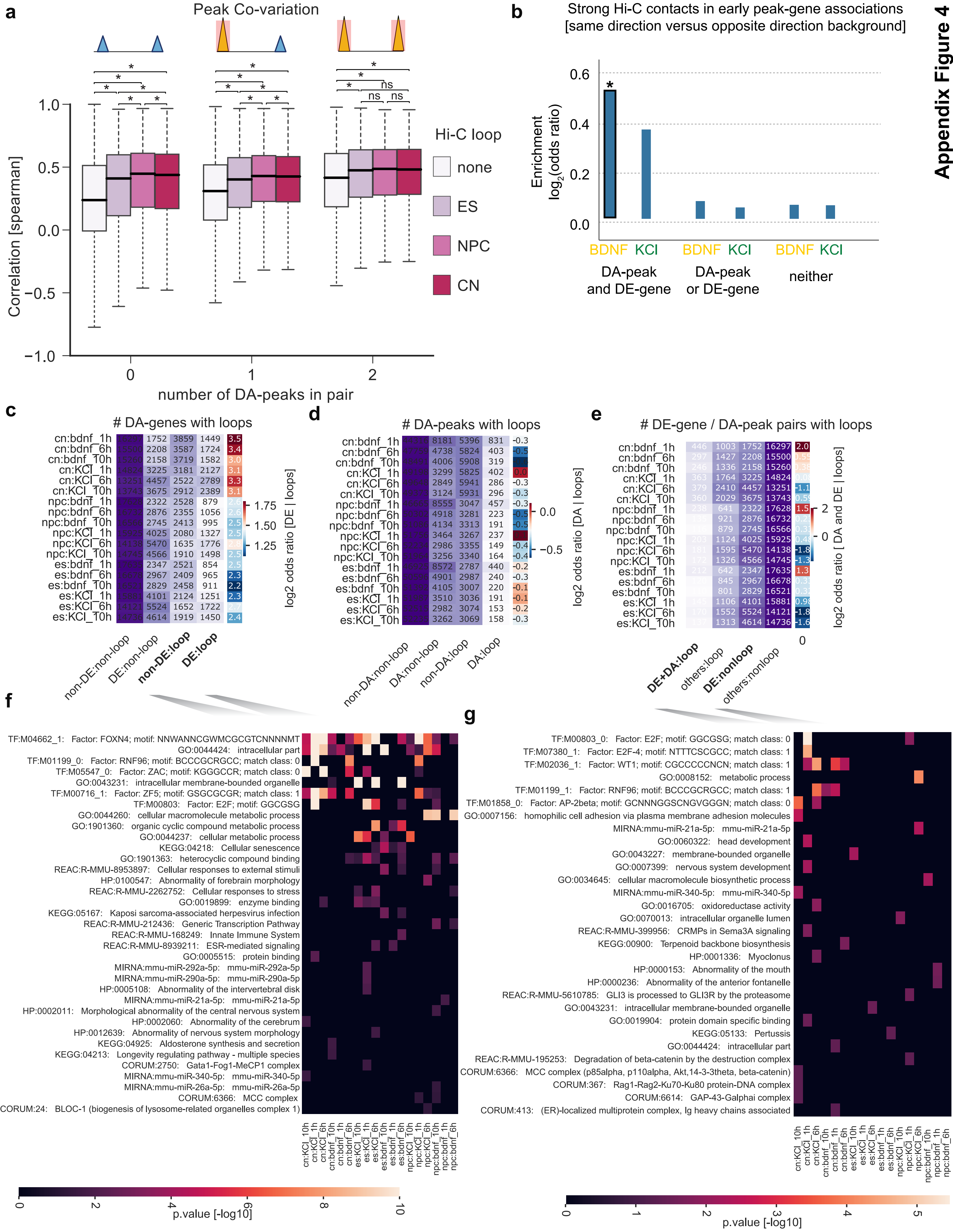

Appendix Figure 4

Appendix Figure S5 Association between DA-peaks and genomic and RNA features

(**a**) Cumulative distribution function of expression changes (log2) between genes based on the presence of DA-peaks (gained or closing), or unchanged (gray line).

(**b**) (*top*) Association between peaks at transcription start sites (TSSs) and gene expression at BDNF 1h. Each point indicates the log2 fold change of an ATAC-seq peak and the gene expression of a closest gene with distance between 0-2Kbp from TSS regions. Colors indicate whether none (gray), only the peak (orange), or both peak and gene (red) show significant changes versus control. (*bottom*) Enrichment for paired DA-peak and DE-gene in the four quadrants are summarized for BDNF and KCl. Asterisks indicate *P* values as corrected by a Benjamini Hochberg procedure (* = $P < 0.05$; ** = $P < 0.01$).

(**c**) Number of associations found between DE-genes comparisons (x-axis) and DA-peaks comparisons (y-axis) based on same (up/gained; down/closing) or opposite directions. Colors indicate significance Z-scores based on observed versus expected values from permutations.

Appendix Figure 5

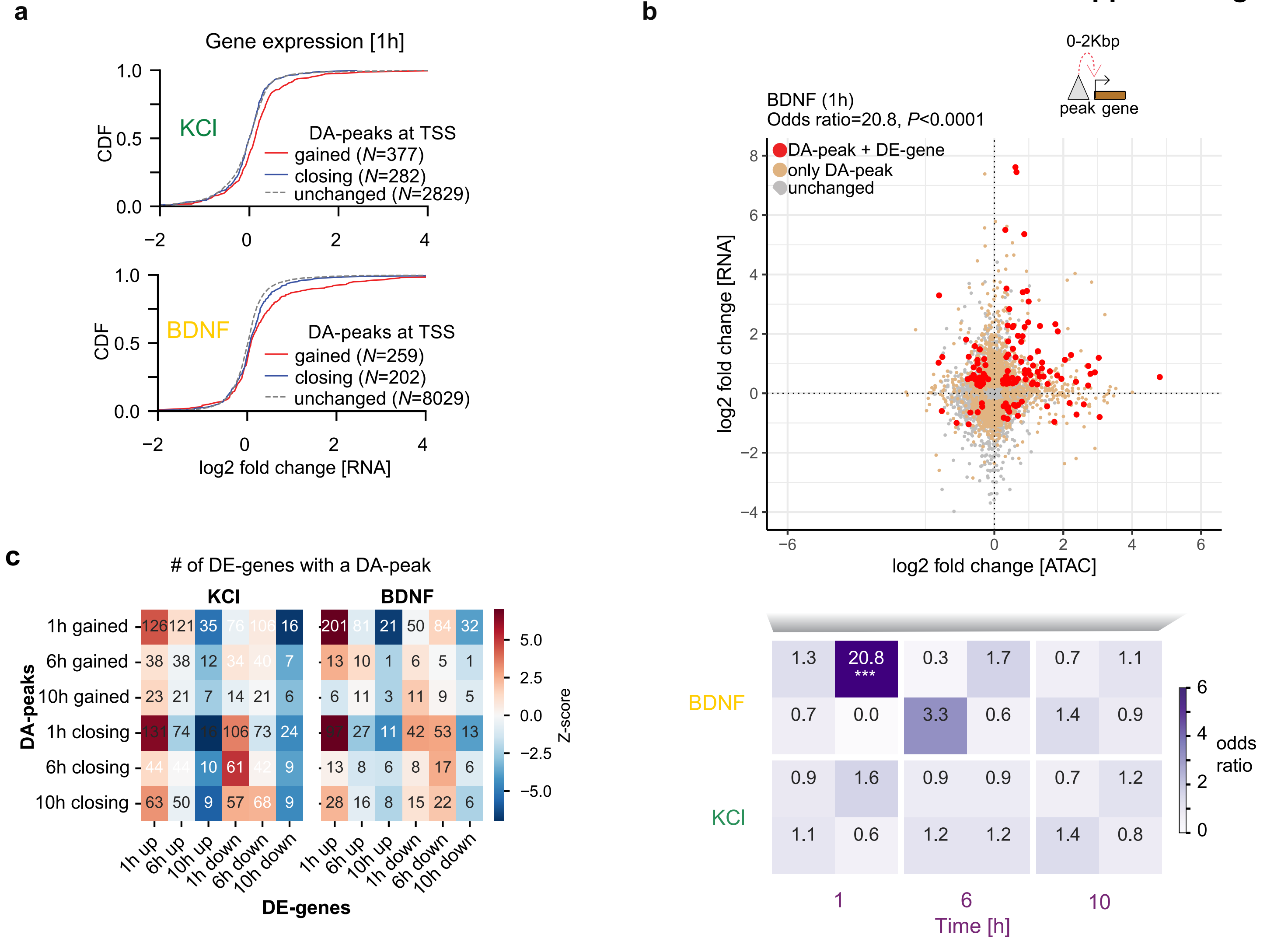

Appendix Figure S6 Enrichment of TF motifs in BDNF and KCl differentially accessible ATAC-seq peaks.

(**a**) Total fraction (as percentage) of DA-peaks explained by enriched TF motifs (minimum ROC-AUC > 0.55).

(**b**) *8*-mers modules harboring gained DA-peaks are visualized by their overall enrichment (ROC-AUC, *y*-axis) versus median relative distance to the peak summit for all motif observations (*x*-axis). Circle sizes indicate adjusted *P*-value significance based on a comparison against negative regions (rank sums test, with BH correction). bZIP has the highest enrichment and additionally the lowest median distance to the summit for BDNF 1 and 6h.

(**c**) Enrichment of bZIP-associated motifs in gained DA-peaks stratified by genomic annotation (HOMER). The number in each cell indicates the number of motifs. Asterisks indicate significance, (adjusted P < 0.01 based on Wilcoxon tests, with BH correction).

Appendix Figure 6

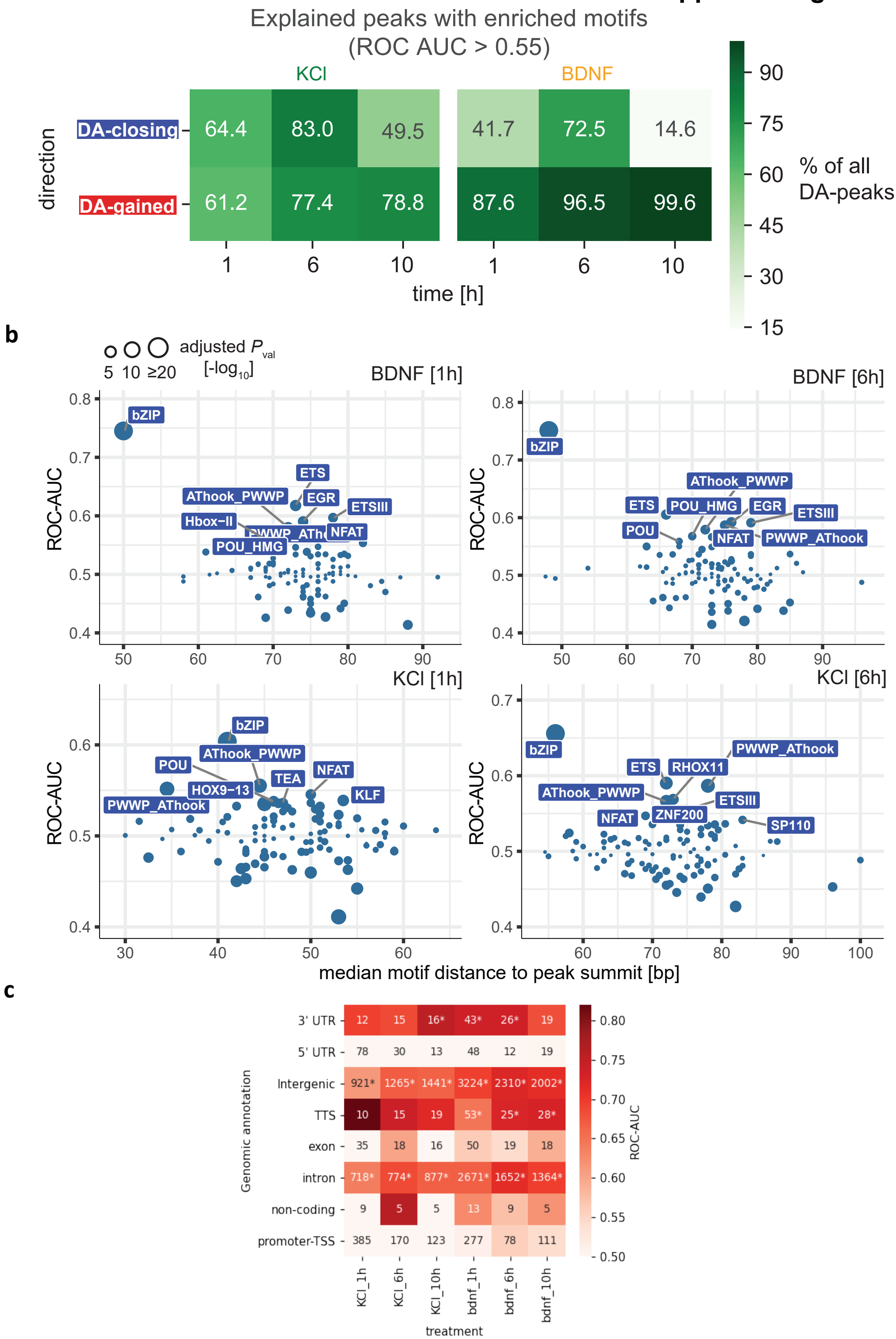

Appendix Figure S7 Dynamics of bZIP motif-binding Fos protein.

(**a**) Analysis of Fos protein levels after 1h and 6h of treatment by western blot, normalized to nucleolin levels. Quantification was performed on the 1h stimulation samples with 5 independent replicates (***P* <0.01, **P*<0.05, ns: not significant, two-sided t-test, error bars as s.d.)
(**b**) Principal component analysis visualization of batch-corrected, variance stabilized transformed values of proteins identified by mass spectrometry on chromatin after treatments (*n*=2 biological replicates)
(**c**) Linear correlation between RNA (y-label) and proteomics (x-label) overall changes versus control samples, shown as the linear correlation between readouts for both TF-related genes ('TF') and non-TFs ("Other").
(**d**) BDNF protein abundance change readouts for 187 matched TFs (log2 fold changes versus control) versus RNA (log2 fold changes BDNF 1h versus control). The title indicates linear correlation value and significance. Dot outlier in the top-right indicates the gene Fos. Labels indicate the top 15 TFs, sorted descendingly by the maximum value in either x- or y-labels.

Appendix Figure 7

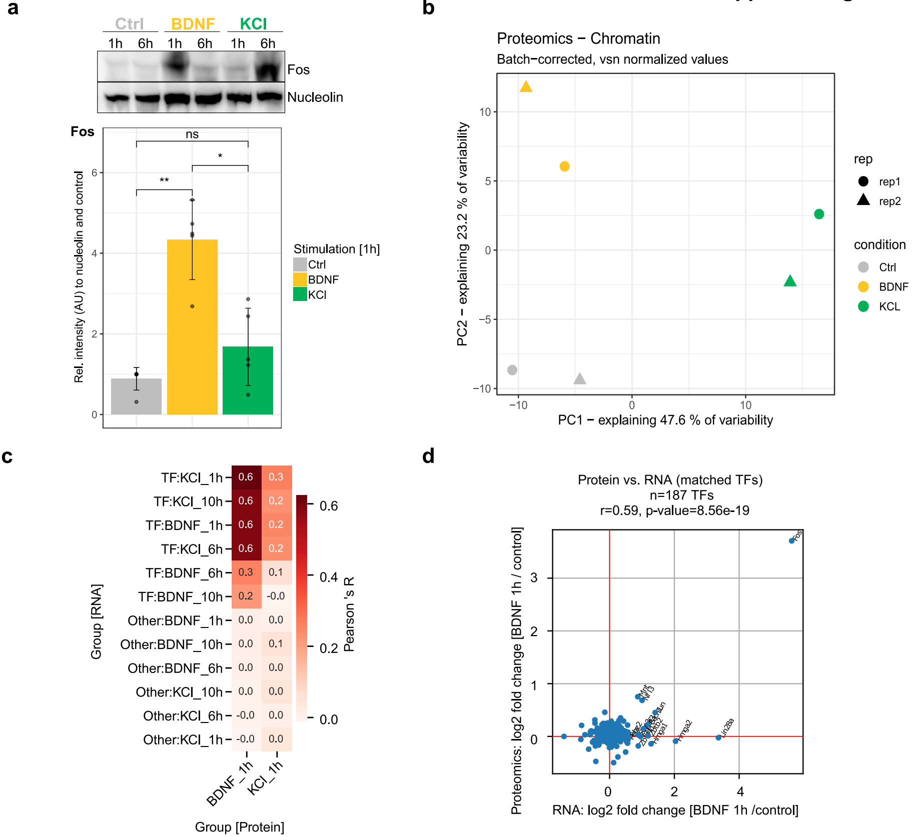

Appendix Figure S8 Effect of bZIP and co-regulatory TFs on BDNF and KCl at 6 hours post stimulation, and genome tracks for modified Arc distal regulatory element perturbed using CRISPR in mESC-derived neurons.

(**a**) Changes in log2 fold change between bZIP containing peaks and bZIP+TF2 containing ATAC-seq peaks in time point 6h. Legends as in **Figure 4a-b**

(**b**) (*top*) Genome track for Arc gene and distal enhancer. Highlighted mutated enhancer region in gray box. Coordinate indicates consensus peak region using DiffBind. Numbers in parentheses indicate the absolute number of motifs detected for each highlighted TF specificity module.

(*bottom*) aligned nucleotide sequences for one control replicate (A5) and three homozygous replicates (B1, C5, E11). aligned in the mouse genome (mm10). Red region indicates the deleted region based on sequencing data. Motifs for ERG, bZIP, HIC1 and Hbox are highlighted whenever a region with 8-mer hits with a high score is detected.

(**c**) Arc expression values after 1h of BDNF treatment compared to unstimulated control (and housekeeping gene RPL-13) measured by qPCR in the different CRISPR control (grey box) and Arc enhancer KO lines (blue box). Each line (3 control lines-A8, R1, R8 and 3 KO lines-B1, C5, E11, defined by the dot shape) was differentiated to neurons and stimulated in biological duplicates (n=3 technical qPCR measurements). Central band in indicates the median, boxes depict third and first quartiles and whiskers show the 1.5× IQR above and below the box. Two-sided *t*-test (***P*<0.01).

(**d**) qPCR measurement of Arc, Btg2 and Fos expression in control (grey) and Arc enhancer KO lines (blue) after 1h of BDNF stimulation in the presence of varying concentrations of T5224. Normalized to RPL-13 (housekeeping gene) and unstimulated cells without the inhibitor (biological duplicates of 2 control and 2 KO lines (B1, E11), measured in triplicates). Boxplot: central band indicates the median, boxes depict third and first quartiles and whiskers show the 1.5× IQR above and below the box). Two-sided t-test (*, **, ***, **** = $P < 0.05$, 0.01, 0.001 and 0.0001, respectively, and ns: not significant).

Appendix Figure 8

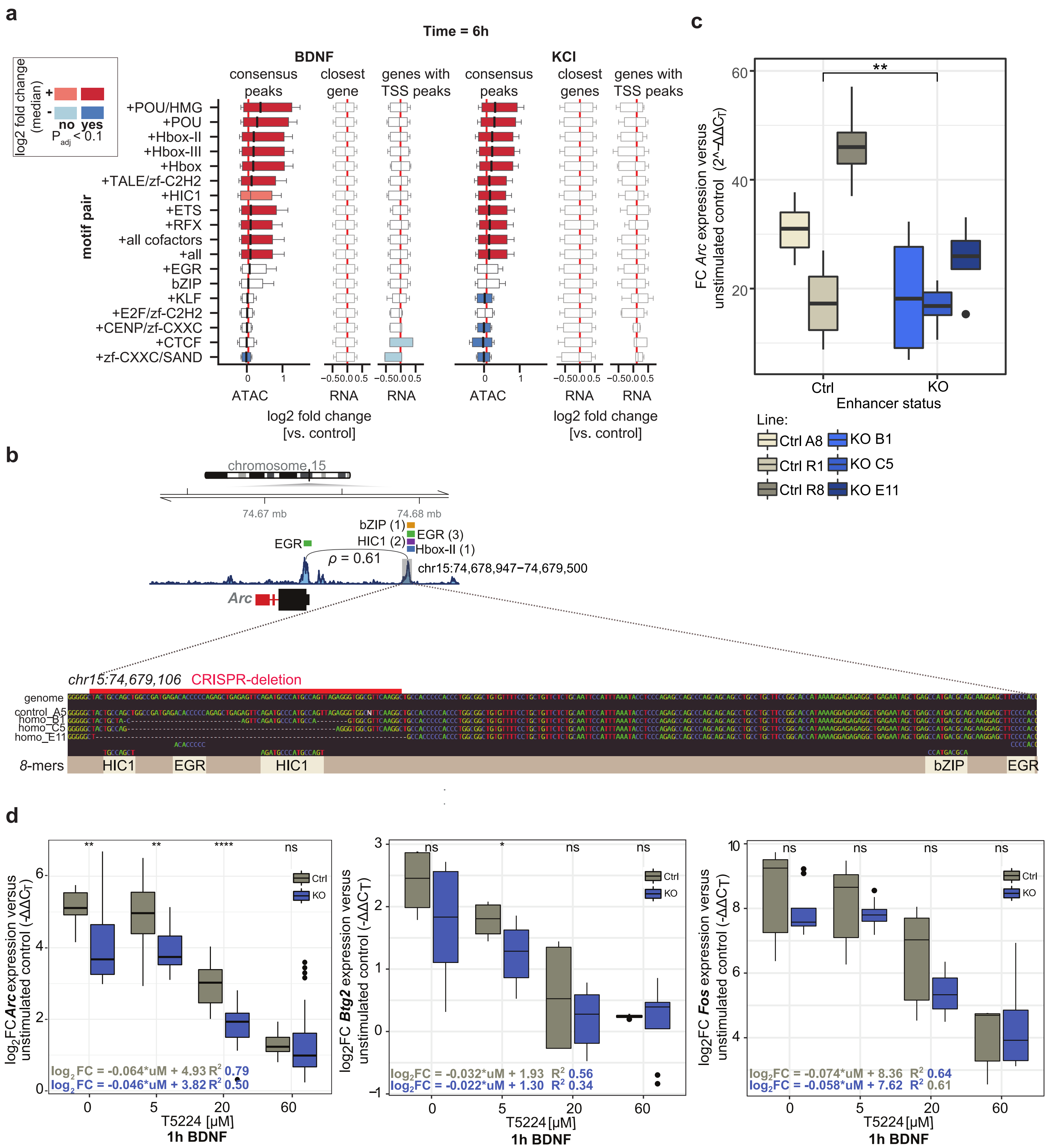

Appendix Figure S9 Validation of Fos and Egr1 binding to Arc enhancer.

(**a**) Annealing positions of primer sets designed to identify binding on the putative Arc enhancer, specific to BDNF stimulation.

(**b**) Fos and (**c**) Egr1 enrichment on Arc TSS and Arc enhancer identified by ChIP-qPCR. Negative control loci are telomeres and a gene-desert area in chromosome 6. Fold change over input is calculated as 2^-dCt sample to input (N=3 for Egr1; N=1 for Fos; error bars as s.d.; ., *, **= P < 0.1, 0.05 and 0.01, respectively, ns: not significant, two-sided t-test with control as reference group)

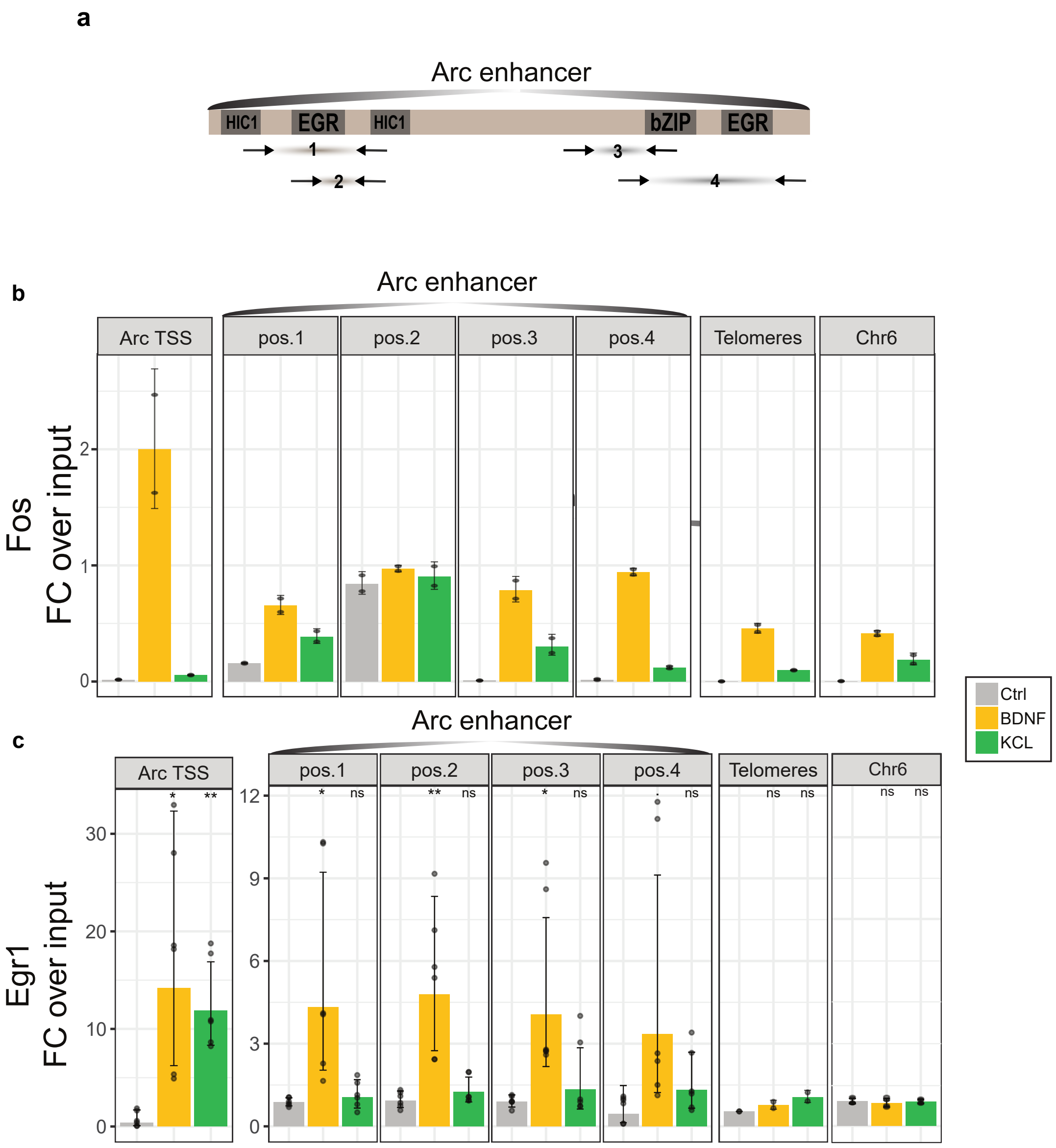
a
Arc enhancer
HIC1
EGR
HIC1
bZIP
EGR
1
2
3
4
b
Arc enhancer
Fos
FC over input
Arc TSS
pos.1
pos.2
pos.3
pos.4
Telomeres
Chr6
0
1
2
Ctrl
BDNF
KCL
c
Arc enhancer
Egr1
FC over input
Arc TSS
pos.1
pos.2
pos.3
pos.4
Telomeres
Chr6
0
10
20
30
0
3
6
9
12
*
**
*
ns
**
ns
*
ns
ns
ns
ns
ns
ns

Appendix Figure S10 Association between promoter-exon CTCF-loops, chromatin DA-peaks and differential usage of exons.

(**a**) Enrichment scores for convergent CTCF motifs in gained DA-peaks, closing DA-peaks and unchanged peaks associated with introns (*left*) and exons (*right*). Annotations are based on HOMER. Asterisks indicate Fisher's exact test *P* value significance, adjusted by Benjamini Hochberg procedure (*, **, *** = P < 0.05, 0.01 and 0.001, respectively). Circle color and size annotations as in (b)

(**b**) Exon log2 fold changes between BDNF and KCl as quantified by DEXSeq(Anders *et al*, 2012) (**Methods**). Dots indicate differentially used exons (DUE; orange), DUE with promoter-exon CTCF loops (reds) or unchanged exons (gray). Labels indicate genes and exon names with both features, selected for experimental validation

(**c**) *(top)* Genome tracks for *Stxbp5* and *Cpe201*. ATAC-seq tracks indicate DA-peaks (red = gained; blue = closing) and CTCF tracks indicate presence of motifs (pink = ChIP-seq peak; blue = motif). Below gene models, reference DUE exon position (red) is highlighted, and next it a block indicating a control exon (gray) used for comparison is highlighted. *(middle)* Box plots indicate fold change ratios between reference exon and control exon fold changes 1h after treatment with BDNF (orange), KCl (green), and control (gray). Asterisk indicates significant changes versus control (adjusted *P* < 0.1; *t*-test, two-sided). (*bottom*) Normalized RNA counts (asterisk = significant log2 fold change versus control).

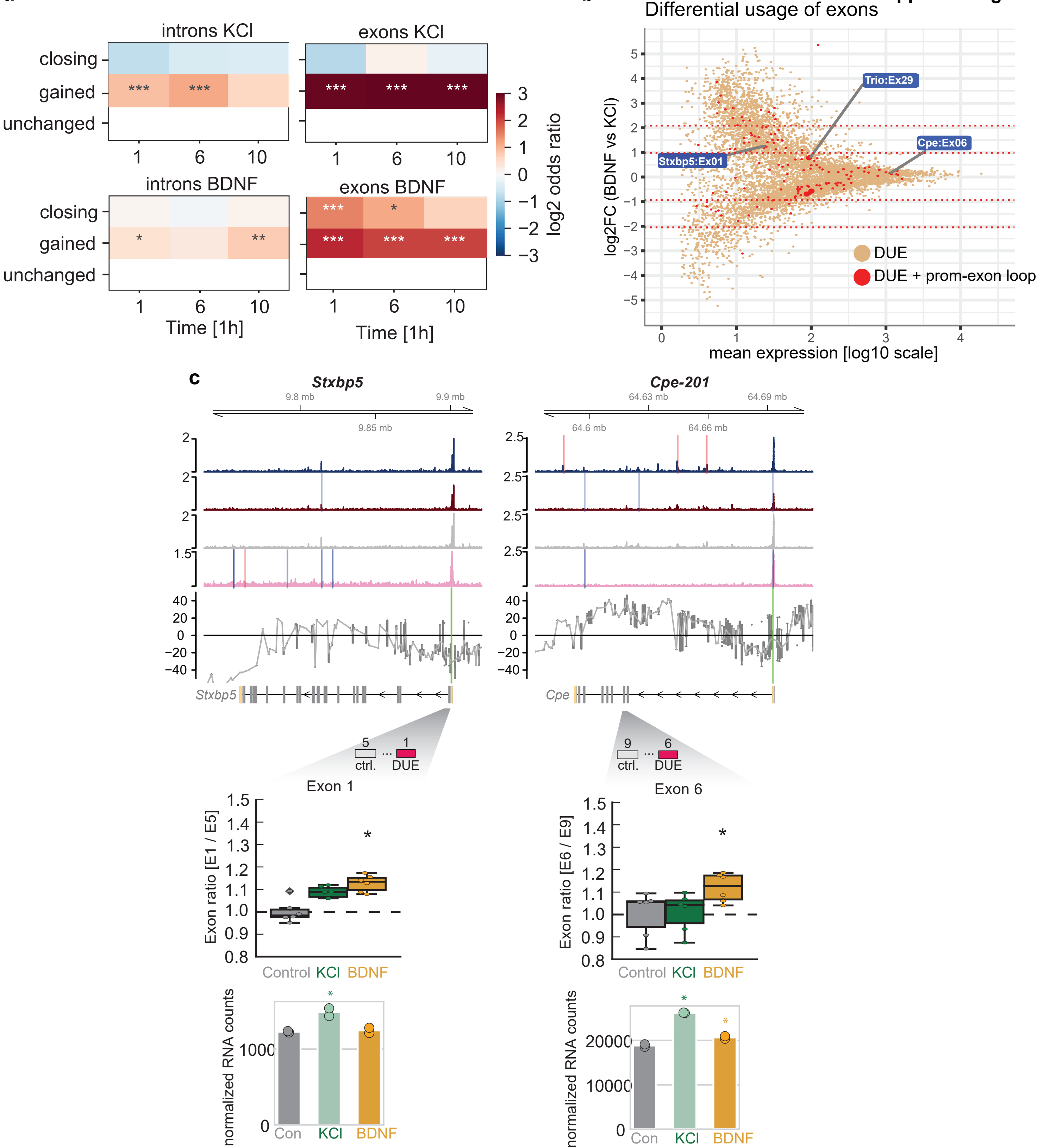
a
introns KCl
exons KCl
introns BDNF
exons BDNF
closing
gained
unchanged
Time [1h]
log2 odds ratio
b
Differential usage of exons
log2FC (BDNF vs KCl)
mean expression [log10 scale]
Trio:Ex29
Stxbp5:Ex01
Cpe:Ex06
DUE
DUE + prom-exon loop
c
Stxbp5
Cpe-201
9.8 mb
9.9 mb
9.85 mb
64.63 mb
64.69 mb
64.6 mb
64.66 mb
ctrl.
DUE
Exon 1
Exon 6
Exon ratio [E1 / E5]
Exon ratio [E6 / E9]
Control KCl BDNF
normalized RNA counts
Con KCl BDNF

Appendix Figure S11 Disease Ontology associations for mouse DA-peaks and Partitioning heritability analysis of mouse primary cortical neurons and hiPSC-derived neurons.

(**a**) Genes Ontology enrichment for genes associated with DA-peaks, linked based on GREAT(McLean *et al*, 2010). Circle size indicates effect size (log2 Odds Ratio). Colors indicate significance. *Y*-axis color labels indicate sources of ontology information.

(**b**) Associations between mouse consensus and DA-peaks from cortical neurons and human GWAS terms based on summary statistics. Legends as Figure 5a. GWAS traits labeled as associated with neuronal function are on top of the heatmap. GWAS related to non-neuronal traits are at the bottom of the heatmap.

Appendix Figure 11

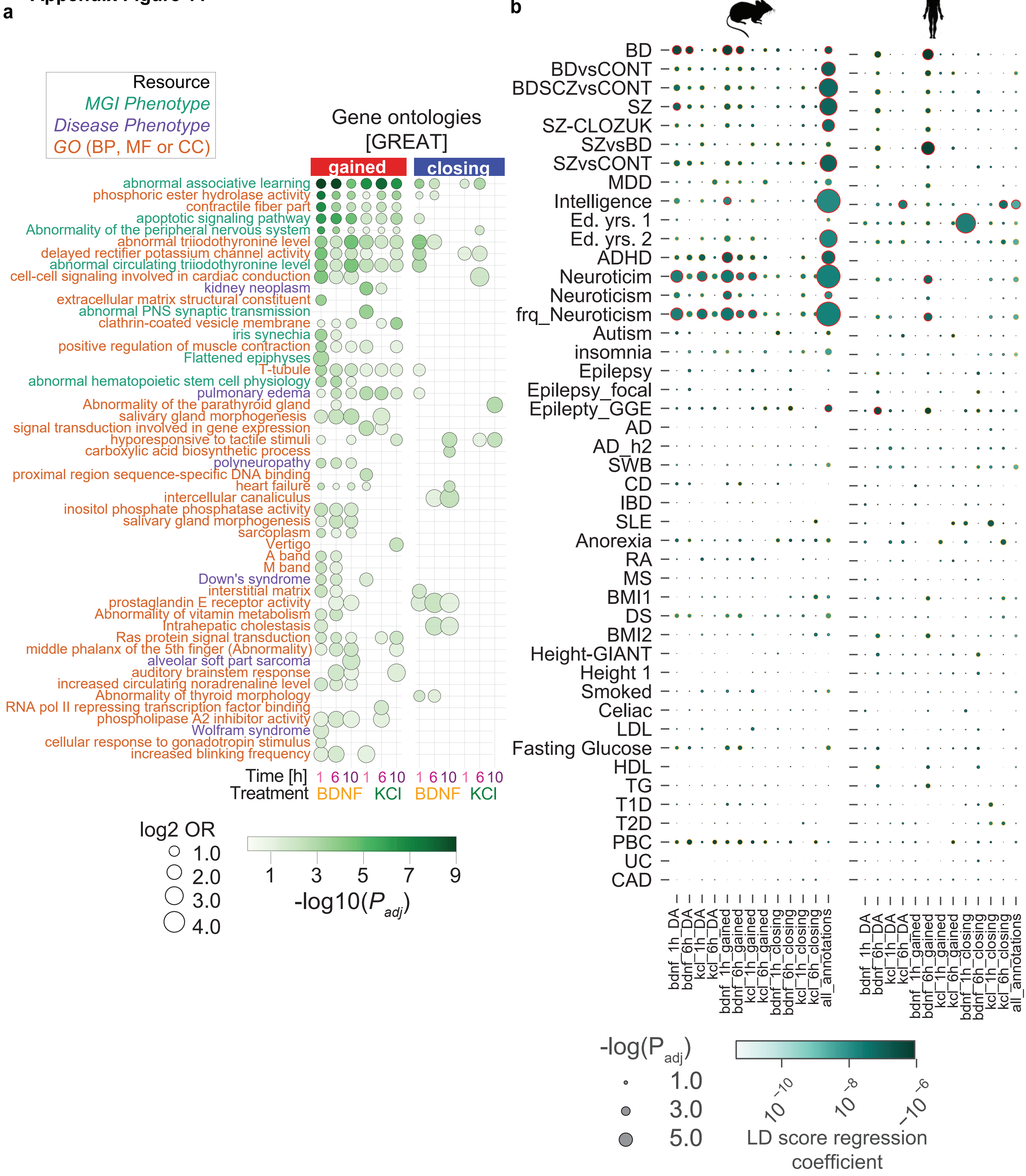

Appendix Figure S12 Histological staining of mouse and hIPSC-induced neurons and differentially accessible peaks upon treatment with BDNF and KCl

(**a**) Representative images of primary cortical mouse neurons at DIV10 stained for markers of neuronal maturity including MAP2 and Tau as well as synaptic markers Synapsin1/2, Synaptophysin, and PSD95. Nuclei are indicated by DAPI staining. Scale bars correspond to 50 μm.

(**b**) Representative images of iNeurons induced from human iPSCs at DIV13 after induction. The EGFP staining proves the efficient lentiviral delivery of NGN2-EGFP used for neuronal induction. Human iNeurons were stained for markers of neuronal maturity including MAP2, TUBIII and Tau as well as the synaptic marker Synapsin1/2. Nuclei are indicated by DAPI staining. Scale bars correspond to 50 μm.

(**c**) Principal component analysis visualization of chromatin accessibility changes in hIPSC-induced neurons.

(**d**) Number of DA-peaks recovered for each time point and stimulation category when compared versus control hIPSC-derived neurons. Gained (new) and closing (new) indicate peaks observed as differentially accessible for the first time in that time point, whereas gained and closing indicate DA-peaks already observed as DA in a previous time point.

(**e**) Hierarchical clustering of normalized counts obtained from ATAC-seq consensus peaks (*left*) and only DA-peaks (*right*).

.

Appendix Figure 12

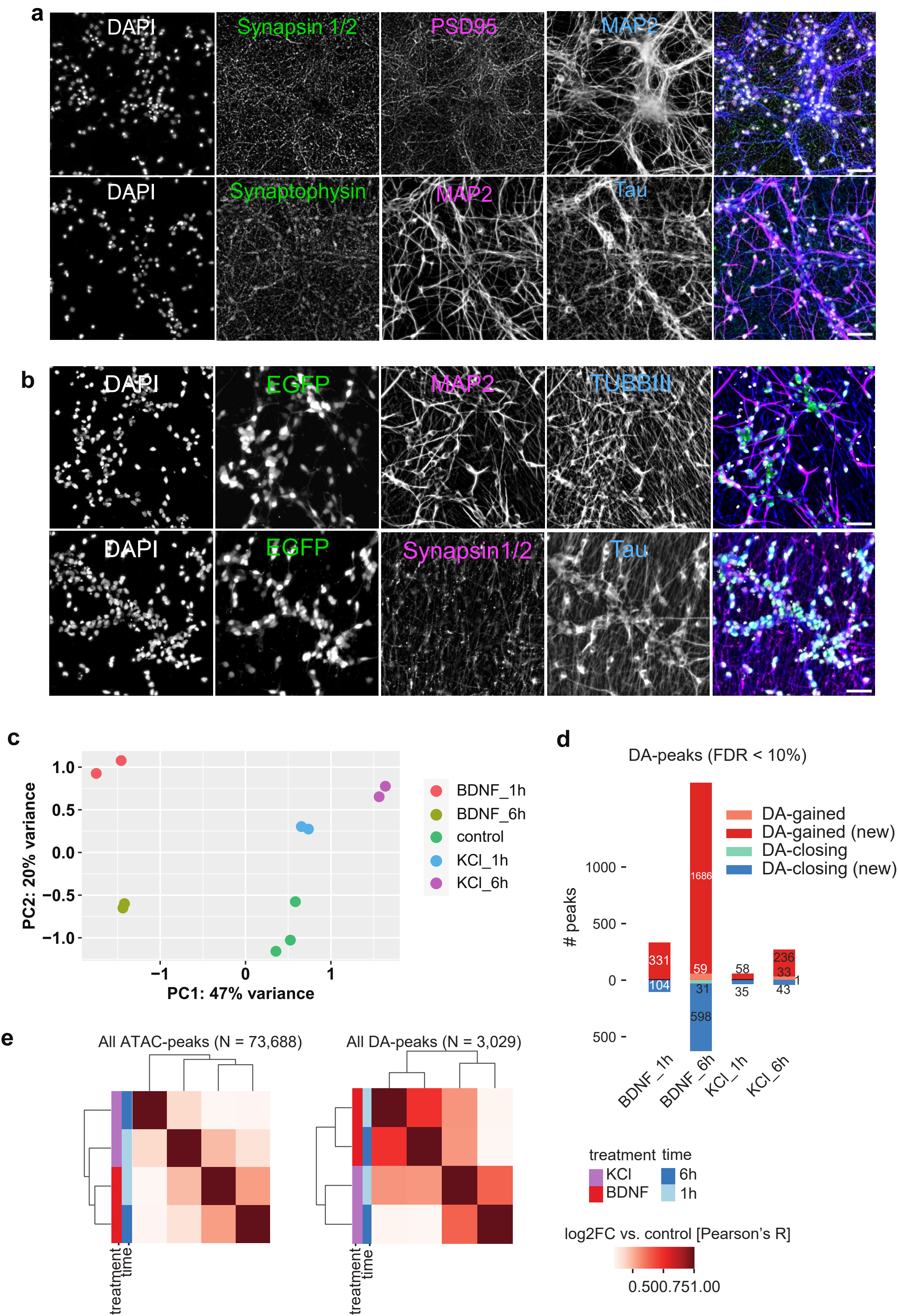

Supplement: Supplementary file 1 — Appendix [file MSB-18-e10473-s005.pdf]
