# Supplementary material for: Comparative chromatin accessibility upon BDNF stimulation delineates neuronal regulatory elements
Source: Mol Syst Biol. 2022 Aug 23;18(8):e10473. doi: 10.15252/msb.202110473 (PMC9396287; doi:10.15252/msb.202110473)
Supplement: Supplementary file 7 [file MSB-18-e10473-s006.pdf]

molecular systems biology

# Comparative chromatin accessibility upon BDNF stimulation delineates neuronal regulatory elements

Kyung Min Noh, Ignacio Ibarra, Vikram Ratnu, Lucia Gordillo, In-Young Hwang, Luca Mariani, Kathryn Weinand, Henrik Hammarén, Jennifer Heck, Martha Bulyk, Mikhail Savitski, and Judith Zaugg
**DOI: 10.15252/msb.202110473**

*Corresponding author: Kyung Min Noh (kyung.min.noh@embl.de) , Judith Zaugg (judith.zaugg@embl.de)*

## Review Timeline:

| | |
|---|---|
| Submission Date: | 27th May 21 |
| Editorial Decision: | 15th Jul 21 |
| Revision Received: | 4th May 22 |
| Editorial Decision: | 31st May 22 |
| Revision Received: | 28th Jul 22 |
| Accepted: | 1st Aug 22 |

*Editor: Maria Polychronidou*

## Transaction Report:

(Note: With the exception of the correction of typographical or spelling errors that could be a source of ambiguity, letters and reports are not edited. Depending on transfer agreements, referee reports obtained elsewhere may or may not be included in this compilation. Referee reports are anonymous unless the Referee chooses to sign their reports.)

Manuscript Number: MSB-2021-10473, Comparative chromatin accessibility upon BDNF-induced neuronal activity delineates neuronal regulatory elements

Thank you again for submitting your work to Molecular Systems Biology. We have now heard back from the three referees who agreed to evaluate your study. Overall, the reviewers acknowledge that the topic and findings are relevant. They raise however a series of concerns, which we would ask you to address in a major revision.

I think that the recommendations of the reviewers are rather clear and therefore I do not see the need to repeat the points listed below. All issues raised would need to be satisfactorily addressed. Please feel free to contact me in case you would like to discuss in further detail any of the issues raised, I would be happy to organise a video call.

On a more editorial level, we would ask you to address the following points:

Reviewer #1:

Ibarra et al. provide a comprehensive view how BDNF impacts on gene expression and chromatin dynamics in embryonic mouse primary as well as in mouse and human stem-cell-derived neurons. Samples were harvested after 1h, 6h and 10h upon BDNF stimulation and compared to KCl treatment, which also activates activity-driven transcriptomic changes in neurons. Of note, RNA-seq and ATAC-seq was performed, providing a comprehensive view on transcriptomic and chromatin dynamics as well as their interplay. The authors use state-of-the-art bioinformatic analyses and justify well every analysis, providing plain interpretations of results. TF motifs and TF cooperativity was also analyzed. Here, the authors also study protein levels by quantitative proteomics and experimentally validate their conclusions using the Arc gene as an example. Finally, the authors perform a correlation analyses of the identified BDNF and KCl-responsive chromatin loci with neuropsychiatric traits taken from published GWAS datasets. This part is less convincing as the authors' data was derived from embryonic primary neurons and immature stem-cell-derived neurons and projected on psychiatric traits that occur in mature neurons in aged patients. This biomedical link is not entirely plausible. Nevertheless, this multi-omics study provides a high-resolution system-level view on BDNF and KCl activation in neurons, which is novel and extremely useful to the field. The manuscript is well written and comprehensive (except the biomedical shortcoming).

Major points:
- The limitations of correlating the novel findings with GWAS data of neuropsychiatric traits must be included. The maturity and electrical activity level of the analyzed neurons is different and hence general conclusions are not convincing. BDNF could also impact differently on in vivo neurons due to the interactions with other cell types within the neural networks.

Minor points:
- Fig. 1c shows 772 (366+406) DE-genes at 10h after BDNF stimulation. The text states 722 (page 3)
- Fig. 2b shows 1533 closed DA-peaks after 1h if KCl stimulation, but text states 1522 (page 4)
- Supplementary Fig. 9d. This figure depicts the correlation between ATAC- and DA-peaks between 4 samples of hiPSCs-induced neurons. In the correlation plots, replicates 1 and 2 are reported, but they also correspond to the different time points after stimulation. The "replicate" annotation of the samples appears incorrect.
- the term "neuronal activity" normally refers to electrical activity, meaning functional features of neurons. The authors' projection on gene regulation and transcription (for example in the abstract) can easily be misperceived in the neuroscience community.

Reviewer #2:

In the manuscript titled "Comparative chromatin accessibility upon BDNF-induced neuronal activity delineates neuronal regulatory elements", Ibarra and colleagues performed an extensive comparison of the chromatin accessible landscape and transcriptional programs induced by BDNF stimulation and KCl-induced depolarisation. To compare both stimuli, they generated ATAC-seq and RNA-seq profiles upon each treatment at 1h, 6h and 10h using murine primary cortical neurons. BDNF stimulation resulted in large-scale chromatin remodelling, specifically at enhancers, followed by changes in gene expression. In contrast, KCl depolarisation induced a transcriptional response followed by delayed chromatin remodelling at enhancers, some of which are also remodelled upon BDNF treatment. The authors further explored the TF networks underlying the chromatin responses, and observed that the bZIP motif, bound by pioneer TFs, and thus associated with chromatin opening, was the most enriched motif for both stimuli. The network of TFs that co-operate at differentially accessible regions in each stimulus is different, suggesting stimulus-specific roles for those TFs. Finally, the authors place their work in the context of human disease and observe an enrichment of disease-associated variants at conserved regions between mouse and human genomes, and principally, at regions of increased accessibility upon BDNF stimulus, suggesting a link for BDNF activation and neuropsychiatric traits. The manuscript contributes to the understanding of how different neuronal activation mechanisms influence the chromatin landscape and transcriptional programs of neurons upon activity-dependent stimuli. Three major points require revision before publication.

Major points:

1. One concern is the choice of sample collection times, especially to consider that time dynamics of transcription and chromatin responses to KCl and BDNF stimulation are likely different. The manuscript requires a justification for the choice of time points. From the data presented, it seems likely that KCl response is quick and possibly already fading at the first (1h) time point. The authors should consider the generation of additional RNA and ATAC measurements between untreated and 1 h. Alternatively, they need to very explicitly discuss the possibility of difference in time dynamics early in the manuscript to raise the awareness of the reader, and to help shape future studies.

2. Differential analysis compares treatment to all 6 control samples, irrespective of time point:
a. 'PCA' indicates that control neurons cluster together regardless of time point. For this reason, we compared (...) of BDNF, KCl in any time point versus all control samples.' Given that the first two principal components only explain 62% of the variance, it is likely that clustering would not be observed in multidimensional space. Choosing to compare against all control samples, irrespective of time, neglects biological complexity and will give rise to false positive DE genes. We strongly recommend that differential analyses of treatment vs. control performed for RNAseq and ATACseq replicates should be done for each time point

separately to determine differential genes.

3. The chosen statistical thresholds need to be more stringent:
RNA-seq: The authors use 0.1 as an FDR cutoff and end up with ~ 5000 DE genes which is probably 1/3 to 1/2 of all genes expressed. Given the concern of false positive results (2a), a more stringent FDR of 0.05 or 0.01 would be appropriate to focus the analysis only on the most robust differential genes/peaks.

We also identify the additional points:

4. Introduction:

a. '..., chromatin responses to BDNF stimulation have not been analyzed.' Statement needs to be toned down. Comprehensive genome-wide understanding of BDNF / KCl treatment on chromatin response was missing, but individual loci have been well studied.
b. '... many of which overlap with BDNF stimulation.' a reference is missing for this statement.

5. Results, 1st chapter (BDNF triggers a biphasic expression response):
a. title: 'BDNF triggers a biphasic expression response'. Biphasic is an overstatement given that three time points were characterized and selection of time points are not well founded. Term biphasic needs to be removed.
b. 'but DE-genes decreased at 6h and 10h in both KCl and BDNF.' From this statement, it is not clear that few time point specific DE genes appear at 6 and 10h, because the overall number of DE genes is highest at 6h.

6. Results, 2nd chapter (BDNF alters regulatory elements within chromatin):

a. Fig 2F. The clusters 3-7 are not mentioned in the text. If these peaks are not important, why are 10 clusters presented? Would a lower number of peaks be more appropriate??

b. From the methods, it is not clear why the authors chose to cluster the DE genes and DA peaks into 10 clusters. For Fig. 1d, clusters 6-8 seem to display similar patterns. Same goes for Fig. 2f clusters 4-6. An elbow plot might help to choose a better number of clusters.

c. Fig. S 2a: The use of the same color scale and used shapes as in Fig S 1a for 'Treatment' and 'Time' would facilitate understanding of the results.

d. 'Based on a study linking neuronal activity response to the three-dimensional conformation of the genome (Beagan et al, 2020), we integrated the DA-peaks with published Hi-C maps generated across neurodevelopment (Bonev et al, 2017)'. Beagan et al., 2020 have shown that de-novo loops form at key loci upon neuronal stimulation. The cortical neurons from Bonev et al. are untreated and only pre-established loops/contacts would be observed in this data. This relevant reservation towards the used data needs to be considered and discussed in the manuscript.

e.Could the authors please elaborate on what was correlated in Fig. S 3a? Were all DA peaks used for that analysis or only open 'gained' or 'closing'? Does this figure not show that when having two DA-peaks, the difference between having 'no' Hi-C-loop and a cell type specific loop is smaller?

7. Results, 3rd chapter (Neuronal chromatin dynamics affect gene expression):
a. 'We found that gained DA-peaks at 1h post-BDNF induction were significantly correlated only to genes already up-regulated at 1h (Z-score > 6; Supplementary Figure 4c; Supplementary Data 2)'. It is unclear whether Fig. 2b or S4c is explained with that statement. These two plots seem to visualize the same data, but numbers are inconsistent. It also sounds as if Z-score > 6 was chosen as a threshold for significance, while determining significance with an ANOVA test seems to be t.

8. Results, 4th chapter (TF motifs underlying chromatin responses):
'Some 69% of DA-peaks appearing after stimulation are explained by one of the 16 over-represented TF motifs'.
a. Please tone down the claim, as 'explained' is an oversimplification. Did the authors make sure that these TFs were expressed?
b. Fig. 3b: There is redundancy in the motifs (AAT and CGC). Have the authors considered redundancy of binding motifs?
Fig. 3b: Why is ROC-AUC >= 0.55 highlighted?
c. In the mass spectrometry analysis, the authors compare BDNF vs. KCl. In all other analyses, each of the treatments were compared to the control neurons. The BDNF ~ KCl comparison would be blind to effects that are common to both treatments. Why was this comparison approach chosen here?
d. Could a correlation of mass spectrometry of chromatin-bound fraction over expression level of the 192 TFs be presented as this might be relevant for the community?
e. Fig. S 5b is missing legend of the circle size.
f. 'CTCF showed invariable expression levels, consistent with its ubiquitous expression and structural role in the genome

(Phillips & Corces, 2009).' Could the authors comment on that observation?
f. Fig. 3d: BDNF should be removed from the mass spec figure because cells have been treated with it.
h.'The increases in Fos protein were lower after 1h following KCl induction, which may contribute to the delayed response observed at enhancers in KCl.' Do the enhancers that show delayed binding have an enrichment for the Fos motif?

9. Results, 5th chapter (bZIP and TF cooperativity in induced gene expression):
a. 'Chromatin regions that open early in response to bZIP'. Statement needs toning down.
b. Fig. 4b: Missing numbers in KCl treatment, right panel.
'... had a Hi-C contact' sounds vague. Could the authors please provide a definition?
e. The results for the CRISPR screen are relevant but not well explained and presented. How were motifs chosen and could results of all KO strains be presented for a reader to fully understand the intentions and observations? A schematic drawing of the different KO strains would help to understand which motifs were deleted.
f. Fig. 4d: To give a sense of the actual transcription level, the expression levels in wt and treatment for each of the clones should be shown side by side.
g. 'Deleting different numbers of HIC1 motifs did not affect the level of Arc gene reduction'. This claim claim would need to be supported by a figure (see above).

10. Results, 6th chapter (Differential neuronal gene exon usage):
a. 'Promoter exon CTCF loop' how were loops defined? Needs to be stated if taken from Bonev et al.

b. 'We found enrichment for several neuronal traits for our full set of ATAC-seq peaks ...'. Clarify if the found signals intronic, exonic or intergenic?

c. Fig. S 9a: Syt1 signal in human looks unspecific.

11. Discussion:
Typo 'Systematic' instead of 'systemic'

12. Methods:
a. RNA-seq computational data processing:
Does 'library size normalized gene expression levels' as DESeq2 input mean TPM here? Using unnormalized counts would be the correct input.

b. 'We compared the enrichment of gene ontology terms (...), using all mapped genes as genome background.' Does this mean that if a single read maps to a gene, this gene would be considered expressed? A more stringent filter needs to be applied to determine the background gene set. It could either be genes transcribed > 1 TPM in at least 1 replicate, or considered expressed by DESeq2.

c. Genomic data co-variation and loop data analysis:
'We defined Distal Regulatory Elements (DREs) as regions less than 2kbp and no further than 50Kbp from TSS regions.' Possibly a typo, and the authors mean 'more than 2 kbp'.

Reviewer #3:

Ibarra et al., carried out temporal ATAC-seq and RNA-seq profiling in cultured neurons upon either BDNF or KCl (depolarization) treatments, and identified BDNF- vs. KCl-dependent differentially accessible chromatin regions and gene expression programs. To support their findings, the authors also carried out proteomics and CRISPR-mediated genome editing of the Arc enhancer. In addition, they analyzed correlations between activity dependent chromatin accessibility and human neuronal disease traits including a subset of neuropsychiatric disorders.

Studying the time-course of differential chromatin accessibility and transcriptional changes of BDNF- vs. KCl-treated neurons definetely provide interesting new information and datasets. However, this work needs significant revision before it can be considered for publication.

Major comments:
1. Comparative analysis of chromatin accessibility induced by distinct types of neuronal stimulation is definetely of interest and should be presented as the main focus of this work. On the other hand, my main criticism concerns the general conclusions about the BDNF- vs. KCl-induced chromatin regulations. It is unclear to which extent chromatin profiling from an in vitro culture system has general value regarding BDNF- vs. KCl(depolarization)-induced transcriptional programs in vivo. Indeed, distinct KCl-treatment protocols already differentially affect activity-response gene regulation (e.g. varying the concentration of KCl, or brief vs. sustained KCl treatment; PMID: 29681534; PMID: 28634074). Moreover, selection of activity-response genes is highly dependent on the cellular context (e.g. discussed in PMID 30359600). In this respect, the Discussion is rather brief and

superficial and can be improved by taking into account these considerations.
2. To validate the cooperativity of bZIP and EGRs in Arc enhancer regulation, the authors should delete the bZIP motif in ESCs as well (Fig. 4c,d, Fig S6b).
3. Fos and Egr ChIP-seq should be carried out in cells treated with the different conditions to validate the differentially accessible (DA) binding sites identified in the study.
4. While Fos mRNA levels appear higher for KCl- than BDNF-treated neurons at 1 hour (Fig. 1d, 3c), proteomics data shows the opposite. The authors did not provide a convincing explanation for this.

Minor comments:
1. Fig. 1d, heatmaps. If I understand correctly, the authors show Z-scores of fold change induction. If this is the case, I cannot distinguish whether each gene is up- or down-regulated as compared with non-treated control neurons. If they want to use Z-scores for heat maps, they should visualize normalized read numbers (e.g. cpm) of both the treated and non-treated (control) samples.
2. A few missing citations of relevant studies: PMID: 26595656, PMID:19116276, PMID:19193899
3. Neuronal activity controls BDNF expression, and neuronal activity-dependent BDNF expression triggers synaptic plasticity and other changes in neurons. On the other hand, it is misleading to say: 'BDNF-induced neuronal activity' as e.g. mentioned in the title.

We sincerely thank the reviewers for their constructive comments, which we have used to improve and revise the manuscript. Our point-by-point responses are provided below. We also provided electronic notebook links in the rebuttal wherever relevant to explain our computational analyses. Our changes in the revised manuscript are clearly described as *Italic* in the Action part of each point-by-point response and as purple-colored text in the revised manuscript.

# Reviewer #1:

Ibarra et al. provide a comprehensive view how BDNF impacts on gene expression and chromatin dynamics in embryonic mouse primary as well as in mouse and human stem-cell-derived neurons. Samples were harvested after 1h, 6h and 10h upon BDNF stimulation and compared to KCl treatment, which also activates activity-driven transcriptomic changes in neurons. Of note, RNA-seq and ATAC-seq was performed, providing a comprehensive view on transcriptomic and chromatin dynamics as well as their interplay. The authors use state-of-the-art bioinformatic analyses and justify well every analysis, providing plain interpretations of results. TF motifs and TF cooperativity was also analyzed. Here, the authors also study protein levels by quantitative proteomics and experimentally validate their conclusions using the Arc gene as an example. Finally, the authors perform a correlation analyses of the identified BDNF and KCl-responsive chromatin loci with neuropsychiatric traits taken from published GWAS datasets. This part is less convincing as the authors' data was derived from embryonic primary neurons and immature stem-cell-derived neurons and projected on psychiatric traits that occur in mature neurons in aged patients. This biomedical link is not entirely plausible. Nevertheless, this multi-omics study provides a high-resolution system-level view on BDNF and KCl activation in neurons, which is novel and extremely useful to the field. The manuscript is well written and comprehensive (except the biomedical shortcoming).

Major points:
- The limitations of correlating the novel findings with GWAS data of neuropsychiatric traits must be included. The maturity and electrical activity level of the analyzed neurons is different and hence general conclusions are not convincing. BDNF could also impact differently on in vivo neurons due to the interactions with other cell types within the neural networks.

**Response:** We agree with the reviewer that a culture model system is unable to address the full complexity of the in vivo situation. As the reviewer suggested, we have added a section describing the limitations of the use of in vitro neurons for BDNF stimulation and GWAS data analyses (**Discussion**).
Regarding the issue of applying early neuron data to psychiatric traits that occur in mature neurons in aged patients, we want to emphasize that neuropsychiatric traits, especially SCZ and BD, have been strongly associated with accessible regulatory elements detected at the very early stages of human neurodevelopment (de la Torre-Ubieta et al, 2018; Hook & McCallion, 2020). Also, individuals with BD or SCZ showed reduced levels of BDNF(Ray et al, 2014; Lima Giacobbo et al, 2019), suggesting a link between BDNF-mediated gene regulation and these

traits. We believe that a part of the BD and SCZ-associated early accessible regulatory elements is captured by our model system of neurons stimulated with BDNF.

**Action:** we have added the following points to the Discussion section of our revised manuscript:

- Limitation statement for the in vitro BDNF stimulation (**Discussion - second paragraph)**: *".. Nevertheless, we note that the stimulation effects on chromatin are highly diverse in response to many parameters, such as stimulation strength and duration(Tyssowski et al, 2018; Fukuchi et al, 2017; Joo et al, 2016), neuronal type and connectivity(Fuentes-Ramos et al, 2021; Harabula & Pombo, 2021) and possibly cellular memory to previous stimuli(Yap & Greenberg, 2018)."*

- Limitation statement for the GWAS analysis (**Discussion - Last paragraph)**: *"Limitations to our analysis lie in the inherent distinctions between in vitro and in vivo systems, represented by the difference in neuronal maturation states, interactions between different cell types, and formation of neuronal networks, which can affect the chromatin response to BDNF. Therefore, chromatin events dependent on these parameters that only occur in vivo could not be captured in our setting."*

Minor points:
- Fig. 1c shows 772 (366+406) DE-genes at 10h after BDNF stimulation. The text states 722 (page 3)

**Response and action:** We thank the reviewer for noting this typo. We have updated the number to 772 on page 3. We also include notebooks to verify these numbers for the processed data "notebook/figure1_degenes.ipynb".

- Fig. 2b shows 1533 closed DA-peaks after 1h if KCl stimulation, but text states 1522 (page 4)

**Response and action**: We have updated the number to 1533 on page 3 and include notebooks to verify these numbers for the processed data "notebooks/figure2_dapeaks.ipynb".

- Supplementary Fig. 9d. This figure depicts the correlation between ATAC- and DA-peaks between 4 samples of hiPSCs-induced neurons. In the correlation plots, replicates 1 and 2 are reported, but they also correspond to the different time points after stimulation. The "replicate" annotation of the samples appears incorrect.

**Response**: We thank the reviewer for noting this label mistake. Old Supp 9d **(now Appendix Figure 11d)** indicates the correlation between log2 fold changes between all peaks and the union of all DA-peaks for each stimulation/time versus control samples. For that reason, the indicated values are an estimated mean based on all replicates.

**Action:** We have revised this figure to remove the mistaken replicates label.

- the term "neuronal activity" normally refers to electrical activity, meaning functional features of neurons. The authors' projection on gene regulation and transcription (for example in the abstract) can easily be misperceived in the neuroscience community.

**Response and action**: We thank the reviewer for highlighting this terminology confusion. We have updated the term **neuronal activity** to **neuronal stimulation**, which is used in a previous study (Joo et al, Nat. Neuroscience 2015). This update has been applied to all sections of the re-submitted manuscript (title/abstract/main text) thus, we do not label any specific location.

# Reviewer #2:

In the manuscript titled "Comparative chromatin accessibility upon BDNF-induced neuronal activity delineates neuronal regulatory elements", Ibarra and colleagues performed an extensive comparison of the chromatin accessible landscape and transcriptional programs induced by BDNF stimulation and KCl-induced depolarisation. To compare both stimuli, they generated ATAC-seq and RNA-seq profiles upon each treatment at 1h, 6h and 10h using murine primary cortical neurons. BDNF stimulation resulted in large-scale chromatin remodelling, specifically at enhancers, followed by changes in gene expression. In contrast, KCl depolarisation induced a transcriptional response followed by delayed chromatin remodelling at enhancers, some of which are also remodelled upon BDNF treatment. The authors further explored the TF networks underlying the chromatin responses, and observed that the bZIP motif, bound by pioneer TFs, and thus associated with chromatin opening, was the most enriched motif for both stimuli. The network of TFs that co-operate at differentially accessible regions in each stimulus is different, suggesting stimulus-specific roles for those TFs. Finally, the authors place their work in the context of human disease and observe an enrichment of disease-associated variants at conserved regions between mouse and human genomes, and principally, at regions of increased accessibility upon BDNF stimulus, suggesting a link for BDNF activation and neuropsychiatric traits. The manuscript contributes to the understanding of how different neuronal activation mechanisms influence the chromatin landscape and transcriptional programs of neurons upon activity-dependent stimuli. Three major points require revision before publication.

Major points:

1. One concern is the choice of sample collection times, especially to consider that time dynamics of transcription and chromatin responses to KCl and BDNF stimulation are likely different. The manuscript requires a justification for the choice of time points. From the data presented, it seems likely that KCl response is quick and possibly already fading at the first (1h) time point. The authors should consider the generation of additional RNA and ATAC measurements between untreated and 1 h. Alternatively, they need to very explicitly discuss the possibility of difference in time dynamics early in the manuscript to raise the awareness of the reader, and to help shape future studies.

**Response:** We thank the reviewer for raising the issue of time points and the possibility of the fading gene expression response. To examine whether the KCl response is already fading at the first (1h) time point, we performed an RT-qPCR experiment and accessed the expression level of several canonical immediate early genes (Arc, Egr1, Fos, Npas4) at earlier time points (0, 15min, 30min, and 60min). Both KCl and BDNF induced stimulation-dependent genes with increasing transcriptional levels over time (**Reviewer Figure 1**, new **Appendix Figure S1a**). These results indicate that at 1h, our neurons are likely in an induction phase of gene expression for both KCl and BDNF stimuli. Our time-course result upon KCl is consistent with a previous report, which found most immediate early gene expression peaked between 1h and 2h after KCl stimulation (Tyssowski *et al*, 2018)

Given that the qPCR results show a constant increase up to 1h, additional high-throughput sequencing at times earlier than 1 h would not provide much extra information. We decided not to generate additional RNA and ATAC measurements, and instead, as the reviewer suggested, we highlighted the possibility of early dynamics to the reader.

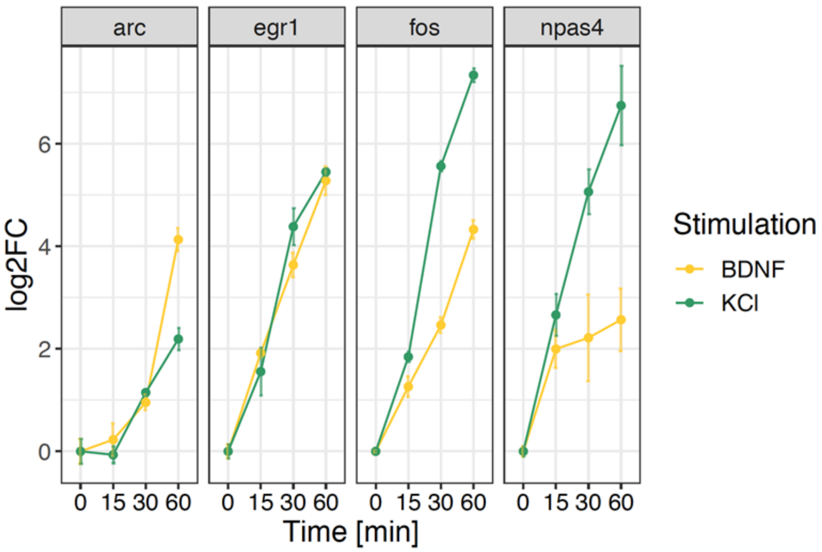


***Reviewer Figure 1** (new Appendix Figure S1a). qPCR measurement of immediate early gene expression levels after 15, 30, and 60 minutes of stimulation with BDNF or KCl, normalized to RPL-13 (housekeeping gene) and unstimulated neurons (0min) (n=2 biological replicates, error bars as s.d.)*

**Action:** Updates in text (**Results - BDNF triggers a biphasic expression response - 1st paragraph)**:

- *"Both stimuli induced transcription of immediate early genes within minutes of treatment, but the transcriptional levels constantly increased with different dynamics in the first hour of stimulation (**Appendix Figure S1a**), in agreement with previous reports applied KCl stimulation (Guzowski et al, 1999; Tyssowski et al, 2018). Therefore, to access early transcriptional responses, we examined 1h after treatment as the first time point, followed by 6h and 10h to capture later transcriptional events (Tyssowski et al, 2018)"*

2. Differential analysis compares treatment to all 6 control samples, irrespective of time point:
a. 'PCA' indicates that control neurons cluster together regardless of time point. For this reason, we compared (...) of BDNF, KCl in any time point versus all control samples.' Given that the first two principal components only explain 62% of the variance, it is likely that clustering would not be observed in multidimensional space. Choosing to compare against all control samples, irrespective of time, neglects biological complexity and will give rise to false positive DE genes. We strongly recommend that differential analyses of treatment vs. control performed for RNAseq and ATACseq replicates should be done for each time point separately to determine differential genes.

**Response**: We appreciate the reviewer for this comment. As suggested, we performed additional differential expression analysis using each time-point-specific control and observed very similar fold-changes and numbers of DE genes to what our current analyses provide (see Reviewer Figure 3). We like to re-emphasize that there is no clustering of the control samples according to time points based on the correlation of all normalized counts (using either the union of all DE-genes or all genes; new **Appendix Figure S1 d**). Also, on the PCA we did not find any clustering of the controls by time point for the first two dimensions. We extended the PCA analysis to the first four dimensions and found again no evidence for time-point specific clustering of control samples (see Reviewer Figure 2).
Given that combining all control samples allows for a better estimation of the dispersion per gene, we would argue it is better to consider all control samples together and keep the analysis as we have done.

**Action:** i) We extended the PCA analysis to test whether **variability** captured by PC 1-4 is associated with the time point of control samples. Specifically, we have assessed the clustering of the control samples using all PC1 to PC4 vectors (**Reviewer Figure 2**, new Appendix Figure S1b), which explain 85.1% of the total variability. None of the bi-dimensional plots indicates a time-specific separation in the control samples. These results suggest that biological variation between time points in control samples is minimal.
Notebook link: figure1_control_variability_rld.ipynb

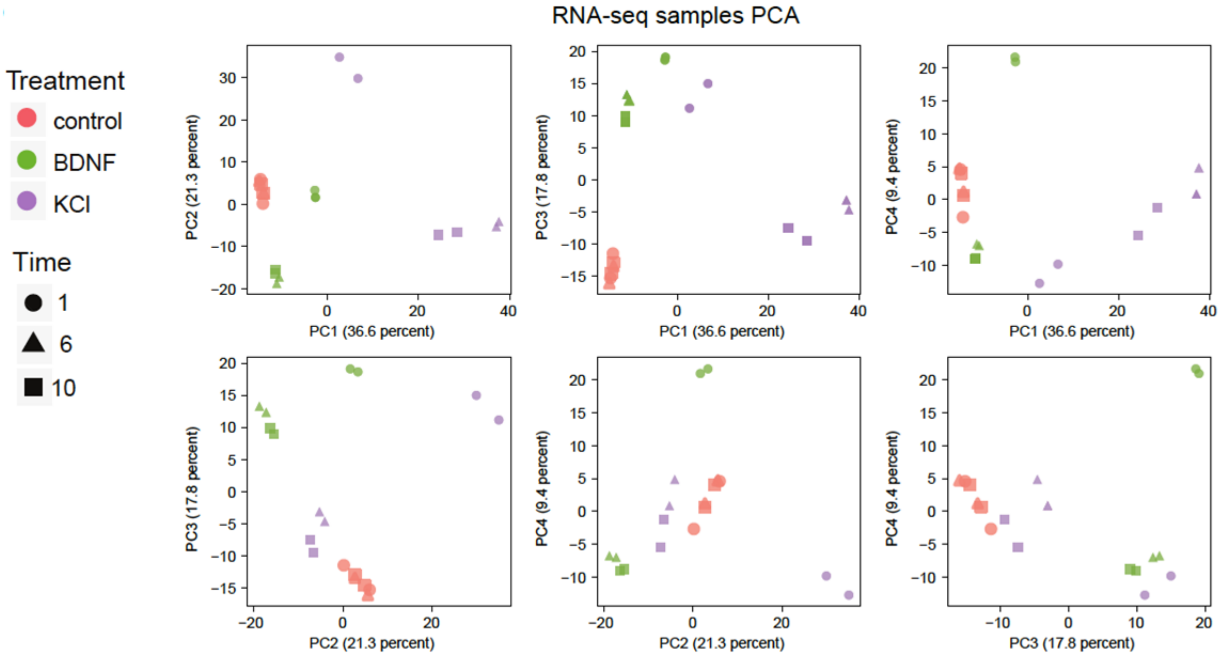


***Reviewer Figure 2*** *(new Appendix Figure S1b). PCA visualizations based on rld-normalized RNA-seq data, using PC pairs between PC1 and PC4. Colors and shape legends are indicated in the first panel.*

ii) **Agreement of log2 fold change estimates and number of significant DE-genes**: The main reason for considering *all control* samples together is that it allows a better estimation of dispersions per gene. Below (**Reviewer Figure 3**), we have compared the log2 fold changes of DE-genes at each time and treatment, between time-specific control (y-axis) and all controls (x-axis). In general, we observed strong positive correlations for DE-genes that are found in both comparison types, and complete overlap in the sign of log2 fold changes between DE-genes and controls. This analysis indicates that the estimates for gene expression changes are not biased by the usage of all control samples, and can overall detect more DE-genes due to an improvement during the dispersion estimation step.

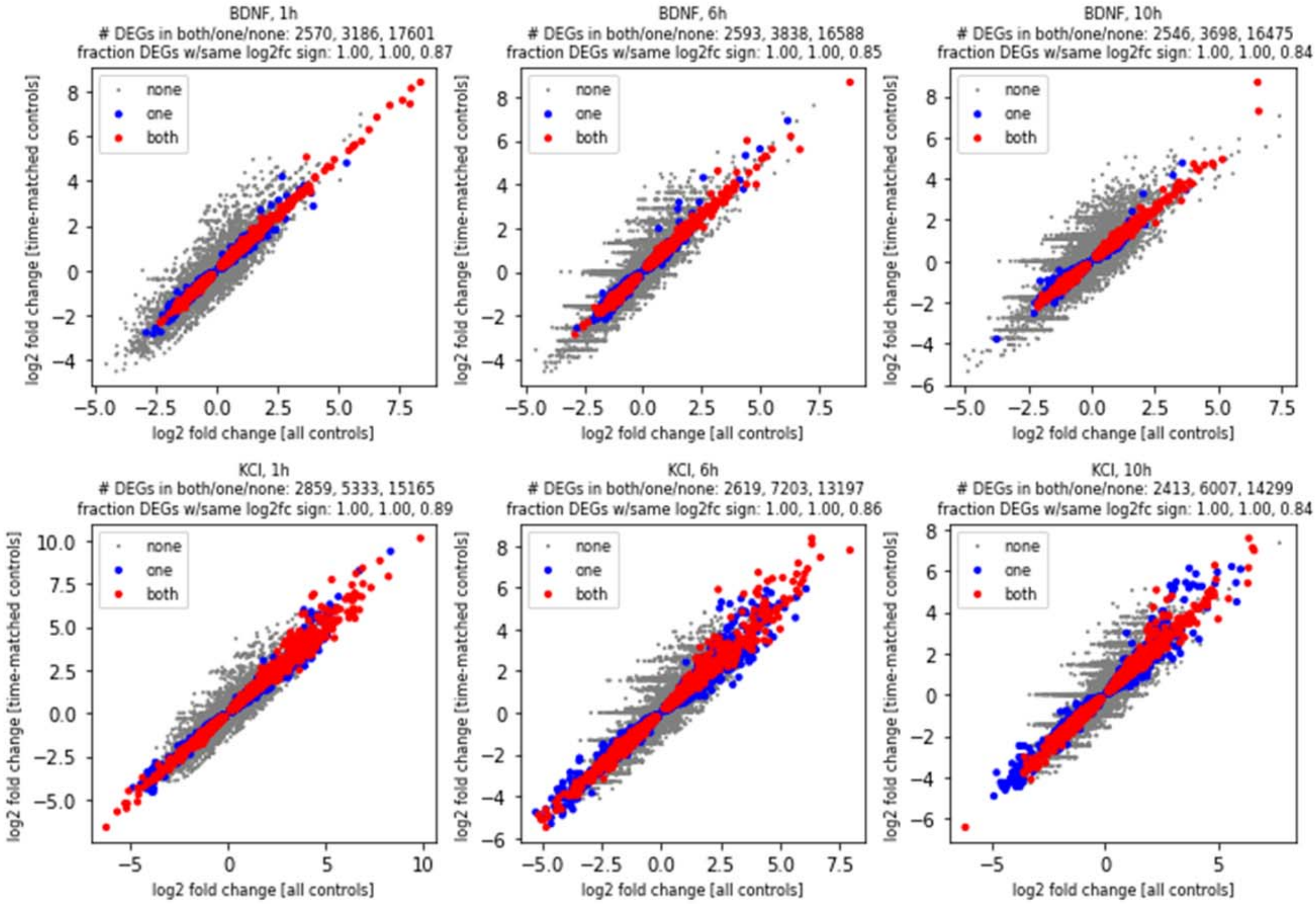


***Reviewer Figure 3.*** *Log2 fold changes obtained using all control samples (x-axis) and time-specific control samples (y-axis). Red points indicate DE-genes reported in both analyses, whereas blue indicates DE-genes found only at one analysis. Numbers on top indicate the number of points and proportion of genes in the same direction, whenever the gene is DE-genes in both samples, one sample, or none.* *Notebook link*: **figure1_degenes.ipynb**

**Actions:**

- Updated Methods (**RNA-seq computational data processing**): "*As PCA analyses indicate that control neurons cluster together regardless of time point, we processed raw counts for these samples using DESeq2, and compared gene expression levels of BDNF, KCl at any time point versus all control samples…*"
- Erratum (update Appendix Figure S1a, now S1b): The explained PC1/PC2 variances in here vary slightly in the original manuscript as the 1st version was generated along with other samples from related work. This PCA panel is the only one affected. We have updated this panel in the main manuscript, and we apologize for the inconvenience.

3. The chosen statistical thresholds need to be more stringent: RNA-seq: The authors use 0.1 as an FDR cutoff and end up with ~ 5000 DE genes which is probably 1/3 to 1/2 of all genes expressed. Given the concern of false positive results (2a), a more stringent FDR of 0.05 or

0.01 would be appropriate to focus the analysis only on the most robust differential genes/peaks.

**Response**: In this study, we deliberately use a low stringency threshold to have a larger set of genes/peaks for comparing peak and gene expression variation over time. Most of our analyses are based on the clustering of expression patterns across time points. Thus the differentially expressed genes are a way to select the most interesting genes, which then get further split into sub-groups (clusters) based on their temporal behavior. Furthermore, a large portion of all the DE-genes (71.2%) reported with FDR=0.1 is also present with FDR=0.01.
Regarding ATAC-seq peaks, a smaller fraction of DA-peaks is recovered with FDR=0.01 (39.1%), yet the main dynamics across time are virtually the same as described in Fig 2b (**Reviewer figure 4**). Thus, we do not expect the FDR threshold to be a major confounder in our analyses and conclusions.

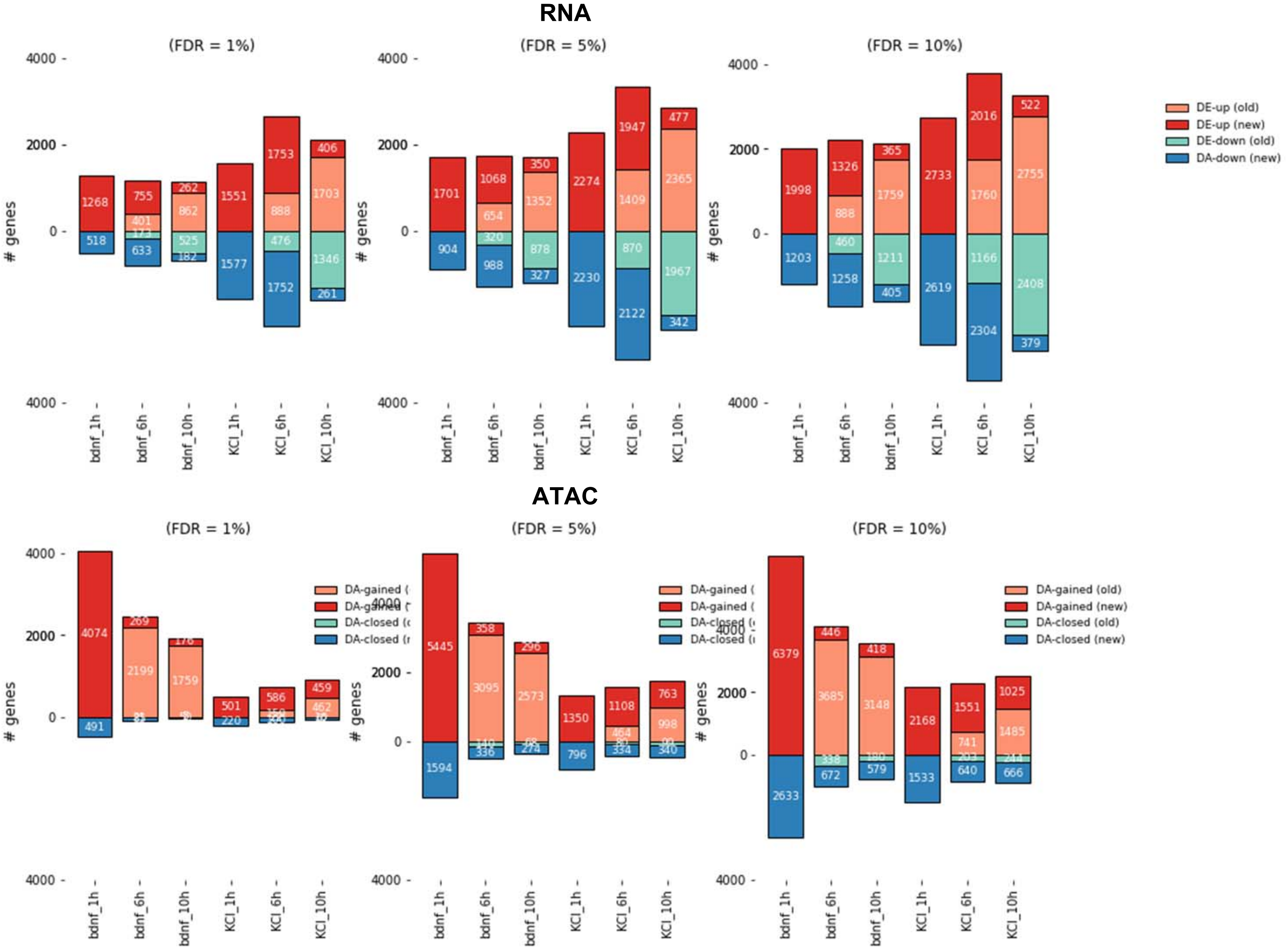

***Reviewer Figure 4.*** *Counts for up/down DE-genes (upper row) and gained/closing ATAC-seq peaks (bottom row) with FDR thresholds of 1% (left), 5% (middle) and 10% (right).*

Notebook link: notebooks/figure1_count_degenes_diff_thr.ipynb

We also identify the additional points:

4. Introduction:
a. '..., chromatin responses to BDNF stimulation have not been analyzed.' Statement needs to be toned down. Comprehensive genome-wide understanding of BDNF / KCl treatment on chromatin response was missing, but individual loci have been well studied.

**Response**: We agree with the reviewer and have added it to the text.

**Action:** We updated the main text (**Introduction, 2nd paragraph**) to**:** "*While BDNF-induced chromatin regulation and gene expression have been studied in individual loci(Tuvikene et al, 2016; Alder et al, 2003), genome-wide responses to BDNF stimulation have not been analyzed.*"

b. '... many of which overlap with BDNF stimulation.' a reference is missing for this statement.

**Response and action**: We have included a reference (**Introduction, 3rd paragraph**): (Liu *et al*, 2018).

5. Results, 1st chapter (BDNF triggers a biphasic expression response):
a. title: 'BDNF triggers a biphasic expression response'. Biphasic is an overstatement given that three time points were characterized and selection of time points are not well founded. Term biphasic needs to be removed.

**Response**: We adopted the term ‘biphasic’ as it has been previously used in the literature to describe the gene expression pattern induced by KCl stimulation at equivalent time points (1h, 6h, and 10h; (Yap & Greenberg, 2018; Tyssowski *et al*, 2018). We do think that the term biphasic best captures what we are observing, i.e. that genes can be classified as early (1h) and late (6, 10h) responders. We hope the reviewer approves this.

b. 'But DE-genes decreased at 6h and 10h in both KCl and BDNF.' From this statement, it is not clear that few time point specific DE genes appear at 6 and 10h, because the overall number of DE genes is highest at 6h.

**Response**: We thank the reviewer for this point since it allows us to clarify the statement. In this statement, we wanted to emphasize the number of genes that were newly differentially expressed at 6h and 10h. Most DE-genes are already differentially expressed at time-point 1h. Thus, even though bars (total number of genes) are higher in 6h and 10h versus 1h, the lighter subsection within the bar plots, which indicate the number of newly discovered differentially

expressed genes, are smaller. For instance, newly DE-genes at 6h (1334 new upregulated for BDNF, 2024 for KCl) and 10h (366 up BDNF, 523 for KCl) are less than at 1h (1998 up BDNF, 2733 for KCl). We have updated the section and text to highlight this point better in the resubmitted manuscript.

**Action:** We updated the main text to: *"but the number of genes that became newly differentially expressed at 6h or at 10h decreased in both KCl (N = 5,352/4,344/905 for 1, 6 and 10h, respectively) and BDNF (N = 3,201/2,597/772)."*

6. Results, 2nd chapter (BDNF alters regulatory elements within chromatin):
a. Fig 2F. The clusters 3-7 are not mentioned in the text. If these peaks are not important, why are 10 clusters presented? Would a lower number of peaks be more appropriate??

**Response**: We thank the reviewer for pointing this out. We explained the number of clusters below (b). We noted clusters 3-6 could refer to less accessible peaks and down-regulation of genes by either BDNF or KCl, or both, that can be teased apart by observing the size of the circles and darkness of color (log2 odds ratio and significance). We added a description of clusters 3-7 to the main text.

**Action:** we have added the following (**Results - Neuronal chromatin dynamics affect gene expression. 3rd paragraph**): *"A set of BDNF-specific early-response peaks associated with early-response genes in BDNF (cluster 1; 1,623 peaks) and a similar peak cluster for KCl (cluster 2; 1,972 peaks) contain a large fraction of promoters. Together with cluster 1, another set of peaks (cluster 7; 1,360 peaks) includes opening peaks associated with upregulated genes in response only to BDNF, at all time points. Sets of shared DA-peaks that were affected by both BDNF and KCl (clusters 8-10; 4,293 peaks) showed a faster response in BDNF than in KCl for accessibility and gene expression, and are composed mainly of distal elements. In the case of closing peaks associated with downregulated genes, we could identify 4 different trends (clusters 3 to 6; N = 2,163/1,258/1,530/1,367, respectively). Cluster 3 comprises KCl-specific peaks corresponding to a great extent to promoters (in all time points) whilst clusters 4 and 5 are BDNF-specific and are mostly annotated as distal elements, with cluster 5 depicting early-response downregulated genes. Similar to clusters 8-10, cluster 6 represents shared closing peaks, with a large proportion of distal elements."*

b. From the methods, it is not clear why the authors chose to cluster the DE genes and DA peaks into 10 clusters. For Fig. 1d, clusters 6-8 seem to display similar patterns. Same goes for Fig. 2f clusters 4-6. An elbow plot might help to choose a better number of clusters.

**Response:** We thank the reviewer for this suggestion. Even though our selection of clusters for heatmaps is partly guided by clustering metrics, we ultimately decided to explore and expand the granularity described by significantly discovered GO terms (Figure 1d), and associations with DE-gene trends (Figure 2f):

- In Figure 1d, there are specific ontologies that are not visualized in clusters 6-8 when *k=8*, and can only be observed when *k*=10. As shown below (**Reviewer Figure 5**),

depending on the metric for the elbow plot (e.g., Silhouette, the Elbow method, and any other of the possible metrics we show in our notebook), we would end up with different numbers of clusters. Thus, any decision with regards to the number of clusters based on any of these metrics is somewhat arbitrary. We chose the number of clusters in a way that allows visualizing the GO complexity we have detected in the most granular way possible, and at least 4-5 non-redundant clusters were shown for downstream interpretation purposes.

- For Figure 2f, the selection is done in a similar way: exploring the number of associations found between DE-gene trends associated with DA-peaks, and selecting the number of clusters in which the granularity of associations with DE-gene associations does not get more subdivided with increasing values of *k*.

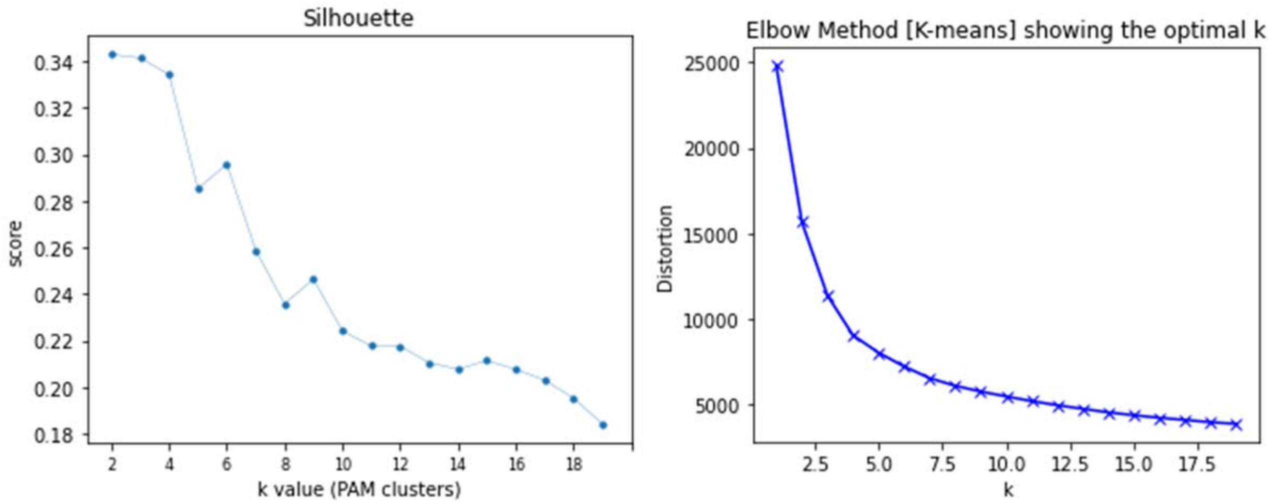


***Reviewer Figure 5*** *(Related to Figure 1d). (left) Silhouette score for PAM clustering. (right) elbow method using K-means clustering.*

**Action:** We have added the following sentence to the methods section: "*The number of clusters was decided based on the granularity of associations described by showing top 5 significantly discovered GO terms (Figure 1d), and associations with DE-gene trends (Figure 2f*)"

Scripts at: notebooks/figure1_clustering_elbow.ipynb

c. Fig. S 2a: The use of the same color scale and used shapes as in Fig S 1a for 'Treatment' and 'Time' would facilitate understanding of the results.

**Response and action:** We thank the reviewer for this observation. In the revised manuscript, we have updated the colors of Fig. S2a to match the ones in Fig. S1a (new S1b).

d. 'Based on a study linking neuronal activity response to the three-dimensional conformation of the genome (Beagan et al, 2020), we integrated the DA-peaks with published Hi-C maps generated across neurodevelopment (Bonev et al, 2017)'. Beagan et al., 2020 have shown that de-novo loops form at key loci upon neuronal stimulation. The cortical neurons from Bonev et al. are untreated and only pre-established loops/contacts would be observed in this data. This

relevant reservation towards the used data needs to be considered and discussed in the manuscript.

**Response:** We appreciate this insightful comment on the stimulated condition in Beagan et al., 2020 and Bonev et al., 2017. We agree that this is important to comment on and have done so in the updated manuscript.

**Action:** We updated the main text to include the following sentence (**Results - BDNF alters regulatory-element chromatin. Last Paragraph**). "*These Hi-C maps do not include genomic contacts that are formed de novo in response to stimulation, but encompass the pre-established loops during development…Thus, following BDNF stimulation, changes in enhancer accessibility … correlate with changes in the physical connectivity in the genome linked to neurodevelopment.*"

e.Could the authors please elaborate on what was correlated in Fig. S 3a? Were all DA peaks used for that analysis or only open 'gained' or 'closing'? Does this figure not show that when having two DA-peaks, the difference between having 'no' Hi-C-loop and a cell type specific loop is smaller?

**Response**: These values depict the correlation values between the peak normalized signals across stimulation conditions between pairs of peaks. Correlations are calculated for all pairs within 200 Kbp, and peak pairs are also grouped by the annotation of individual peaks as associated with Hi-C data: "none of the peaks is DA", "one of the peaks is DA", or "both of the peaks are DA". Finally, within each of these groups, peak pairs are further stratified based on what type of Hi-C connection was observed between them.

Regarding the last statement, this figure indeed shows that the difference between peaks without Hi-C loops and cell type-specific loops gets smaller when both peaks are DA peaks. We can think of several reasons for this, such as (i) sample size differences between groups, or (ii) peaks with more DA-peaks are also in general more variable, which can then lead to a higher correlation baseline for randomly selected peaks. Although we believe that both points are interesting data observations that could be assessed, they are not a major point in our analyses and insights. For that reason, we decided not to expand on those points in this revision.

To reiterate, the most important conclusions of this visualization are that (i) CN- and NPC-specific peaks have the highest correlation distributions between peak pairs, and (ii) the correlation of peak pairs without DA-peaks tends to increase as either of those or both peaks are DA-peaks.

**Action:** Clarified in the main text "*We observed higher correlations for loop-associated peak pairs in cortical neurons with respect to the ones associated to embryonic stem cells, or unnanotated (**Appendix Figure S3a**), suggesting a role for genome topology in co-varying regions.*

7. Results, 3rd chapter (Neuronal chromatin dynamics affect gene expression):
a. 'We found that gained DA-peaks at 1h post-BDNF induction were significantly correlated only to genes already up-regulated at 1h (Z-score > 6; Supplementary Figure 4c; Supplementary Data 2)'. It is unclear whether Fig. 2b or S4c is explained with that statement. These two plots seem to visualize the same data, but numbers are inconsistent. It also sounds as if Z-score > 6 was chosen as a threshold for significance, while determining significance with an ANOVA test seems to be t.

**Response**: We thank the reviewer for this comment, which we believe refers to the results in Figures 2e (not 2b- it shows DA-peaks without connecting to any genes) and S4c. Specifically, Figure 2e indicates the total number of genes/peaks in each time point and stimulus, while S4c is limited to the number of peaks and genes that are connected - equivalent to the shaded area connecting the left and right sides in the Sankey plot. For instance, at BDNF 1h Figure 2e shows the number of peaks (left; 1184) and genes (right; 725), of which 201 are paired; Fig S4c contains only the number of pairs (201).

Regarding statistical testing, a standard test such as ANOVA, Chi-squared, or Fisher's exact test can be suitable for the assessment of our data. However, in Chi-squared and/or Fisher's exact test, categories are dependent on each other and thus the NxM contingency matrices are not fully disentangled. Therefore, we have proposed a permutation test approach that keeps the structure of the log2 fold changes and p-values of each DA-peak and DE-gene, and is more suitable to the current data structure, given their time-course dependency.

We understand that Fisher's/Chi-squared estimates might be required for a full assessment, in this version we are providing in both Figures and EV2 data the *P*-values and effect sizes for both genes and peaks. Specifically, in EV2 we added three new columns for each peak/gene measurement: (i) odds.ratio.fisher: The odds ratio obtained for each pairing between DA-peaks and DE-genes sets, (ii) padj.fisher: The final adjusted *P*-value of all independent tests, and (iii) pearson.res: The deviation versus expected values using a Chi-squared test. Finally, we updated Figure 2e to highlight the most important associations, and indicate whether those are supported by either the permutation approach (Z-score), and/or the association based on a Fisher's exact test result (FDR=10%), after multiple test correction (*). The calculation of estimates are reproduced in our repository notebooks/sankey**.**

**Actions:**

- Updated Figure 2e labels to indicate if association is supported by our permutation approach, Fisher's exact test, or both.
- Updated EV2 to indicate Fisher's exact test and Chi-squared test Pearson residuals.
- Updated main text in 3rd Chapter and methods to highlight the usage of an FDR threshold for both approaches ("FDR=10%"). Additionally, the Methods section is updated to indicate this, at the section "Genomic data co-variation and loop data analysis.".

8. Results, 4th chapter (TF motifs underlying chromatin responses):

'Some 69% of DA-peaks appearing after stimulation are explained by one of the 16 over-represented TF motifs'.
a. Please tone down the claim, as 'explained' is an oversimplification. Did the authors make sure that these TFs were expressed?

**Response:** We thank the reviewer for this comment. We have clarified in the resubmitted version “explained by” as “associated with”. Indeed, we have verified that at least one of the TFs binding to over-represented motifs is expressed; this is summarized in Figure 3c.

**Action:** We have added the following sentence to the main text, 3rd chapter “TF motifs underlying chromatin responses”, first paragraph: **“***Some 69% of DA-peaks appearing after stimulation are associated with one of the 16 over-represented TF motifs.*”

b. Fig. 3b: There is redundancy in the motifs (AAT and CGC). Have the authors considered redundancy of binding motifs?

**Response:** Thanks for the comment. We want to emphasize that the “Motif” is summarizing multiple 8-mers associated with each TF category (not 3-mers). The prevalence of 3-mers is a visual effect, which highlights the most conserved region.

In general, 8-mers related to any of the 108 TF specificity groups have a similar content at the level of di- and tri-nucleotides, but this overlap is for longer k-mers. Based on the PBM data from Mariani et al, we are using 8-mers as these are the ones that can be readily quantified and reproduced in the *in vitro* binding of TFs, and also because it generates a distinguishable separation between clusters (Mariani *et al*. 2017). It is correct to say that there are limitations in this approach, as the partial overlap in the specificity of some TF groups, such as bZIP / NR2E.

**Actions:**

- We are explaining these details highlighted in the current manuscript (**Methods - Motif enrichment analysis, last paragraph**): **“***One limitation of motif enrichment analyses based on k-mers is the partial overlap of independent k-mer groups due to shared k-mers. In general, 8-mers overlap between the 108 TF specificity groups is reduced, given the high value of k (8). Previous work to demonstrate this used Protein Binding Microarray data and distinguished accurate separation between such datasets (Mariani et al. 2017). TF modules such as bZIP and NR2E require careful consideration, as they have a higher overlap of common 6 and 7-mers.”*
- We provide a loader of *k*-mers used in each group, that can be verified using the function “GENRE.get_kmers_by_module(kmers_dir='.’). E.g. see notebook at. notebooks/figure3_enhancer_specific_signal.ipynb

Fig. 3b: Why is ROC-AUC >= 0.55 highlighted?

**Response:** The value of ROC-AUC 0.55 is highlighted as a minimal effect size threshold (0.5 is random), to refer to interesting TF groups to follow up. This allows increasing the focus in a handful of cases where the motif prevalence differences between positive and negative sequences have the greatest magnitude, and not only the ones with the smallest p-values.

c. In the mass spectrometry analysis, the authors compare BDNF vs. KCl. In all other analyses, each of the treatments were compared to the control neurons. The BDNF ~ KCl comparison would be blind to effects that are common to both treatments. Why was this comparison approach chosen here?

**Response**: We used this volcano plot to emphasize the effect sizes of protein abundance between BDNF and KCl. Both stimuli show Fos protein as a significant TF present on chromatin, but to a greater extent after BDNF (also validated by Western blot, see Appendix Figure S6b provided to reviewer's point 8***h***). A direct comparison of BDNF/Ctrl and KCl/Ctrl is shown below (Reviewer Figure 6).

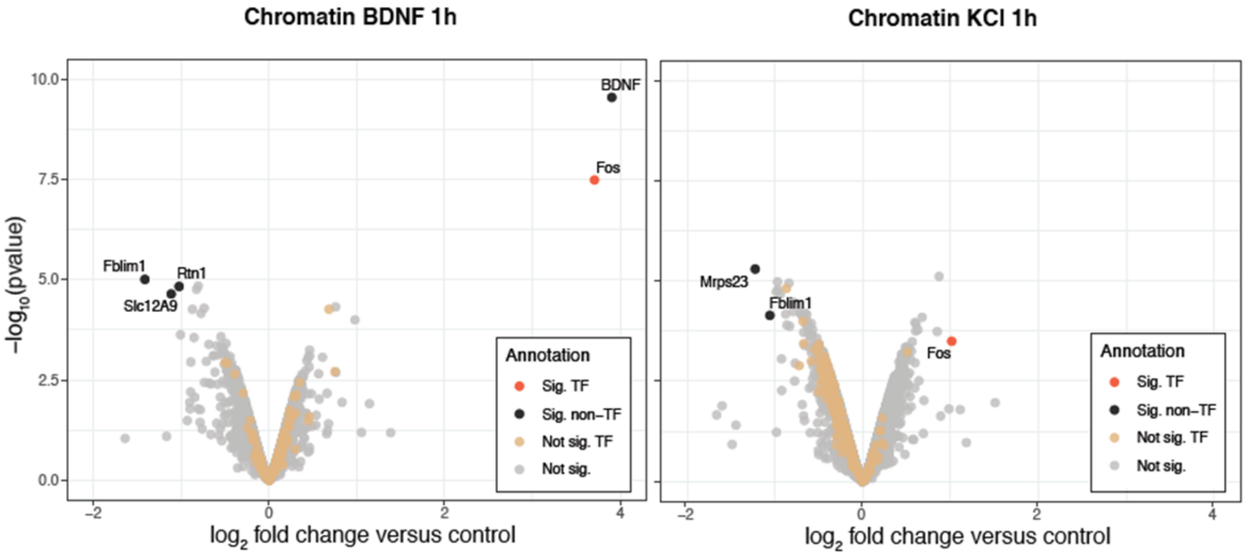


***Reviewer Figure 6****. Changes in protein abundances on chromatin after 1h BDNF (left) and KCL (right) versus unstimulated control. Proteins were annotated as significant TF (red; P < 0.05, according to CIS-BP database), significant non-TF (black), not significant TF (beige), and other not significant proteins (grey). Total detected proteins 5508, with 192 annotated TFs (n=2 biological replicates).*

d. Could a correlation of mass spectrometry of chromatin-bound fraction over expression level of the 192 TFs be presented as this might be relevant for the community?

**Response and action**: We thank the reviewer for this suggestion. We have included correlations and matched values between RNA/proteomics for the 187 TFs (out of the 192) expressing at BNDF_1h and KCl_1h and having both protein and RNA readouts. Interestingly, the highest correlations are found between protein changes after BDNF stimulation (compared to control) and changes in TF-coding genes triggered by KCl (at all timepoints) and after 1h of BDNF. Differentially abundant proteins after KCl stimulation, however, show a lower correlation

with TF and non-TF DEG in all samples. Since the proteomics dataset only comprises proteins found on chromatin, the lack of correlation of protein-RNA may indeed point out an additional layer of regulation at the post-transcriptional (probably post-translational) level that is diminishing the recruitment of these TFs in the case of KCl and not in BDNF. It is interesting to see that the correlation in TF protein and genes is maintained over time in KCl, but comparable only after 1h of BDNF, supporting our claim of a stronger role of TF transcriptional regulation at early time points of BDNF stimulation and a delayed or maintained role in KCl stimulation. The notebook to reproduce this purpose can be found in <u>notebooks/figure3_corr_protein_rna.ipynb</u>

We have now added this as a new Appendix Figure S6c-d and refer to it in the main text.

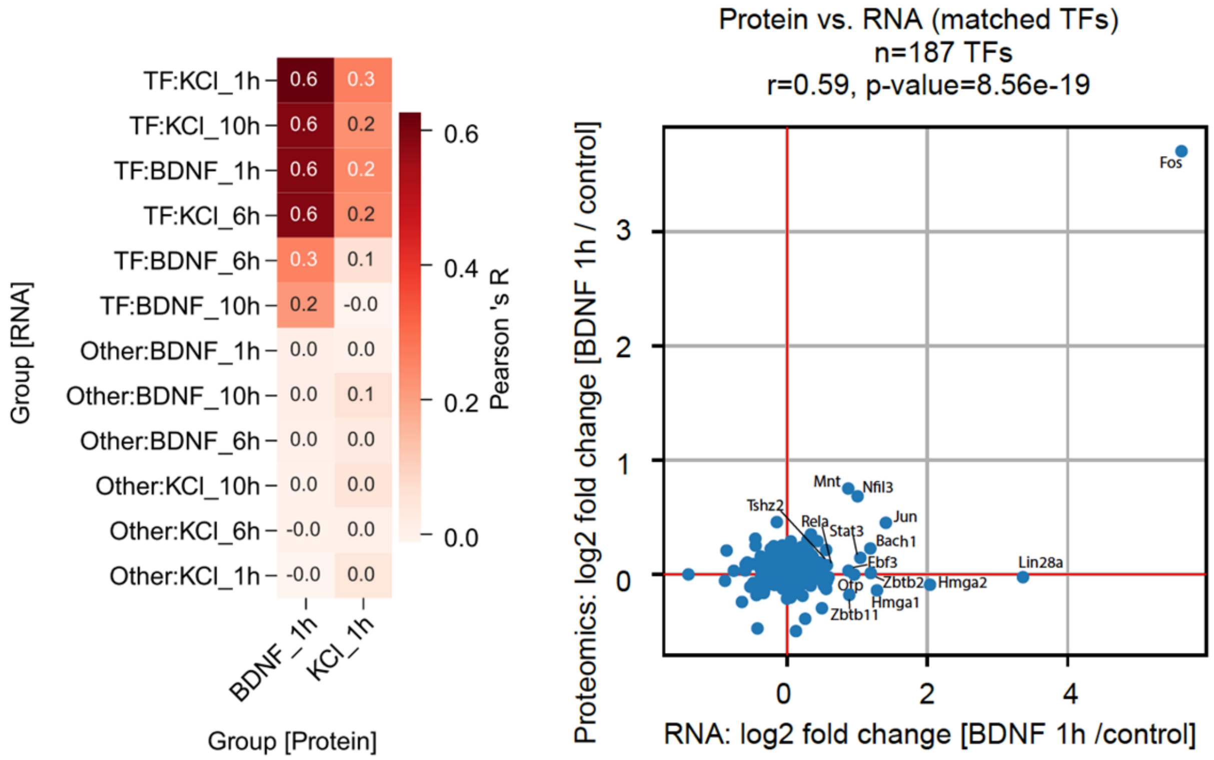


***Reviewer Figure 7*** *(new Appendix Figure S6c and d). Proteomics and RNA comparisons for Transcription Factors: (left) Linear correlation between RNA (y-label) and proteomics(x-label) overall changes versus control samples, shown as the linear correlation between readouts for both TF-related genes ('TF') and non-TFs ('Other'). (right) BDNF protein abundance change readouts for 187 matched TFs (log2 fold changes versus control) versus RNA (log2 fold changes BDNF 1h versus control). The title indicates linear correlation value and significance. Dot outlier in the top-right indicates the gene Fos. Labels indicate the top 15 TFs, sorted descendingly by the maximum value in either x- or y-labels.*

**Updates in text**: Indication of a Supplementary figure to provide these correlation plots and scatter plot comparison (**Results - TF motifs underlying chromatin responses - 3rd paragraph**) *"Among all 5,508 proteins detected, we observed an overall increase in the abundance of 192 combined TF proteins after BDNF stimulation compared to KCl (Fig. 3d, lower panel, <u>comparison between RNA and proteins in Appendix Figure S6c-d</u>)"*

***Updates in figures:*** *"Appendix Figure 6c and d, plus legend"*

e. Fig. S 5b is missing legend of the circle size.

**Response and action**: We thank the reviewer for this suggestion. In the resubmitted version, we include the circle size in the legend for the supplementary figure.

f. 'CTCF showed invariable expression levels, consistent with its ubiquitous expression and structural role in the genome (Phillips & Corces, 2009).' Could the authors comment on that observation?

**Response:** CTCF is associated with closing-chromatin peaks, but the expression is non-variable, as detected by RNA-seq. We speculate that CTCF motifs' association with DA-peaks is due to its cooperativity with other early-upregulated TFs induced by BDNF/KCl stimulation. Examples of those TFs are EGR factors and HIC1, as presented in Figure 5a.

**Action:** we updated the main text: *"CTCF is likely to function with other TFs whose expression was induced by stimulation."*

g. Fig. 3d: BDNF should be removed from the mass spec figure because cells have been treated with it.

**Response:** We agree with the reviewer that the observed BDNF signal should be associated with the treatment itself - including BDNF in the panel serves as a treatment control. We have highlighted that the signal associated with BDNF should be connected to the experimental stimulation, and not to gene expression.

**Action:** we updated the text in Figure 3d legend:
"*High levels of BDNF (blue dot) are associated with experimental stimulation."*

h.'The increases in Fos protein were lower after 1h following KCl induction, which may contribute to the delayed response observed at enhancers in KCl.' Do the enhancers that show delayed binding have an enrichment for the Fos motif?

**Response:** We thank the reviewer for this comment and have performed two additional analyses (details below). Indeed, we found that enrichment of Fos-associated motifs at enhancers (gained DA-regions (FDR=1%) overlapping with intergenic regions) is higher at later time points for KCl and earlier for BDNF (**Appendix Figure S5c**). In addition, we provide experimental evidence that Fos protein shows a delayed increase in KCl treatments (**Appendix Figure S6b**).
Detailed analyses:
**i) Computational**: We subdivided the gained DA-peaks by genomic annotations (using HOMER) and verified the enrichment of the Fos-related motif (bZIP) between sequences harboring those regions and negative regions, using ROC-AUC scores (**Reviewer Figure 8**,

new Appendix Figure S5c). Overall, regions associated with enhancers are fewer for KCl than BDNF (Intergenic), and ROC-AUC enrichments associated with enhancers (Intergenic) are significant in all cases (FDR=1%). Thus, the enrichment for Fos-associated motifs at enhancers is found at both BDNF and KCl gained DA-regions (FDR=1%), yet effect sizes are greater for BDNF than KCl (as indicated in **Figure 3b**).

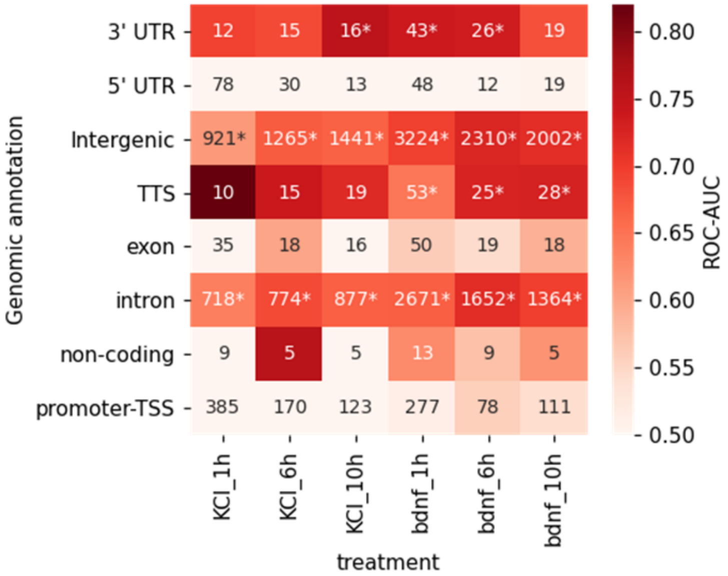


***Reviewer Figure 8*** *(new Appendix Figure S5c). Enrichment of bZIP associated motifs in gained DA-peaks stratified by genomic annotation. The number in each cell indicates the number of motifs. * indicates significance of result, using Wilcoxon tests and BH procedure for multiple testing correction.*

*Code*
*git.embl.de/rio/neuronal_activity_bdnf/-/blob/main/notebooks/figure3_enhancer_specific_signal.ipynb*

**ii) Experimental:** We tested Fos protein levels in nuclear extractions after 1h and 6h of BDNF or KCl treatment by Western blot. Fos protein levels are higher after 1h of BDNF stimulation compared to KCl (barplot, $P < 0.05$, two-sided t-test), in accordance with the mass spectrometry data. Additionally, Fos protein levels are reduced after 6 h of BDNF but rise after 6h of KCl stimulation, supporting a delayed activity of Fos in KCl compared to BDNF. We provide this result in the supplementary panel and refer to it in the main text.

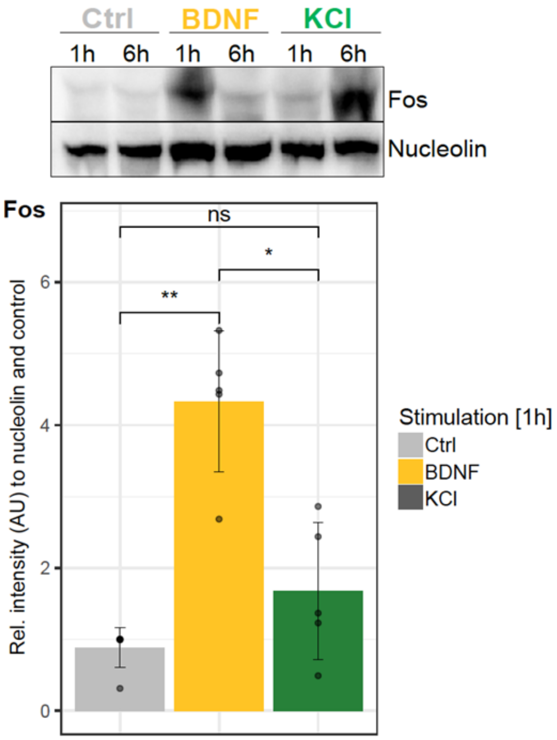


***Reviewer Figure 9*** *(new Appendix Figure S6b). Analysis of Fos protein levels in nuclear extractions after 1h and 6h of treatment by Western blot, normalized to nucleolin levels. Quantification was performed on the 1h stimulation samples with 5 independent replicates (**P <0.01, *P<0.05, ns: not significant, two-sided t-test, error bars as s.d.)*

**Action:** We have added a statement in which we claim that Fos-bound enhancers may display a delayed response to KCl (**the last paragraph in TF motifs underlying chromatin responses**): "*In addition, Fos protein showed a delayed increase in KCl treatments by Western blot (**Appendix Figure S6b**) and the enrichment for Fos-associated motifs at enhancers (intergenic regions) linked to gained DA-regions (FDR=1%) is higher at later time points for KCl and earlier for BDNF (**Appendix Figure S5c**). These results suggest that Fos-bound enhancers exert a delayed response to KCl.*"

9. Results, 5th chapter (bZIP and TF cooperativity in induced gene expression):
a. 'Chromatin regions that open early in response to bZIP'. Statement needs toning down.

**Response and action**: We updated this sentence to "Chromatin regions that open early *presumably in response to bZIP-related factors…*"

b. Fig. 4b: Missing numbers in KCl treatment, right panel.

**Response and action:** We clarify that the numbers for boxplots in left/right boxplots are the same, and the numbers are exactly the same as in the left one. We have duplicated those to avoid confusion. We thank the reviewer for their suggestion.

'... had a Hi-C contact' sounds vague. Could the authors please provide a definition?

**Response and action:** Updated to *"and had a high Hi-C contact score with the Arc gene promoter, as measured by shaman package."*

e. The results for the CRISPR screen are relevant but not well explained and presented. How were motifs chosen and could results of all KO strains be presented for a reader to fully understand the intentions and observations? A schematic drawing of the different KO strains would help to understand which motifs were deleted.

**Response:** We thank the reviewer for this comment, which allows us to clarify the details of the CRISPR results. Previously, we had included a schematic drawing of the three KO lines to inform the deleted motifs (**Appendix Figure S6b, new S7b**) but did not accurately describe it. We agree with the reviewer that further clarifications of motif choice and KO lines are necessary to provide the reader with a more precise description, which we have done now (see below - also responses to f and g).

**Action:** We have updated to text: "*To test whether the co-occurrence of bZIP+TF2 could impact gene expression induced by BDNF stimulation, we focused on the Arc gene (Fig. 4c) [...]. To assess this, we aimed to remove one of the activator TF motifs such as EGR motif and determine the level of Arc induction. It is, however, complicated as each of the three EGR motifs at this enhancer region is very close to the other TF motif (first EGR motif with HBox-II, second EGR motif with HIC1, third EGR motif with bZIP). To this end, we chose to remove the second EGR motif with HIC1 as we might be able to separate the activator and repressor effect. The third EGR motif is in high proximity to bZIP, thus we chose not to disturb it. We generated mouse embryonic stem cell (mESC) clones that homozygously removed the distal genomic region [...].*"

f. Fig. 4d: To give a sense of the actual transcription level, the expression levels in wt and treatment for each of the clones should be shown side by side.

**Response and action**: As the reviewer suggested, we provide the plot showing the Arc gene expression for each of the clones (three EGR motif KO lines and three control clonal lines) upon BDNF stimulation and KCl depolarization. A biological variation appeared between clones, yet none of the clones showed CRISPR off-target effects (based on predicted off-target sites sequencing), and all had intact genomes (based on low coverage genome sequencing). The cause of variation between control lines is unclear, but the variation between EGR motif KO lines could be partly due to the additional HIC1 motif deletion (explained below).

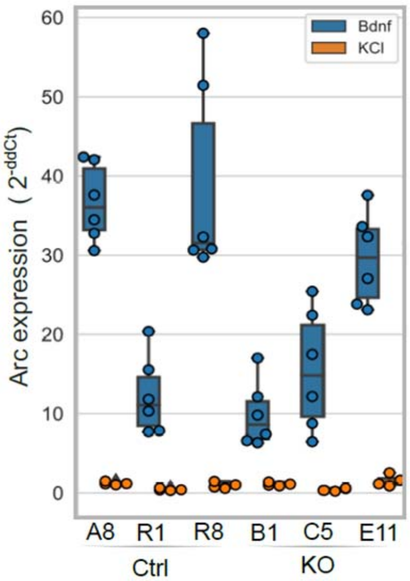


***Reviewer Figure 10*** *(new Appendix Figure S7c)**.** *Arc expression values were measured by qPCR in the different CRISPR control and Arc enhancer KO lines. Each line (3 control lines-A8, R1, R8, and 3 KO lines-B1, C5, E11) was differentiated into neurons and stimulated in biological duplicates (n=2 technical qPCR measurements)*

g. 'Deleting different numbers of HIC1 motifs did not affect the level of Arc gene reduction'. This claim claim would need to be supported by a figure (see above).

**Response:** In response to the reviewer's comment, we provided more details of the results. Specifically, all the KO lines have EGR motifs deleted. In addition to that, the C5 line has one HIC1 motif deleted, and the E11 line has two HIC1 motifs deleted (**Appendix Figure S7b**). We observed a trend of anticorrelation between the numbers of HIC1 motif deletion and *Arc* transcription induced by BDNF stimulation, suggesting a functional role of HIC1 in his enhancer (**Appendix Figure S7c**). Nevertheless, considering the clonal variation within controls, this result is not enough to fully dissect the different effects of HIC1 and EGR on *Arc* expression. Thus, we aggregated all clonal results and presented them in a simplified way in the initial manuscript. We have commented the possible effect of HIC1 acting as a repressor of *Arc* expression in the revised manuscript.

**Action:** We have updated the main text: *"In addition, when dissecting the expression levels per clonal line, we observed a trend of anticorrelation between the number of HIC1 motif deletions (additionally to the EGR motif deletion) and Arc transcription induced by BDNF, implying a role of HIC1 in this enhancer (**Appendix Figure S7c**)"*

10. Results, 6th chapter (Differential neuronal gene exon usage):
a. 'Promoter exon CTCF loop' how were loops defined? Needs to be stated if taken from Bonev et al.

**Response:** Promoter-exon CTCF loops were defined following the predictions from our previous work (Ruiz-Velasco *et al.* 2017 Cell systems). Here, a promoter-exon CTCF loop is annotations that harbor convergent CTCF motifs between the promoter and any part of the gene (intron/exon). We mentioned it in methods and figure legends but not in the main text. We have revised the main text.

**Action:** We have updated the main text: "*To test this idea, we quantified differentially used exons (DUEs) between BDNF and KCl, and found 54 genes with DUEs that harbored BDNF DA-peaks over a CTCF motif or a putative promoter-exon CTCF loop (OR=1.6 relative to non-DUE genes; P < 0.001;* ***Appendix Figure S9b****; promoter-exon CTCF loop predictions from Ruiz-Velasco et al.(Ruiz-Velasco et al., 2017)*"

b. 'We found enrichment for several neuronal traits for our full set of ATAC-seq peaks ...'. Clarify if the found signals intronic, exonic or intergenic?

**Response:** Thanks for the suggestion. In this analysis (LDSC), all DNA variants are selected together by genome blocks, regardless of whether those regions are intronic/exonic/intergenic. We clarify this in the main text to make sure the idea is properly conveyed.

**Action:** Updates in the main text: *"Testing for associations between all genome regions harboring ATAC-seq peaks, we found enrichment for several neuronal traits and little enrichment for non-neuronal traits (**Appendix Figure S10b**)"*

c. Fig. S 9a: Syt1 signal in human looks unspecific.

**Response and action:** Indeed, the Syt1 signal in human neurons is weak due to antibody unspecificity. We tried to optimize the protocol to reduce background and enhance antibody signal without success, even though Syt1 gene expression levels were similar to Map2, and even higher than PSD95 and Tubb3 (**Reviewer Figure 11**). We performed new staining using an improved protocol for sample preparation and image acquisition (see **Methods: Immunofluorescent staining of human and mouse neurons**) and new antibodies (updated in **Reagents and Tools Table**). These results (**Reviewer Figure 12**) are included in **Appendix Figure S11 a** (mouse) and **b** (human).

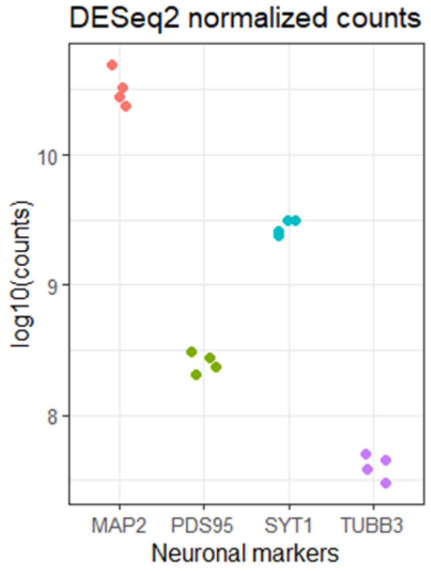


***Reviewer Figure 11****. DESeq2 normalized counts of neuronal marker genes in hiPSC-derived neurons. Data comes from an independent experiment where 4 biological replicates of unstimulated neurons were subjected to RNA-seq.*

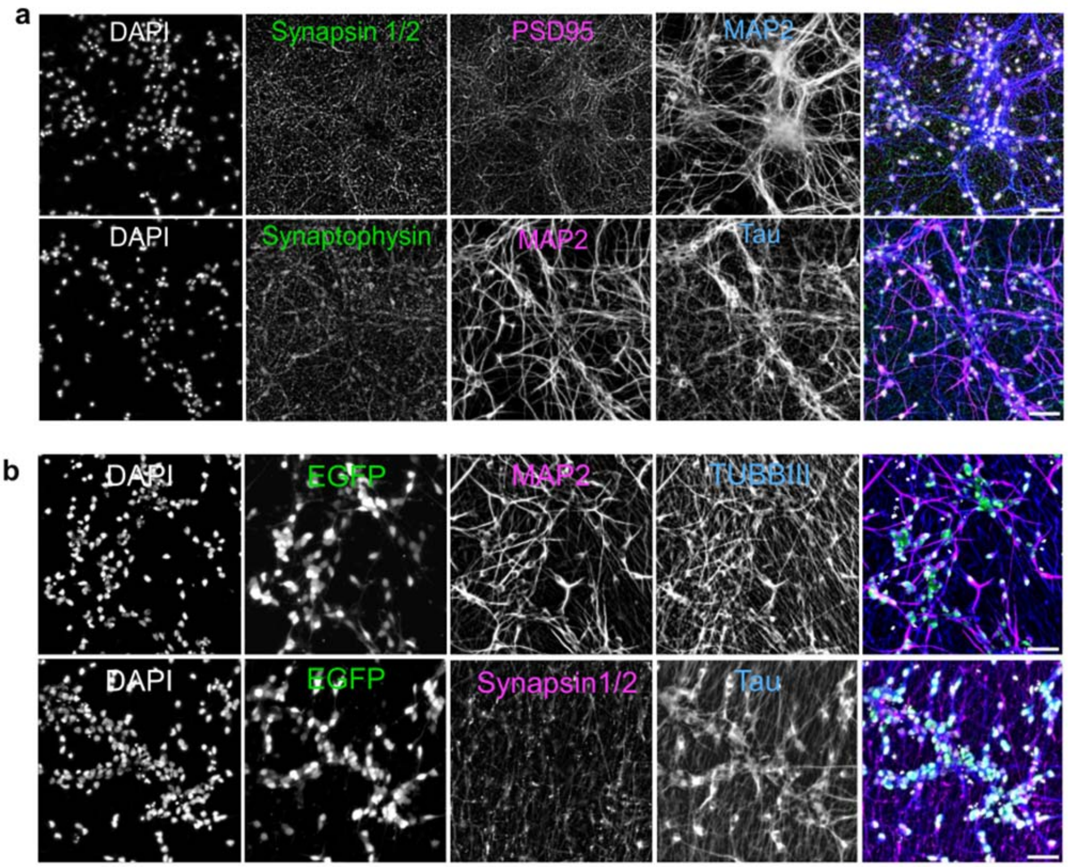


***Reviewer Figure 12*** *(new Appendix Figure S11a, b)****. a)*** *Representative images of primary cortical mouse neurons at DIV10 stained for markers of neuronal maturity including MAP2 and Tau as well as synaptic markers Synapsin1/2, Synaptophysin, and PSD95. Nuclei are indicated by DAPI staining. Scale bars*

*correspond to 50 µm.* ***b)*** *Representative images of iNeurons induced from human iPSCs at DIV13 after induction. The EGFP staining proves the efficient lentiviral delivery of NGN2-EGFP used for neuronal induction. Human iNeurons were stained for markers of neuronal maturity including MAP2, TUBIII and Tau as well as the synaptic marker Synapsin1/2. Nuclei are indicated by DAPI staining. Scale bars correspond to 50 µm.*

11. Discussion:
Typo 'Systematic' instead of 'systemic'

**Response and action:** We have adopted “systematic” instead of systemic in the discussion.

12. Methods:
a. RNA-seq computational data processing:
Does 'library size normalized gene expression levels' as DESeq2 input mean TPM here? Using unnormalized counts would be the correct input.

**Response:** The input for DESeq2 is indeed raw counts that get normalized during the pipeline using library size factors. We have amended this typo in the main text.

**Action:** Updates in methods: *“For this reason, we processed raw counts for these samples using DESeq2, and compared gene expression levels of BDNF, KCl at any time point versus all control samples, estimating in each case log2 fold changes, standard errors, and significance using the Wald test implementation (two-sided).”*

b. 'We compared the enrichment of gene ontology terms (...), using all mapped genes as genome background.' Does this mean that if a single read maps to a gene, this gene would be considered expressed? A more stringent filter needs to be applied to determine the background gene set. It could either be genes transcribed > 1 TPM in at least 1 replicate, or considered expressed by DESeq2.

**Response:** We thank the reviewer for this comment. Indeed, we applied the filter considered expressed by DESeq2 since the GO analysis tests the foreground (all genes in a specific cluster) against a background consisting of genes in all the other clusters (i.e. they have to be differentially expressed). The genes used for DESeq2’s statistical test are defined based on the general steps from DESeq2 i.e. *detected in at least two samples*, with at least one read in each, however to reach statistical significance (and thus be included in the background) they are expressed higher. In fact, only 90 genes are expressed at less than 100 counts. We have updated the description of the methods for GO analysis to clarify this point.

**Action:** Updates in methods **(section “RNA-seq computational data processing”)**: *“We compared the enrichment of gene ontology terms in each cluster versus other clusters using topGO version 2.42.0, using all genes belonging to other clusters ontology as genome background. 4,910 of these genes have a coverage of 100 reads or more in at least one sample.”*

c. Genomic data co-variation and loop data analysis:
'We defined Distal Regulatory Elements (DREs) as regions less than 2kbp and no further than 50Kbp from TSS regions.' Possibly a typo, and the authors mean 'more than 2 kbp'.

**Response and action:** We have updated this typo and amended the point highlighted by the reviewer, “more than 2 kbp” instead of “less”.

# Reviewer #3:

Ibarra et al., carried out temporal ATAC-seq and RNA-seq profiling in cultured neurons upon either BDNF or KCl (depolarization) treatments, and identified BDNF- vs. KCl-dependent differentially accessible chromatin regions and gene expression programs. To support their findings, the authors also carried out proteomics and CRISPR-mediated genome editing of the Arc enhancer. In addition, they analyzed correlations between activity dependent chromatin accessibility and human neuronal disease traits including a subset of neuropsychiatric disorders.

Studying the time-course of differential chromatin accessibility and transcriptional changes of BDNF- vs. KCl-treated neurons definetely provide interesting new information and datasets. However, this work needs significant revision before it can be considered for publication.

Major comments:
1. Comparative analysis of chromatin accessibility induced by distinct types of neuronal stimulation is definetely of interest and should be presented as the main focus of this work. On the other hand, my main criticism concerns the general conclusions about the BDNF- vs. KCl-induced chromatin regulations. It is unclear to which extent chromatin profiling from an in vitro culture system has general value regarding BDNF- vs. KCl(depolarization)-induced transcriptional programs in vivo. Indeed, distinct KCl-treatment protocols already differentially affect activity-response gene regulation (e.g. varying the concentration of KCl, or brief vs. sustained KCl treatment; PMID: 29681534; PMID: 28634074). Moreover, selection of activity-response genes is highly dependent on the cellular context (e.g. discussed in PMID 30359600). In this respect, the Discussion is rather brief and superficial and can be improved by taking into account these considerations.

**Response:** We appreciate this insightful feedback. We have strengthened the Discussion as the reviewer suggested.

**Action:** Updates in the main text (**Discussion, 2nd and 3rd paragraphs**):
“*Transcriptional and chromatin profiling in brain neurons upon stimulation has been a challenging task given the complexity of the brain, which hinders the detection of specific signals due to high variability across cell types (Winick-Ng et al, 2021). Our chromatin profiling in vitro culture system is presented as a tool to explore and identify candidate genomic regulatory elements limited to specific stimulation and cell type. We have validated our findings*

*using CRISPR perturbations of regulatory elements on transcription and compared chromatin responses to BDNF in mouse and human in an attempt to relate our findings to non-coding variants of human diseases. Nevertheless, we note that the effects on chromatin in response to stimulation are extremely specific to many parameters, such as stimulation strength and duration (Tyssowski et al, 2018; Fukuchi et al, 2017; Joo et al, 2016), neuronal type and connectivity (Fuentes-Ramos et al, 2021; Harabula & Pombo, 2021) and possibly cellular memory to previous stimuli (Yap & Greenberg, 2018).*

*The proper response to BDNF stimulation can be crucial during neurodevelopment when the transcriptional program must be tightly regulated to avoid untimely consequences (Cohen-Cory et al. 2010). On the other hand, neuronal activation by KCl prompts rapid functional responses that may require a fast activation of gene expression programs. This difference in the biological relevance between KCl activation and BDNF stimulation can be reflected in the distinct chromatin responses described here. At the chromatin accessibility level, we found that BDNF stimulation-induced comprehensive changes in the enhancer landscape at an early stage, with concomitant gene expression changes. This regulatory network involving enhancer-promoter interactions with the recruitment of different TFs to regulate transcription indicates a tight chromatin control. Conversely, KCl activation resulted in delayed chromatin remodeling of a similar set of enhancers. At an early time-point, the KCl-dependent changes in chromatin accessibility in promoters showed little correlation with transcription, albeit the transcriptional response was greater compared to BDNF. This could be related to the independence of cis-chromatin elements in regulating gene transcription at an early time-point of KCl or more complex TF dynamics at promoters involving TFs functioning as both activators and repressors (e.g., KLFs, E2Fs).”*

2. To validate the cooperativity of bZIP and EGRs in Arc enhancer regulation, the authors should delete the bZIP motif in ESCs as well (Fig. 4c,d, Fig S6b).

**Response:** As the reviewer suggested, we validated the cooperativity of bZIP and EGR in Arc enhancer regulation. Instead of generating new KO lines, we employed the small molecule inhibitor T5224, which prevents Fos and other members of the AP-1 family from binding DNA(Aikawa *et al*, 2008). The inhibitor treatment enabled us to directly test the cooperativity of bZIP and EGR using the control and KO mESC clones we have, bearing deletions in the distal genomic region (e.g., EGR motif) of the Arc gene. To this end, we generated neurons from the control and KO mESC clones, treated them with 1h of BDNF and increasing concentrations of T5224, and assessed Arc gene expression. The inhibitor treatment reduced *Arc* expression triggered by BDNF stimulation in a dose-dependent manner (**Reviewer Figure 13**, new Figure 4e) as well as other BDNF-inducible genes regulated by AP-1 family members (**Reviewer Figure 14**, new Appendix Figure S7d). Notably, the inhibitor treatment yielded significantly less Arc gene expression in the KO lines than in the control lines (**Reviewer Figure 13**, new Figure 4e). Also, when we tested the complex model considering the interaction between bZIP and EGR: Arc ~ EGR + bZIP + EGR:bZIP, it described our data significantly better than the simple model (Arc ~ EGR + bZIP). These results support the cooperativity of bZIP and EGR TFs in Arc

gene activation upon BDNF stimulation. As the KO clones have a deletion at the EGR motif of the Arc gene but not other motifs, we did not observe this complex model fitting with the other gene expression.

**Action:** Updates in the main text: *"Furthermore, we investigated the cooperativity between the transcription factors EGR and bZIP in the regulation of Arc expression. For this, we used the small molecule inhibitor T5224, which prevents AP-1(bZIP) family members, such as Fos, from binding DNA(Aikawa et al, 2008). The control and KO mESC clones bearing deletions in the distal genomic region of the Arc gene were differentiated into neurons and treated with BDNF for 1h and increasing concentrations of T5224. As expected, the inhibitor treatment reduced Arc expression triggered by BDNF stimulation (**Fig. 4e**) and also other BDNF-inducible genes such as Fos and Btg2 (**Appendix Figure S7d**), known to be transcriptionally regulated by AP-1 family members(Pagin et al, 2021). The inhibitor treatment yielded a significantly reduced level of Arc gene expression in the KO lines compared to the control lines, suggesting the joint effect of AP-1(bZIP) and EGR TFs on Arc expression. We tested two models to describe Arc expression. A simple model considered Arc expression to be additively dependent on the EGR binding motif (deletion of the genomic region containing EGR motifs) and bZIP (T5224 inhibitor concentration) as in Arc ~ EGR + bZIP. A more complex model included the interaction between bZIP and EGR: Arc ~ EGR + bZIP + EGR:bZIP. We found that the complex model could describe our data significantly better than the simple model (Likelihood Ratio Test $\chi^2(1)$ = 4.3438, P = 0.03714). As a negative control, we analyzed the BDNF-dependent expression of Fos and Btg2 in control and KO lines in the presence of T5224 (**Appendix Figure S7d**). The expression of Fos and Btg2 genes is better explained by a simple model relying solely on the bZIP term and not by the complex model including the interaction of two factors (Fos: Likelihood Ratio Test, $\chi^2(1)$ = 2.8148, P = 0.2448; Btg2: Likelihood Ratio Test $\chi^2(1)$ = 4.476, P = 0.1067). Altogether, our results validate the cooperativity of bZIP and EGR TF motifs at Arc enhancer in Arc gene activation upon BDNF stimulation."*

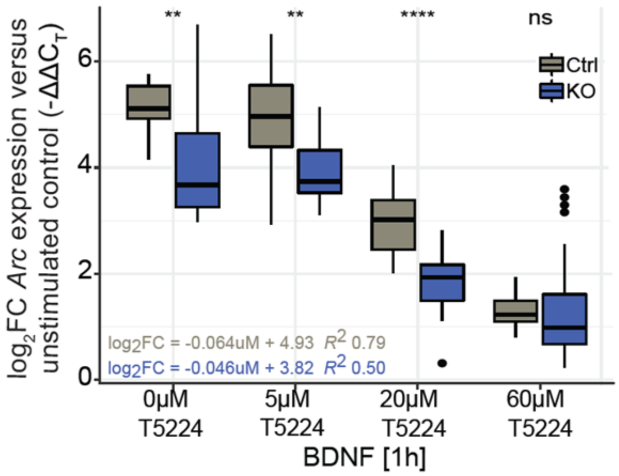


***Reviewer Figure 13** (new Figure 4e). qPCR-based measurement of Arc expression changes in control and Arc enhancer KO lines after 1h of BDNF stimulation in the presence of varying concentrations of T5224. Normalized to RPL-13 (housekeeping gene) and unstimulated cells without the inhibitor (N=4, 2 independent biological replicates of 2 control and 2 KO lines (B1, E11), measured in triplicates)*

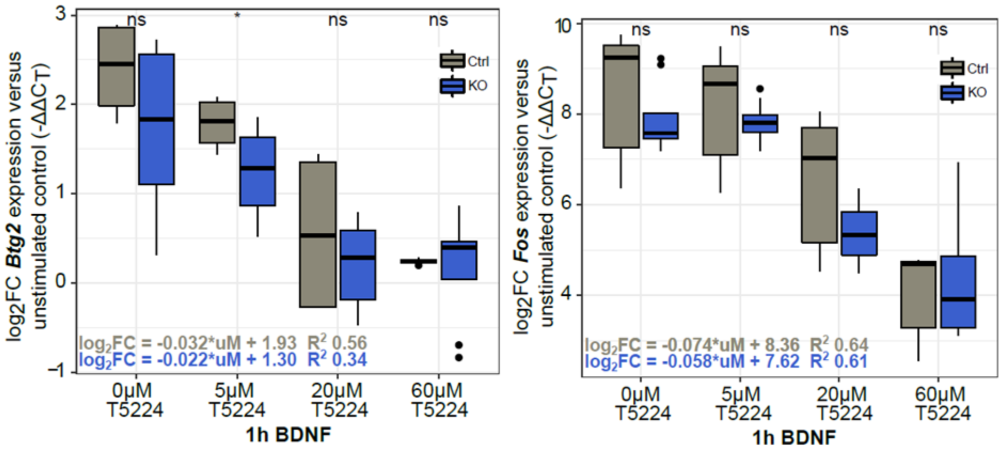


***Reviewer Figure 14*** *(new Appendix Figure S7d)**.** qPCR measurement of Btg2 (left) and Fos (right) expression in control and Arc enhancer KO lines after 1h of BDNF stimulation in the presence of varying concentrations of T5224. Normalized to RPL-13 (housekeeping gene) and unstimulated cells without the inhibitor (biological duplicates of 2 control and 2 KO lines (B1, E11), measured in triplicates)*

3. Fos and Egr ChIP-seq should be carried out in cells treated with the different conditions to validate the differentially accessible (DA) binding sites identified in the study.

**Response and action:** We thank the reviewer for this suggestion. We attempted several times to carry out Fos ChIP-seq but failed enriching Fos-bound chromatin regions in particular after sequencing library generation, which could be due to the signal vs. noise ratio of the tested antibodies. We did not observe this issue with the Egr1 ChIP-seq library. To this end, we focused on our identified BDNF-specific Arc enhancer, which is differentially accessible upon BDNF stimulation and shows binding sites for Fos and Egr1 transcription factors. Using ChIP-qPCR with anti-c-Fos antibody and anti-Egr1 antibody, we validated the binding of these factors on the TSS of the Arc gene and different positions across the Arc enhancer. Overall, Fos and Egr1 protein binding to these regions increased after 1h of BDNF treatment compared to control and KCl treatment (**Reviewer Figure 15**, new Appendix Figure S8).

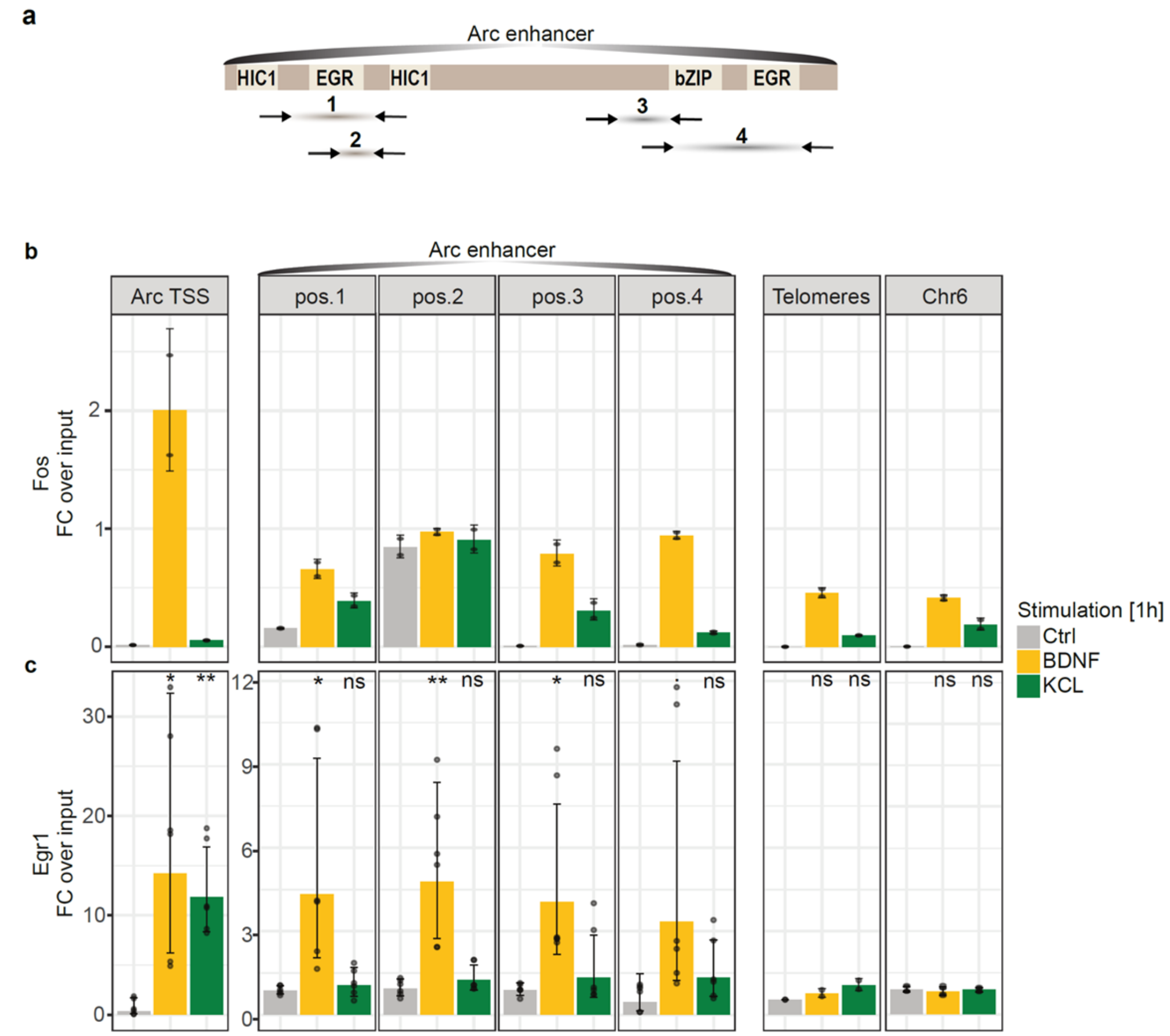


***Reviewer Figure 15*** *(new Appendix Figure S8). Validation of Fos and Egr1 binding to Arc enhancer at 1h after stimulation. (**a**) Annealing positions of primer sets designed to identify binding on the putative Arc enhancer, specific to BDNF stimulation. (**b**) Fos and (**c**) Egr1 enrichment on Arc TSS and Arc enhancer identified by ChIP-qPCR. Negative control loci are telomeres and a gene-desert area in chromosome 6. Fold change over input is calculated as 2^-dCt sample to input (N=3 for Egr1; N=1 for Fos; error bars as s.d.;. P<0.1, *P<0.05,**P<0.01, ns: not significant, two-sided t-test with control as the reference group)*

**Action:** Updates in the main text: "*This distal region exhibited properties of an active enhancer (enriched H3K27ac and H3K4me1 marks obtained from neuronal epigenomics data)(Malik et al, 2014), bound CTCF(Sams et al, 2016; Ren et al, 2017) and had a high Hi-C contact score with the Arc gene, as measured by shaman package(Bonev et al, 2017). The binding of Egr1 and a bZIP family protein, Fos to this region was also examined by ChIP-qPCR (**Appendix Figure S8**).*"

4. While Fos mRNA levels appear higher for KCl- than BDNF-treated neurons at 1 hour (Fig. 1d, 3c), proteomics data shows the opposite. The authors did not provide a convincing explanation for this.

**Response:** We thank the reviewer for this comment. The discordance between Fos mRNA levels and protein levels could be due to their multi-layered regulations - both *fos* mRNA and Fos protein have a very short half-life and a high turnover rate, suggesting a complex transcriptional and posttranscriptional regulation before, during, and after stimulation. Numerous studies showed that Fos protein exerts negative autoregulation on the *fos* gene(Schönthal *et al*, 1989; Wilson & Treisman, 1988; Rahmsdorf *et al*, 1987). Fos C-terminal domain phosphorylated by ERK stabilized Fos protein (Chen *et al*, 1993; Gilley *et al*, 2009; Monje *et al*, 2003) and downregulated *fos* transcription(Wilson & Treisman, 1988). Fos heterodimerization with Jun allowed them to bind *cis*-regulatory element AP-1(Rauscher *et al*, 1988b, 1988a) and in turn downregulated *fos* expression(König *et al*, 1989; Schönthal *et al*, 1989). However, Fos protein degradation mediated by the proteasome-ubiquitin system was also dependent on its partner Jun(Tsurumi *et al*, 1995), indicating the complex equilibrium between stabilization and degradation.
Taken together, the cis- and trans-regulatory features activated differentially by BDNF stimulation or KCl depolarization may affect Fos protein and mRNA stability. BDNF stimulation seems to enhance Fos protein stability and detriment the mRNA levels, maybe by reducing the transcription or accelerating RNA decay in consequence of differential phosphorylation patterns of the C-terminus in BDNF- versus KCl-stimulated cells.

**Action:** Updates in the main text (**last paragraph section TF motifs underlying chromatin responses**): *"Furthermore, while Fos protein was more abundant after 1h of BDNF treatment than in KCl, Fos mRNA showed the opposite trend, with higher levels in KCl-treated neurons. This discordance might be due to the complex equation of Fos protein and mRNA levels. For instance, there is negative autoregulation of the fos gene upon Fos protein (Rahmsdorf et al, 1987; Schönthal et al, 1989; Wilson & Treisman, 1988), where a higher stabilization of the Fos protein via phosphorylation(Chen et al, 1993; Gilley et al, 2009; Monje et al, 2003) results in lower levels of fos mRNA(Rauscher et al, 1988a, 1988b)."*

Minor comments:
1. Fig. 1d, heatmaps. If I understand correctly, the authors show Z-scores of fold change induction. If this is the case, I cannot distinguish whether each gene is up- or down-regulated as compared with non-treated control neurons. If they want to use Z-scores for heat maps, they should visualize normalized read numbers (e.g. cpm) of both the treated and non-treated (control) samples.

**Response:** We thank the reviewer for this suggestion. In the revised manuscript, we updated Figure 1d heatmaps by using normalized counts instead of log2 fold changes main heatmap, also including the control samples and row-scaling values by rows. We hope that this allows a direct visual comparison between treated and non-treated control samples.

**Action:** We updated the **Figure 1d** heatmap panel and caption to highlight the usage of normalized counts.

2. A few missing citations of relevant studies: PMID: 26595656, PMID:19116276, PMID:19193899

We included the following citations in the Introduction and results section, next to currently written in our manuscript that relate to them.

***Updates in the main text.***

- *Introduction: "The TFs transcriptionally activated by KCl include neuronal PAS domain protein 4 (NPAS4), FOS, and EGRs, many of which overlap with BDNF stimulation(Joo et al, 2016; Kawashima et al, 2009; Pintchovski et al, 2009)"*
- *Results: We focused on the Arc gene, a key effector for synaptic function*

3. Neuronal activity controls BDNF expression, and neuronal activity-dependent BDNF expression triggers synaptic plasticity and other changes in neurons. On the other hand, it is misleading to say: 'BDNF-induced neuronal activity' as e.g. mentioned in the title.

**Response and action**: We thank the reviewer for pointing this out. The statement 'BDNF-induced neuronal activity' could be misleading because neuronal activity often refers to electrical activity within synapses, as reviewer #1 (minor comment 4) has also been pointed out.

Here we intended to refer to BDNF-induced neuronal activation, such as BDNF-induced TrkB receptor activation and increased signaling pathway in neurons. We are changing the title to 'Comparative chromatin accessibility upon BDNF stimulation delineates neuronal regulatory elements' and will use the term *neuronal stimulation* instead of neuronal activity (reviewer #1 minor comment).

## References

Aikawa Y, Morimoto K, Yamamoto T, Chaki H, Hashiramoto A, Narita H, Hirono S & Shiozawa S (2008) Treatment of arthritis with a selective inhibitor of c-Fos/activator protein-1. *Nat Biotechnol* 26: 817–823

Alder J, Thakker-Varia S, Bangasser DA, Kuroiwa M, Plummer MR, Shors TJ & Black IB (2003) Brain-derived neurotrophic factor-induced gene expression reveals novel actions of VGF in hippocampal synaptic plasticity. *J Neurosci* 23: 10800–10808

Bonev B, Mendelson Cohen N, Szabo Q, Fritsch L, Papadopoulos GL, Lubling Y, Xu X, Lv X, Hugnot J-P, Tanay A, *et al* (2017) Multiscale 3D Genome Rewiring during Mouse Neural Development. *Cell* 171: 557–572.e24

Chen RH, Abate C & Blenis J (1993) Phosphorylation of the c-Fos transrepression domain by mitogen-activated protein kinase and 90-kDa ribosomal S6 kinase. *Proc Natl Acad Sci U S A* 90: 10952–10956

Gilley R, March HN & Cook SJ (2009) ERK1/2, but not ERK5, is necessary and sufficient for phosphorylation and activation of c-Fos. *Cell Signal* 21: 969–977

Guzowski JF, McNaughton BL, Barnes CA & Worley PF (1999) Environment-specific expression of the immediate-early gene Arc in hippocampal neuronal ensembles. *Nat Neurosci* 2: 1120–1124

Joo J-Y, Schaukowitch K, Farbiak L, Kilaru G & Kim T-K (2016) Stimulus-specific combinatorial functionality of neuronal c-fos enhancers. *Nat Neurosci* 19: 75–83

Kawashima T, Okuno H, Nonaka M, Adachi-Morishima A, Kyo N, Okamura M, Takemoto-Kimura S, Worley PF & Bito H (2009) Synaptic activity-responsive element in the Arc/Arg3.1 promoter essential for synapse-to-nucleus signaling in activated neurons. *Proc Natl Acad Sci U S A* 106: 316–321

König H, Ponta H, Rahmsdorf U, Büscher M, Schönthal A, Rahmsdorf HJ & Herrlich P (1989) Autoregulation of fos: the dyad symmetry element as the major target of repression. *EMBO J* 8: 2559–2566

Liu J, Amar F, Corona C, So RWL, Andrews SJ, Nagy PL, Shelanski ML & Greene LA (2018) Brain-Derived Neurotrophic Factor Elevates Activating Transcription Factor 4 (ATF4) in Neurons and Promotes ATF4-Dependent Induction of Sesn2. *Front Mol Neurosci* 11: 62

Malik AN, Vierbuchen T, Hemberg M, Rubin AA, Ling E, Couch CH, Stroud H, Spiegel I, Farh KK-H, Harmin DA, *et al* (2014) Genome-wide identification and characterization of functional neuronal activity–dependent enhancers. *Nat Neurosci* 17: 1330–1339

Monje P, Marinissen MJ & Gutkind JS (2003) Phosphorylation of the carboxyl-terminal transactivation domain of c-Fos by extracellular signal-regulated kinase mediates the transcriptional activation of AP-1 and cellular transformation induced by platelet-derived growth factor. *Mol Cell Biol* 23: 7030–7043

Pagin M, Pernebrink M, Pitasi M, Malighetti F, Ngan C-Y, Ottolenghi S, Pavesi G, Cantù C & Nicolis SK (2021) FOS Rescues Neuronal Differentiation of Sox2-Deleted Neural Stem

Cells by Genome-Wide Regulation of Common SOX2 and AP1(FOS-JUN) Target Genes. *Cells* 10

Pintchovski SA, Peebles CL, Kim HJ, Verdin E & Finkbeiner S (2009) The serum response factor and a putative novel transcription factor regulate expression of the immediate-early gene Arc/Arg3.1 in neurons. *J Neurosci* 29: 1525–1537

Rahmsdorf HJ, Schönthal A, Angel P, Litfin M, Rüther U & Herrlich P (1987) Posttranscriptional regulation of c-fos mRNA expression. *Nucleic Acids Res* 15: 1643–1659

Rauscher FJ 3rd, Cohen DR, Curran T, Bos TJ, Vogt PK, Bohmann D, Tjian R & Franza BR Jr (1988a) Fos-associated protein p39 is the product of the jun proto-oncogene. *Science* 240: 1010–1016

Rauscher FJ 3rd, Voulalas PJ, Franza BR Jr & Curran T (1988b) Fos and Jun bind cooperatively to the AP-1 site: reconstitution in vitro. *Genes Dev* 2: 1687–1699

Ren G, Jin W, Cui K, Rodrigez J, Hu G, Zhang Z, Larson DR & Zhao K (2017) CTCF-Mediated Enhancer-Promoter Interaction Is a Critical Regulator of Cell-to-Cell Variation of Gene Expression. *Mol Cell* 67: 1049–1058.e6

Ruiz-Velasco M, Kumar M, Lai MC, Bhat P, Solis-Pinson AB, Reyes A, Kleinsorg S, Noh K-M, Gibson TJ & Zaugg JB (2017) CTCF-Mediated Chromatin Loops between Promoter and Gene Body Regulate Alternative Splicing across Individuals. *Cell Syst* 5: 628–637.e6

Sams DS, Nardone S, Getselter D, Raz D, Tal M, Rayi PR, Kaphzan H, Hakim O & Elliott E (2016) Neuronal CTCF Is Necessary for Basal and Experience-Dependent Gene Regulation, Memory Formation, and Genomic Structure of BDNF and Arc. *Cell Rep* 17: 2418–2430

Schönthal A, Büscher M, Angel P, Rahmsdorf HJ, Ponta H, Hattori K, Chiu R, Karin M & Herrlich P (1989) The Fos and Jun/AP-1 proteins are involved in the downregulation of Fos transcription. *Oncogene* 4: 629–636

Tsurumi C, Ishida N, Tamura T, Kakizuka A, Nishida E, Okumura E, Kishimoto T, Inagaki M, Okazaki K & Sagata N (1995) Degradation of c-Fos by the 26S proteasome is accelerated by c-Jun and multiple protein kinases. *Mol Cell Biol* 15: 5682–5687

Tuvikene J, Pruunsild P, Orav E, Esvald EE & Timmusk T (2016) AP-1 transcription factors mediate BDNF-positive feedback loop in cortical neurons. *Journal of Neuroscience* 36: 1290–1305

Tyssowski KM, DeStefino NR, Cho JH, Dunn CJ, Poston RG, Carty CE, Jones RD, Chang SM, Romeo P, Wurzelmann MK, *et al* (2018) Different Neuronal Activity Patterns Induce Different Gene Expression Programs. *Neuron* 98: 530–546.e11

Wilson T & Treisman R (1988) Fos C-terminal mutations block down-regulation of c-fos transcription following serum stimulation. *EMBO J* 7: 4193–4202

Yap E-L & Greenberg ME (2018) Activity-Regulated Transcription: Bridging the Gap between Neural Activity and Behavior. *Neuron* 100: 330–348

RE: MSB-2021-10473R, Comparative chromatin accessibility upon BDNF stimulation delineates neuronal regulatory elements

Thank you for sending us your revised manuscript. We have now heard back from the two reviewers who were asked to evaluate your revised study. As you will see below, they think that the study has improved as a result of the performed revisions. They do however still list a few remaining concerns, which we would ask you to address in another round of revision.

We would also ask you to address some editorial issues listed below.

Reviewer #2:

The authors have made extensive efforts to provide additional experimental data and computational analyses. The revised manuscript is of great relevance to the field and most of the technical concerns have been successfully addressed. We remain critical about the stringency used for the false discovery rate threshold and advise the authors to perform additional quality controls prior to publication.

Comments

Regarding major point 3: We remain concerned about the number of potential false positive discoveries due to the chosen FDR threshold of 0.1 for identification of differentially expressed genes (DEGs) and differentially accessible regions (DARs). There is a striking difference in the numbers of identified genes/regions between the different FDR thresholds, especially for the ATAC-Seq data. The authors have to clarify whether the reported GO terms obtained using a FDR of 0.1 are consistent/similar with terms obtained when an FDR of 0.05 / 0.01 is applied. The TF motif discovery identified in association with DARs should also be investigated at FDR of 0.05 / 0.01. The FDR/GO terms/number of DARs reported in the text should be those from lowest FDR.

Regarding minor point 5: The references provided in response to our comment do not contain the term 'biphasic'. The authors should avoid the use of the term 'biphasic', as it is not reasonable to deduce biphasic response when only taking measurements at three different timepoints (i.e. "it resembles a biphasic response for the timepoints studied"). The authors should avoid over-interpreting the results.

Regarding minor point 6: The observation that there is an increase in correlation of loop-associated peak pairs during cortical neurodevelopment, in contrast to embryonic stem cells is interesting. Beagan et al., 2020 showed that there are both pre-existing and de novo loops formed in response to neuronal activation. The manuscript should indicate the percentage of DARs/ DEGs that are in preformed loops in the unstimulted cortical neurons, and the number of DARs/DEGs that are not connected via loops in the unstimulated neurons. Do these groups have distinct biological functions? We encourage the authors to put the current results in a broader perspective to bring more value to this part of the study.

Regarding minor point 7: We appreciate that the authors included more details in their methods section and also provided the notebooks used for the analysis. However, the details provided still do not clearly explain how the Z-scores were calculated from the correlation. We recommend either including a few additional sentences in the methods section to explain this point explicitly or providing more documentation in the notebooks.

Reviewer #3:

The authors addressed most of my points of criticism and the manuscript has been improved. However, I still have a few final concerns that require revision before publication.

Specific points:
1. I have a concern regarding new Figure 4d,e and Appendix Figure S7c. According to Figure S7c, variation of Arc inducibility in control clones is quite big, raising a concern regarding authors' claim that enhancer KO reduces Arc inducibility. Indeed, the median inducibility of two KO clones (C5, E11) is higher than wild-type R1 clone. I recommend to move Figure 4d,e to supplementary materials and tone down related conclusions in the abstract and main text.
2. The authors added the sentence: 'Deleting these TF motifs did not show any effect on Arc gene expression upon KCl stimulation (Fig. 4d)' in the new manuscript. However, Arc is not induced even in control in vitro neuronal differentiated ESCs.
3. Related to this, even though the authors showed that Arc induction by KCl treatment is modest as compared with Bdnf treatment in ex vivo cultured cortical neurons (Figure 1), the complete lack of Arc induction by KCl treatment in neuronal differentiated ESCs is unclear. This raises a concern whether KCl-mediated depolarization can indeed provide a sufficient stimulus to induce downstream genes in neuronal differentiated ESCs. Because KCl experiments in Figure 4d do not provide essential additional information, these data may be removed from the panel.
4. There is a possibility that KCl treatment may cause Bdnf release, and Bdnf may also affect neuronal excitability. In the Discussion section, the authors should discuss about this potential confound.
5. I think that information about Fos and Egr1 antibodies is missing.

Reviewer #2:

The authors have made extensive efforts to provide additional experimental data and computational analyses. The revised manuscript is of great relevance to the field and most of the technical concerns have been successfully addressed. We remain critical about the stringency used for the false discovery rate threshold and advise the authors to perform additional quality controls prior to publication.

Comments

**Regarding major point 3:** We remain concerned about the number of potential false positive discoveries due to the chosen FDR threshold of 0.1 for identification of differentially expressed genes (DEGs) and differentially accessible regions (DARs). There is a striking difference in the numbers of identified genes/regions between the different FDR thresholds, especially for the ATAC-Seq data. The authors have to clarify whether the reported GO terms obtained using a FDR of 0.1 are consistent/similar with terms obtained when an FDR of 0.05 / 0.01 is applied. The TF motif discovery identified in association with DARs should also be investigated at FDR of 0.05 / 0.01. The FDR/GO terms/number of DARs reported in the text should be those from lowest FDR.

We thank the reviewer's concern about the FDR threshold. To address this, we collected DE-genes and DA-peaks using a FDR of 0.01 and performed a similar analysis to the ones previously reported. We provided the comparison results in a new **Appendix Figure S3** and clarified each point below.

**"The authors have to clarify whether the reported GO terms obtained using a FDR of 0.1 are consistent/similar with terms obtained when an FDR of 0.05 / 0.01 is applied."**

Indeed, the reported GO terms (Figure 1d) are consistent with terms obtained when FDR of 0.01 is applied. We want to emphasize that to perform the reported GO analysis (Figure 1d), we selected the top 5,000 genes with the lowest adjusted p-value. Of those 5,000 DE-genes, 4,910 had 100 counts or more, and 4,907 had an adjusted *P*-value of 0.01 or lower. We have updated this information in the figure caption of **Figure 1d**, **Appendix Figure S3** and in the main text (purple/underlined):

"To identify BDNF-induced genes, we performed unsupervised clustering of the top 5,000 significant DE-genes across stimuli and time-points, obtaining early (1h) and late (6/10h) clusters across stimuli (**Fig. 1d**). Of these 5000 DE-genes, 4910 had more than 100 counts and 4907 had an adjusted p-value of 0.01 or less."

We show almost equivalent GO terms between the currently used top 5,000 genes with the lowest adjusted p-value and 4,907 genes selected using FDR of 0.01 and more than 100 counts (**top, Reviewer Figure 16**). Global comparison of log2 fold changes between two groups indicates significant correlations (**bottom, Reviewer Figure 16**). Top ontologies are globally maintained by clusters (**Reviewer Figure 17**), and fluctuations are associated with the internal steps of topGO to generate background genes, due to a slightly modified version of the original gene sets. Hence, log2 odds ratios are comparable and equivalent to the previous ones, for matched top-terms shown in each cluster (**Figure 1e**).

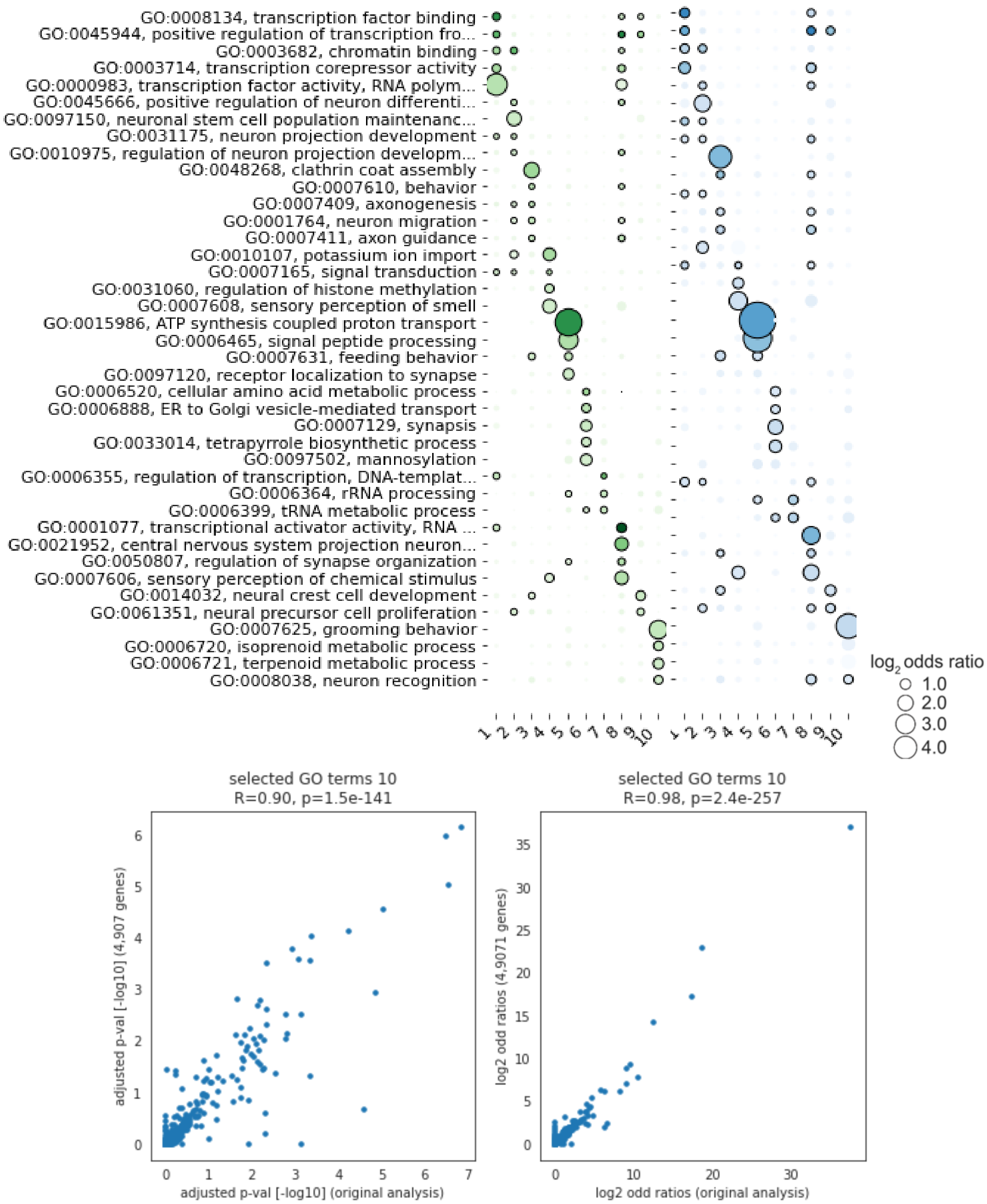


***Reviewer Figure 16** (related to Figure 1d)*:

***(top)** Comparison between adjusted p-values and log2 odds ratios when using all 5,000 genes (green) and a subset of 4,907 genes selected using FDR=1% and more than 100 counts (blue), in a new topGO run adapted from the original scripts. The blue dotplot has been included in the main manuscript, as **Appendix S3c.***

***(bottom)** correlation between adjusted p-values and log2 odd ratios of matched terms. Notebook available [here](here).*

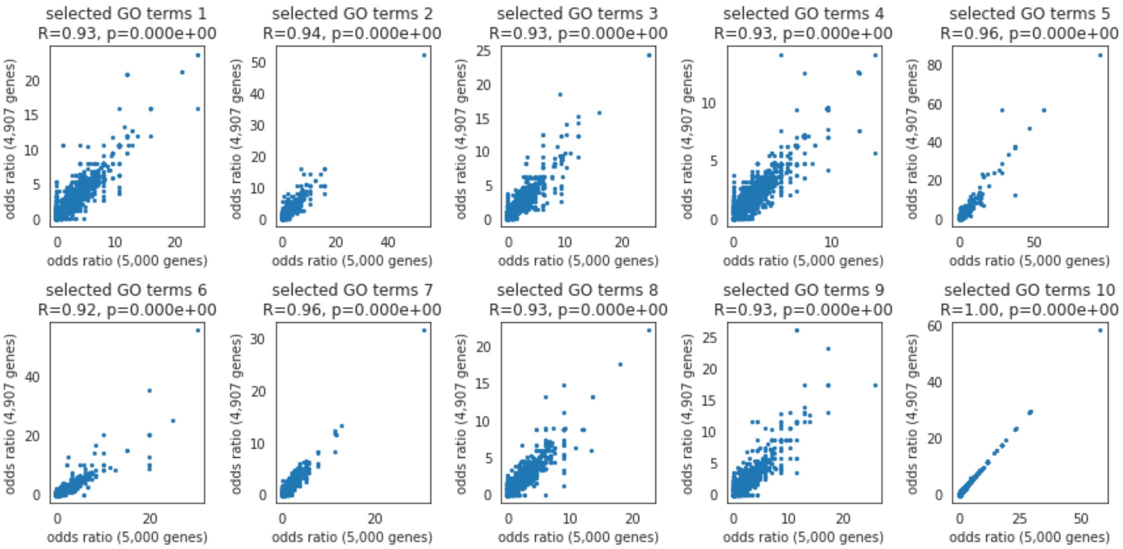


***Reviewer Figure 17*** *(related to Figure 1d)**:** Correlation between GO log2 odds ratios for cluster-wise GO analyses, when using genes with 100 counts and DE-genes with adjusted P-value < 0.01 in at least one condition (y-axes), and or P < 0.1 (x-axes). Titles indicate cluster number from **Figure 1d** and correlation coefficient for matched terms.*

**"…The TF motif discovery identified in association with DARs should also be investigated at FDR of 0.05 / 0.01…."**

We have recalculated the TF-motif enrichments using only DA-peaks with FDR of 0.01 or lower (Revision #1, **Reviewer Figure 4, now S3b**) and included them as a supplementary panel (**S3d**, also **Reviewer Figure 18**). Due to the reduced number of tested DA-peaks for some stimulation/time combinations, there is a lack of signal in some motif observations, which we associate with the power in the statistical test. This effect is more substantial for closing DA-peaks, where peaks are mostly reduced. Importantly, all reported pioneer (bZIP) and co-regulatory factors signals (CTCF/Hbox/EGR/HIC1) are still significant with the FDR of 0.01 threshold. We have updated the main text accordingly.

**Text update:**

- *"The majority of TF motifs reported next are still significantly detected when using DA-peaks selected with the FDR threshold of 1%, (Appendix Figure S3d)"*

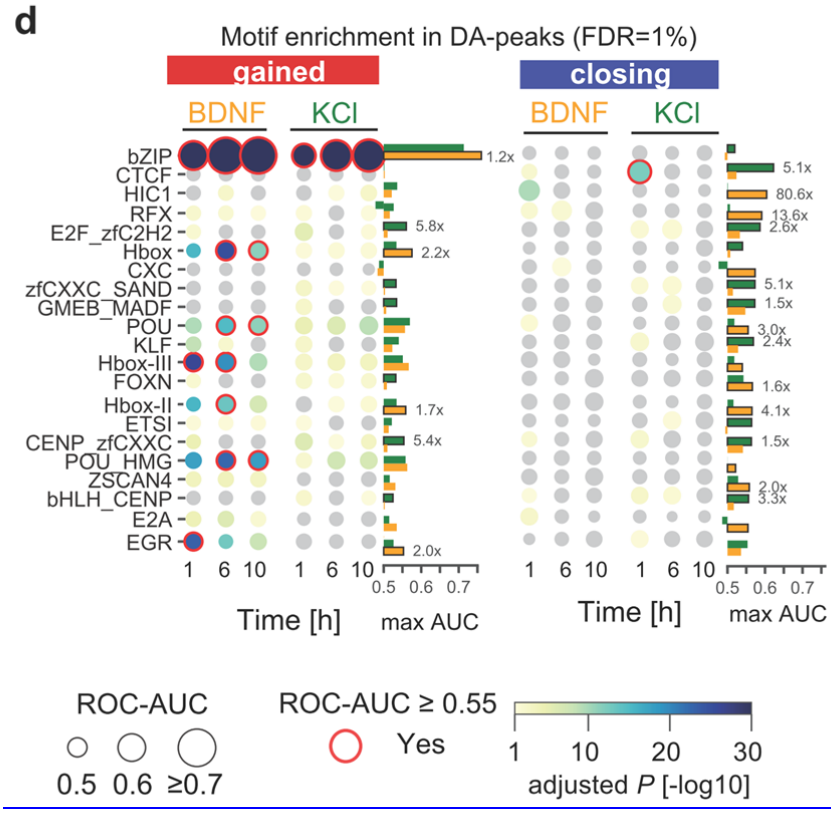


***Reviewer Figure 18(S3d)*** *(related to Figure 3b)**:** TF motif enrichment analyses using DA-peaks with FDR=1% and GENRE-negative regions for those. Color legends as in **Figure 3b**. Importantly, the main reported trends for pioneer (bZIP) and co-regulatory factors are maintained (CTCF, EGR, Hbox, HIC1). HIC1 has a significant adjusted p-value, but a ROC-AUC lower than 0.55, likely due to fewer sequences used for testing in closing DA-peaks for BDNF 1h.*

**"The FDR/GO terms/number of DARs reported in the text should be those from lowest FDR. "**

We agree with the reviewer that it is important to report the results from the lowest FDR, which we have now also mentioned in the main text. Yet we disagree about **replacing** the results in the **main** text and **figures** with the lowest FDR data. The reason for this is that we find the results overall being very similar across FDR thresholds while a small FDR additionally reduces power for some of the analyses. We are concerned that performing analyses with varying FDR could lead to confusion. Therefore, in the resubmitted version, we are reporting the visualization of the number of DE-genes, DA-peaks, and GO terms with FDR=1% as a supplementary panel (**Appendix Figure S3a-b**) and while we keep most analyses and interpretations using FDR=10% we now provide wherever possible a mention that the number of closing peaks is lower with FDR=1%.

The plots presented in this reply have been included in **Appendix Figure S3**, with the next caption

- ***"Appendix Figure S3 Effect of FDR 1% in DE-genes, DA-peaks and downstream analyses.***
*(**a**) number of DA-genes using FDR = 1%. Labels and colors as in **Figure 1c**.*
*(**b**) number of DA-peaks using FDR = 1%. Labels and colors as in **Figure 2b**.*

*(**c**) GO analysis using subset of 4,907 genes with FDR = 1% and more than 100 counts. Colors and legends as indicated in **Figure 1e**.*
*(**d**) TF motif enrichment analyses using DA-peaks with FDR=1% and GENRE-negative regions for those. Colors, legends as in **Figure 3b**."*

**Regarding minor point 5**: The references provided in response to our comment do not contain the term 'biphasic'. The authors should avoid the use of the term 'biphasic', as it is not reasonable to deduce biphasic response when only taking measurements at three different timepoints (i.e. "it resembles a biphasic response for the timepoints studied"). The authors should avoid over-interpreting the results.

We thank the reviewer for this suggestion. To minimize over-interpretation the term biphasic has been removed in the revised manuscript. The subhead of the first result part is updated to "BDNF triggers diverse transcriptional responses".

**Regarding minor point 6:** The observation that there is an increase in correlation of loop-associated peak pairs during cortical neurodevelopment, in contrast to embryonic stem cells is interesting. Beagan et al., 2020 showed that there are both pre-existing and de novo loops formed in response to neuronal activation. The manuscript should indicate the percentage of DARs/DEGs that are in preformed loops in the unstimulted cortical neurons, and the number of DARs/DEGs that are not connected via loops in the unstimulated neurons. Do these groups have distinct biological functions? We encourage the authors to put the current results in a broader perspective to bring more value to this part of the study.

Thank you for this thoughtful suggestion. In our analyses, we used the loops defined in the study by Bonev et al., 2017 which characterize loops formed during neurodevelopment and pre-established loops in cortical neurons (CN). We found associations between CN loops and variability of chromatin peaks and enrichment of DA-peak / DE-gene pairs changing in the same direction when being part of a loop (**Appendix Figure S4a-b**). We agree that additional analysis of loop-associated peak pairs would be insightful, and we have included in this resubmission **two additional analyses** (**Appendix Figure S4c-g**) to address this point (see below for details).
We also agree that the integrating analysis of loop-associated peak pairs with the data from Beagan et al., 2020 would be insightful. However, detailed analyses that go beyond the co-variation are non-trivial and would not alter the main conclusions from our study, we therefore consider any analysis, beyond what we have added, outside the scope of this manuscript. We have added a discussion point about the future studies investigating this point (new edits in purple/underlined):
"Altogether, following BDNF stimulation, changes in enhancer accessibility appear to translate into gene expression changes, and these correlate with changes in the physical connectivity in the genome observed during neurodevelopment and pre-established loops in cortical neurons. It will be interesting to investigate the relationship of DA-peaks and activity-dependent *de novo* loops in future studies to understand whether DA regions may play a role in restructuring chromatin loops upon BDNF stimulation."

**Specific changes:**
To define the extent of DAR and DEGs responsive to BDNF or KCl that are present in loops in ESC, NPC, or CN, we present additional panels in the manuscript (**Reviewer Figure 19, now Appendix Figure S4c-g)**:

- We iteratively counted the number of DE-genes with and without ES/NPC/CN loops from Bonev et al 2017 (**S4c**), DA-peaks with and without loops (**S4d**), and DA-peaks/DE-gene pairs with and without loops (**S4e**).
- From these numbers, we calculated the percentage of DEG, DAR, or DEG-DAR pairs linked to CN or ESC loops, specific to BDNF or KCL stimulations (1h timepoint):
    - *We observed higher percentages of DE-genes linked to cortical neuron (CN) loops in BDNF 1h (45.3%) and KCl 1h (39.7%) versus embryonic stem cell (ES) loops (26.7% and 23.4% for BDNF and KCl 1h, respectively) (Appendix Figure S4c). These percentages were also higher when comparing DA-peaks within loops in CN or ESC (9.2% for BDNF and 10.9% for KCl versus 4.9% and 5.2%, respectively). The number and percentage of DE-genes with DA-peaks linked via CN loops is higher for BDNF 1h (446, 20.3%) than KCl 1h (363, 10.1%) (Appendix Figure S4c)*
- Additionally, log2 odds ratios in **Appendix Figure S4e** highlight the relevance of BDNF-specific DEG and DAR pairs present in CN loops among other enrichments found. We provide this as a general analysis while the careful follow-up investigations remain for further work.
- We explored the association between the genes present in these loops and their biological functions using GO analysis, for the DE-genes with loops versus DE genes without loops, and DAR/DEG pairs with loops versus DEG without loops (row-wise). This comparison does not indicate a strong association between biological processes and DE-genes with DA-peaks and CN loops in BDNF 1h.
    - *The DE-genes or the DE-genes linked to DA-regions associated with loops did not show any enrichment in specific GO terms, as seen by GO analysis. Enrichment signals are weaker in comparison with GO analyses performed using broad dynamic gene clusters (Appendix Figure S4d).*

The notebook for this analysis, genes, and ontologies are available here notebook.

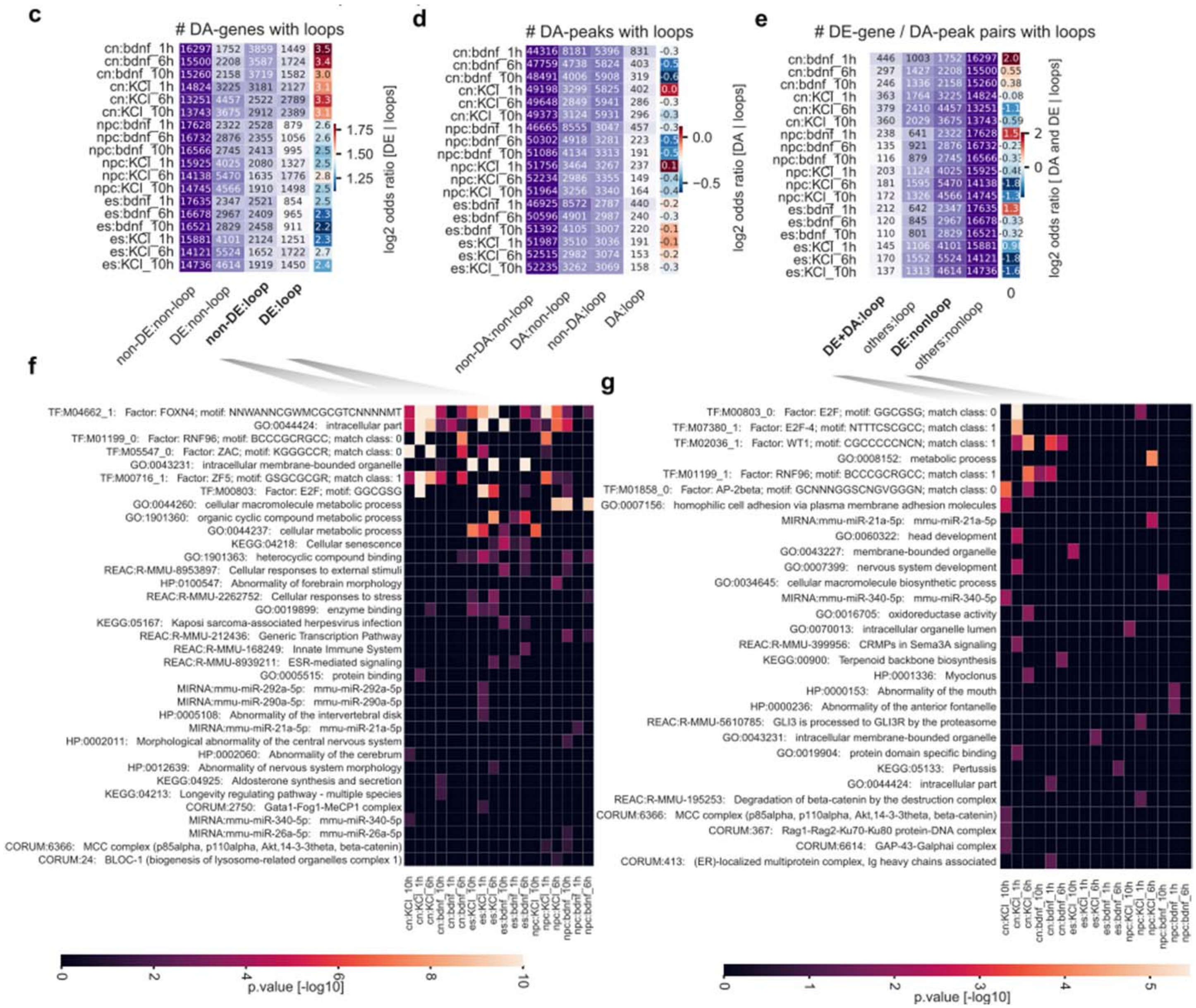


**Reviewer Figure 19 (**also new **Appendix Figure S4c-g**)

(**c**) Heatmap indicating the number of events where a DE-gene is included in a loop in cortical neurons (CN), neural progenitor cells (NPC), or embryonic stem cells (ES), for any of the DE-genes comparisons between BDNF and KCl versus control cells (purple). Right log2 odds ratio indicate the relationship (DE:loop / non-DE-loop) / (non-DE:loop / non-DE:non-loop)

(**d**) Similar to **c,** now indicating the number of DA-peaks with loops in any of the described categories. Log2 odd ratios indicate the relationship (DA-peak:loop / non-DA-peak:loop) / (non-DA-peak:loop / non-DA-peak:non-loop)

(**e**) Similar to **c-d,** now indicating the number of genes connected to peaks as DE-gene/DA-peak pairs, in any of the described categories. Log2 odd ratios indicate the relationship (DA+DE:loop / DE:loop) / (non-DA-peak:loop / non-DA-peak:non-loop)

(**f**) Top gene ontology results for a query of 1449 foreground (DE-genes with loops) and 3859 (non-DE genes with loops) background genes associated with BDNF 1h and CN loops. Colors indicate raw *P* values.

(**g**) Top gene ontology results for a query of 446 foregrounds (DE-gene/DA-peak pairs with loops) and 1752 (DE genes with loops) background genes associated with BDNF 1h and CN loops.

**Regarding minor point 7:** We appreciate that the authors included more details in their methods section and also provided the notebooks used for the analysis. However, the details provided still do not clearly explain how the Z-scores were calculated from the correlation. We recommend either including a few additional sentences in the methods section to explain this point explicitly or providing more documentation in the notebooks.

We thank the reviewer for this suggestion. We have opted to include additional details (underlined below) in the Methods (section **Genomic data co-variation and loop data analysis**), providing a more formal description of how these Z-scores values are calculated from an empirical approach.
"Let *P* and *G* be the number of DA-peaks (P) or DE-genes (G) observed for a particular stimulation/time condition. *P* and *G* are Poisson distributed, and for high mean values (>100) their distribution can be approximated with a Normal distribution. Thus, we can assess the deviation from expectation by using an empirical permutation approach, in which we compare our observed counts against the mean and standard deviation estimated from *P* and *G* values from permuted data obtained by shuffling the gene / peak labels.

Our permutation approach for DA-genes and DA-peaks only resamples features (genes / peaks) and maintains the distribution of observed log2 fold change and *P*-values for each time point. This permuted distribution maintains time dependencies and is less biased than full numerical shuffling. We did 1,000 permutations to obtain the expected mean (mu) and standard deviation (s) values for each *P* and *G* distributions, and used those to calculate Z-scores for the observed values of *P* or G (x), that is, Z = (x - mu) / s."

Reviewer #3:

The authors addressed most of my points of criticism and the manuscript has been improved. However, I still have a few final concerns that require revision before publication.

Specific points:

**1.** I have a concern regarding new Figure 4d,e and Appendix Figure S7c. According to Figure S7c, variation of Arc inducibility in control clones is quite big, raising a concern regarding authors' claim that enhancer KO reduces Arc inducibility. Indeed, the median inducibility of two KO clones (C5, E11) is higher than wild-type R1 clone. I recommend to move Figure 4d,e to supplementary materials and tone down related conclusions in the abstract and main text.

Thank you for this recommendation. As the reviewer pointed out, KO clones of C5 and E11 induced a higher Arc gene expression than wild-type R1 clone. These could be potentially related to the additional deletion of HIC1 repressor motifs in C5 and E11 KO lines as shown in Appendix Figure S8b (Sanger sequencing results of the CRISPR target site in each clone). As we are unable to make a solid conclusion about HIC1 function with available clones, we tone down the statements related to this experimental validation in the abstract and main text (see also points 2 and 3) and move Figures 4d,e to supplementary (**Appendix Figure S8c,d**).

**2.** The authors added the sentence: 'Deleting these TF motifs did not show any effect on Arc gene expression upon KCl stimulation (Fig. 4d)' in the new manuscript. However, Arc is not induced even in control in vitro neuronal differentiated ESCs.

The reviewer is correct that the Arc gene is not induced in vitro mouse ESC-derived neurons upon KCl stimulation. We removed the related sentence from the manuscript (purple, strike-through text):

"We observed a significant reduction in Arc gene expression upon BDNF stimulation in clones with a deletion of the distal TF motifs (t-stat = -3.0, P < 0.01; two-sided t-test), but not in CRISPR controls (P > 0.05) (**Appendix figure S8c**; **Dataset EV3**). ~~Deleting these TF motifs did not show any effect on Arc gene expression upon KCl stimulation (**Fig. 4d**).~~"

**3.** Related to this, even though the authors showed that Arc induction by KCl treatment is modest as compared with Bdnf treatment in ex vivo cultured cortical neurons (Figure 1), the complete lack of Arc induction by KCl treatment in neuronal differentiated ESCs is unclear. This raises a concern whether KCl-mediated depolarization can indeed provide a sufficient stimulus to induce downstream genes in neuronal differentiated ESCs. Because KCl experiments in Figure 4d do not provide essential additional information, these data may be removed from the panel.

We thank the reviewer for this insight and suggestion. We removed the KCl data from the previous Figure 4d, moved it to supplementary (Appendix Figure S8c), and edited text (purple/underlined):

"We observed a significant reduction in Arc gene expression upon BDNF stimulation in clones with a deletion of the distal TF motifs (t-stat = -3.0, P < 0.01; two-sided t-test), but not in CRISPR controls (P > 0.05) (**Appendix Figure S8c**; **Dataset EV3**). KCl treatment failed to induce Arc expression in mESC-derived neurons, unable to verify the role of this putative enhancer in KCl-dependent Arc expression. These results suggest that an EGR motif close to bZIP in the distal regulatory element functions in BDNF-mediated Arc gene activation."

**4.** There is a possibility that KCl treatment may cause Bdnf release, and Bdnf may also affect neuronal excitability. In the Discussion section, the authors should discuss about this potential confound.

The reviewer raised a very interesting point. We added the following sentence to the Discussion (purple/underlined): "Besides, it is possible that neuronal activation by KCl depolarization also induces BDNF expression and its release to the postsynaptic cell, where the additional response to BDNF could confound the specific effect of KCl".

**5.** I think that information about Fos and Egr1 antibodies is missing.

All specific information regarding antibodies can be found in the *Reagents and Tools* table.

RE: MSB-2021-10473RR, Comparative chromatin accessibility upon BDNF stimulation delineates neuronal regulatory elements

Thank you again for sending us your revised manuscript. We have now evaluated the changes made, and we think that the remaining concerns of the reviewers have been addressed. As such, I am pleased to inform you that your paper has been accepted for publication.

## EMBO Press Author Checklist

| | |
|---|---|
| Corresponding Author Name: Kyung-Min Noh | |
| Journal Submitted to: Molecular Systems Biology | |
| Manuscript Number: MSB-2021-10473 | |

**USEFUL LINKS FOR COMPLETING THIS FORM**
The EMBO Journal - Author Guidelines
EMBO Reports - Author Guidelines
Molecular Systems Biology - Author Guidelines
EMBO Molecular Medicine - Author Guidelines

**Reporting Checklist for Life Science Articles (updated January 2022)**
This checklist is adapted from Materials Design Analysis Reporting (MDAR) Checklist for Authors. MDAR establishes a minimum set of requirements in transparent reporting in the life sciences (see Statement of Task: 10.31222/osf.io/9sm4x). Please follow the journal's guidelines in preparing your manuscript.
**Please note that a copy of this checklist will be published alongside your article.**

**Abridged guidelines for figures**
**1. Data**
The data shown in figures should satisfy the following conditions:
- the data were obtained and processed according to the field's best practice and are presented to reflect the results of the experiments in an accurate and unbiased manner.
- ideally, figure panels should include only measurements that are directly comparable to each other and obtained with the same assay.
- plots include clearly labeled error bars for independent experiments and sample sizes. Unless justified, error bars should not be shown for technical replicates.
- if n<5, the individual data points from each experiment should be plotted. Any statistical test employed should be justified.
- Source Data should be included to report the data underlying figures according to the guidelines set out in the authorship guidelines on Data Presentation.

**2. Captions**
Each figure caption should contain the following information, for each panel where they are relevant:
- a specification of the experimental system investigated (eg cell line, species name).
- the assay(s) and method(s) used to carry out the reported observations and measurements.
- an explicit mention of the biological and chemical entity(ies) that are being measured.
- an explicit mention of the biological and chemical entity(ies) that are altered/varied/perturbed in a controlled manner.
- the exact sample size (n) for each experimental group/condition, given as a number, not a range;
- a description of the sample collection allowing the reader to understand whether the samples represent technical or biological replicates (including how many animals, litters, cultures, etc.).
- a statement of how many times the experiment shown was independently replicated in the laboratory.
- definitions of statistical methods and measures:
  - common tests, such as t-test (please specify whether paired vs. unpaired), simple χ2 tests, Wilcoxon and Mann-Whitney tests, can be unambiguously identified by name only, but more complex techniques should be described in the methods section;
  - are tests one-sided or two-sided?
  - are there adjustments for multiple comparisons?
  - exact statistical test results, e.g., P values = x but not P values < x;
  - definition of 'center values' as median or average;
  - definition of error bars as s.d. or s.e.m.

**Please complete ALL of the questions below.**
**Select "Not Applicable" only when the requested information is not relevant for your study.**

**Materials**

| Newly Created Materials | Information included in the manuscript? | In which section is the information available? (Reagents and Tools Table, Materials and Methods, Figures, Data Availability Section) |
|---|---|---|
| New materials and reagents need to be available; do any restrictions apply? | Not Applicable | |

| Antibodies | Information included in the manuscript? | In which section is the information available? (Reagents and Tools Table, Materials and Methods, Figures, Data Availability Section) |
|---|---|---|
| For **antibodies** provide the following information:<br>- Commercial antibodies: RRID (if possible) or supplier name, catalogue number and or/clone number<br>- Non-commercial: RRID or citation | Yes | Reagents and Tools Table |

| DNA and RNA sequences | Information included in the manuscript? | In which section is the information available? (Reagents and Tools Table, Materials and Methods, Figures, Data Availability Section) |
|---|---|---|
| **Short novel DNA or RNA including primers, probes**: provide the sequences. | Yes | Dataset EV5 |

| Cell materials | Information included in the manuscript? | In which section is the information available? (Reagents and Tools Table, Materials and Methods, Figures, Data Availability Section) |
|---|---|---|
| **Cell lines:** Provide species information, strain. Provide accession number in repository **OR** supplier name, catalog number, clone number, and/**OR** RRID. | Yes | Reagents and Tools Table |
| **Primary cultures:** Provide species, strain, sex of origin, genetic modification status. | Yes | Reagents and Tools Table |
| Report if the cell lines were recently **authenticated** (e.g., by STR profiling) and tested for mycoplasma contamination. | Yes | Materials and Methods |

| Experimental animals | Information included in the manuscript? | In which section is the information available? (Reagents and Tools Table, Materials and Methods, Figures, Data Availability Section) |
|---|---|---|
| **Laboratory animals or Model organisms:** Provide species, strain, sex, age, genetic modification status. Provide accession number in repository **OR** supplier name, catalog number, clone number, **OR** RRID. | Yes | Reagents and Tools Table, Materials and Methods |
| **Animal observed in or captured from the field:** Provide species, sex, and age where possible. | Not Applicable | |
| Please detail **housing and husbandry conditions**. | Yes | Materials and Methods |

| Plants and microbes | Information included in the manuscript? | In which section is the information available? (Reagents and Tools Table, Materials and Methods, Figures, Data Availability Section) |
|---|---|---|
| **Plants:** provide species and strain, ecotype and cultivar where relevant, unique accession number if available, and source (including location for collected wild specimens). | Not Applicable | |
| **Microbes:** provide species and strain, unique accession number if available, and source. | Not Applicable | |

| Human research participants | Information included in the manuscript? | In which section is the information available? (Reagents and Tools Table, Materials and Methods, Figures, Data Availability Section) |
|---|---|---|
| If collected and within the bounds of privacy constraints report on age, sex and gender or ethnicity for all study participants. | Not Applicable | |

| Core facilities | Information included in the manuscript? | In which section is the information available? (Reagents and Tools Table, Materials and Methods, Figures, Data Availability Section) |
|---|---|---|
| If your work benefited from core facilities, was their service mentioned in the acknowledgments section? | Yes | Acknowledgements |

**Design**

| Study protocol | Information included in the manuscript? | In which section is the information available? (Reagents and Tools Table, Materials and Methods, Figures, Data Availability Section) |
|---|---|---|
| If study protocol has been **pre-registered, provide DOI in the manuscript**. For clinical trials, provide the trial registration number **OR** cite DOI. | Not Applicable | |
| Report the **clinical trial registration number** (at ClinicalTrials.gov or equivalent), where applicable. | Not Applicable | |

| Laboratory protocol | Information included in the manuscript? | In which section is the information available? (Reagents and Tools Table, Materials and Methods, Figures, Data Availability Section) |
|---|---|---|
| Provide DOI OR other citation details if **external detailed step-by-step protocols** are available. | Not Applicable | |

| Experimental study design and statistics | Information included in the manuscript? | In which section is the information available? (Reagents and Tools Table, Materials and Methods, Figures, Data Availability Section) |
|---|---|---|
| Include a statement about **sample size** estimate even if no statistical methods were used. | Not Applicable | |
| Were any steps taken to minimize the effects of subjective bias when allocating animals/samples to treatment (e.g. **randomization procedure**)? If yes, have they been described? | Not Applicable | |
| Include a statement about **blinding** even if no blinding was done. | Not Applicable | |
| Describe **inclusion/exclusion criteria** if samples or animals were excluded from the analysis. Were the criteria pre-established?<br><br>If sample or data points were omitted from analysis, report if this was due to attrition or intentional exclusion and provide justification. | Not Applicable | |
| For every figure, are **statistical tests** justified as appropriate? Do the data meet the assumptions of the tests (e.g., normal distribution)? Describe any methods used to assess it. Is there an estimate of variation within each group of data? Is the variance similar between the groups that are being statistically compared? | Yes | Results, Figure Legends, Materials and Methods |

| Sample definition and in-laboratory replication | Information included in the manuscript? | In which section is the information available? (Reagents and Tools Table, Materials and Methods, Figures, Data Availability Section) |
|---|---|---|
| In the figure legends: state number of times the experiment was **replicated** in laboratory. | Not Applicable | |
| In the figure legends: define whether data describe **technical or biological replicates**. | Yes | Figure Legends, Materials and Methods |

## Ethics

| Ethics | Information included in the manuscript? | In which section is the information available? (Reagents and Tools Table, Materials and Methods, Figures, Data Availability Section) |
|---|---|---|
| Studies involving **human participants**: State details of **authority granting ethics approval** (IRB or equivalent committee(s), provide reference number for approval. | Yes | Materials and Methods |
| Studies involving **human participants**: Include a statement confirming that **informed consent** was obtained from all subjects and that the experiments conformed to the principles set out in the WMA Declaration of Helsinki and the Department of Health and Human Services Belmont Report. | Not Applicable | |
| Studies involving **human participants:** For publication of **patient photos**, include a statement confirming that consent to publish was obtained. | Not Applicable | |
| Studies involving experimental **animals**: State details of **authority granting ethics approval** (IRB or equivalent committee(s), provide reference number for approval. Include a statement of compliance with ethical regulations. | Yes | Materials and Methods |
| Studies involving **specimen and field samples:** State if relevant **permits** obtained, provide details of authority approving study; if none were required, explain why. | Not Applicable | |

| Dual Use Research of Concern (DURC) | Information included in the manuscript? | In which section is the information available? (Reagents and Tools Table, Materials and Methods, Figures, Data Availability Section) |
|---|---|---|
| Could your study fall under dual use research restrictions? Please check biosecurity documents and list of **select agents and toxins** (CDC): https://www.selectagents.gov/sat/list.htm | Not Applicable | |
| If you used a select agent, is the security level of the lab appropriate and reported in the manuscript? | Not Applicable | |
| If a study is subject to dual use research of concern regulations, is the name of the **authority granting approval and reference number** for the regulatory approval provided in the manuscript? | Not Applicable | |

## Reporting

The MDAR framework recommends adoption of discipline-specific guidelines, established and endorsed through community initiatives. Journals have their own policy about requiring specific guidelines and recommendations to complement MDAR.

| Adherence to community standards | Information included in the manuscript? | In which section is the information available? (Reagents and Tools Table, Materials and Methods, Figures, Data Availability Section) |
|---|---|---|
| State if relevant guidelines or checklists (e.g., **ICMJE, MIBBI, ARRIVE, PRISMA**) have been followed or provided. | Not Applicable | |
| For **tumor marker prognostic studies,** we recommend that you follow the **REMARK** reporting guidelines (see link list at top right). See author guidelines, under 'Reporting Guidelines'. Please confirm you have followed these guidelines. | Not Applicable | |
| For **phase II and III randomized controlled trials**, please refer to the **CONSORT** flow diagram (see link list at top right) and submit the CONSORT checklist (see link list at top right) with your submission. See author guidelines, under 'Reporting Guidelines'. Please confirm you have submitted this list. | Not Applicable | |

## Data Availability

| Data availability | Information included in the manuscript? | In which section is the information available? (Reagents and Tools Table, Materials and Methods, Figures, Data Availability Section) |
|---|---|---|
| Have **primary datasets** been deposited according to the journal's guidelines (see 'Data Deposition' section) and the respective accession numbers provided in the Data Availability Section? | Yes | Data Availability Section |
| Were **human clinical and genomic datasets** deposited in a public access-controlled repository in accordance to ethical obligations to the patients and to the applicable consent agreement? | Not Applicable | |
| Are **computational models** that are central and integral to a study available without restrictions in a machine-readable form? Were the relevant accession numbers or links provided? | Not Applicable | |
| If publicly available data were reused, provide the respective **data citations in the reference list**. | Yes | Reference List |
